# Three-Component Reaction of 3-Formyl-6-Methylchromone, Primary Amines, and Secondary Phosphine Oxides: A Synthetic and Mechanistic Study

**DOI:** 10.1021/acsomega.2c07333

**Published:** 2022-12-30

**Authors:** Nóra Popovics-Tóth, Trinh Dang Tran Bao, Ádám Tajti, Béla Mátravölgyi, Zsolt Kelemen, Franc Perdih, László Hackler, László G. Puskás, Erika Bálint

**Affiliations:** † Department of Organic Chemistry and Technology, Budapest University of Technology and Economics, Budafoki út 8., H-1111 Budapest, Hungary; ‡ Department of Inorganic and Analytical Chemistry, Budapest University of Technology and Economics, Szent Gellért tér 4., H-1111 Budapest, Hungary; § Faculty of Chemistry and Chemical Technology, University of Ljubljana, SI-1000 Ljubljana, Slovenia; ∥ Anthelos Ltd., Alsó kikötő sor 11/D, H-6726 Szeged, Hungary

## Abstract

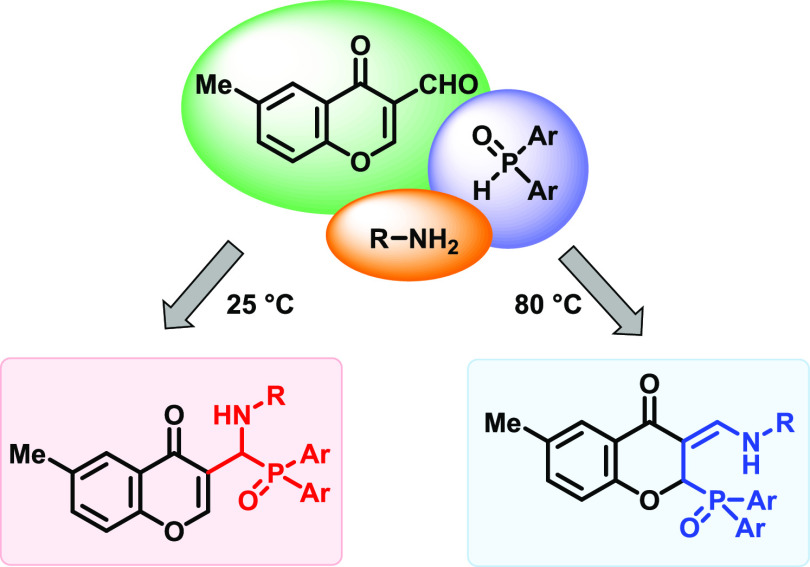

A fast, mild, and efficient catalyst-free approach has been developed for the synthesis of chromonyl-substituted α-aminophosphine oxides by the three-component reaction of 3-formyl-6-methylchromone, primary amines, and secondary phosphine oxides at ambient temperature. Carrying out the reaction with aliphatic amines or aminoalcohols at a higher temperature (80 °C), phosphinoyl-functionalized 3-aminomethylene chromanones were formed instead of the corresponding chromonyl-substituted α-aminophosphine oxides. No reaction occurred when 3-formyl-6-methylchromone and secondary phosphine oxides were reacted with aromatic amines in the absence of any catalyst. Applying a basic catalyst, the formation of the phosphinoyl-functionalized 3-aminomethylene chromanones was observed; however, the reaction was not complete. Detailed experimental and quantum chemical studies were performed to study the transformation. Moreover, the *in vitro* cytotoxicity of phosphinoyl-functionalized 3-aminomethylene chromanones was also investigated in three different cell lines, such as human lung adenocarcinoma (A549), mouse fibroblast (NIH/3T3), and human promyelocytic leukemia (HL60) cells. Several derivatives showed modest activity against the human promyelocytic leukemia (HL60) cell line.

## 1 Introduction

In the last few decades, multicomponent reactions (MCRs) became widely applied in the field of small-molecule drug discovery.^1^ Starting from simple and cheap reagents, a large library of structurally related compounds can be synthesized in a short time.^2^ In general, MCRs have high atom efficiency, which saves time and energy.^3^

One of the most important groups of organic compounds is heterocyclic derivatives. They play an important role in the pharmaceutical and plastic industries and in agriculture.^4^ Among heterocyclic compounds, *O*-heterocycles are found in the structure of naturally occurring vitamins, hormones, antibiotics, sugars, and pigments.^5^ The synthetic derivatives can also have great pharmaceutical significance, as they have antitumor, antiviral, antimicrobial, or anti-inflammatory activities.^6^ In addition, some of them may also have photochemical properties.^7^

3-Formylchromone, as an interesting starting material in MCRs, has three different electron-deficient centers that can be attacked by various nucleophiles, thereby providing an opportunity to create a wide range of new chromone derivatives.^8^ In addition, intramolecular ring opening can also take place, and further heterocycles can be formed.^9^

Several derivatives containing a chromone backbone have already been synthesized; however, only a few chromones containing a phosphine oxide or a phosphonate moiety can be found in the literature. Some of them have been applied as flame-retardant agents in polymers^10^ or have antioxidant, antimicrobial,^11^ or anticancer effects (against antitumor HepG2 (human liver) and HFB4 (human normal melanocyte) cell lines).^12^

A few chromonyl α-aminophosphonates were prepared by the three-component Kabachnik–Fields reaction of 3-formylchromones, aromatic amines, and dialkyl phosphites.^11,13^ The condensations were carried out without a catalyst in a solvent or in the absence of any solvent, at 70–110 °C for long reaction times (2–6 h), and the desired chromonyl α-aminophosphonates were obtained in variable yields (16–70%). In another example, 3-formylchromone derivatives, 3-amino-2-phenyl-quinazolin-4(3*H*)-one, and 1.5 equivalents of diethyl phosphite were reacted in a “one-pot” method or in two steps, where a Schiff base was formed first and then it reacted with the phosphorus reagent.^12^ Additional aminophosphonates were synthesized by the Kabachnik–Fields reaction of 3-formylchromones, amide derivatives, and trialkyl phosphites at 80–100 °C for 1–1.5 h in acetic acid as the solvent.^14,15^

In one case, the condensation of 3-formylchromone, bifunctional amines (aminoalcohols or diamines) or urea derivatives, and dialkyl phosphites was investigated, where a ring opening was observed, and new five-, six- or seven-membered heterocyclic units were formed.^16^

In this paper, the three-component reaction of 3-formyl-6-methylchromone, primary amines, and secondary phosphine oxides was investigated in the absence of any catalyst or using a basic catalyst. We aimed at the optimization of a model reaction, and to extend it using various amines and phosphorus reagents. We also wished to study the mechanism of the three-component reaction by experiments, as well as by quantum chemical calculations. The *in vitro* cytotoxicity of several compounds synthesized was also aimed to be investigated in three different cell lines (human lung adenocarcinoma cell line (A549), mouse fibroblast healthy cell line (NIH/3T3), and human promyelocytic leukemia cell line (HL60)).

## 2 Results and Discussion

### 2.1 Three-Component Reaction of 3-Formyl-6-methylchromone, Primary Amines, and Secondary Phosphine Oxides

**Figure 1:**
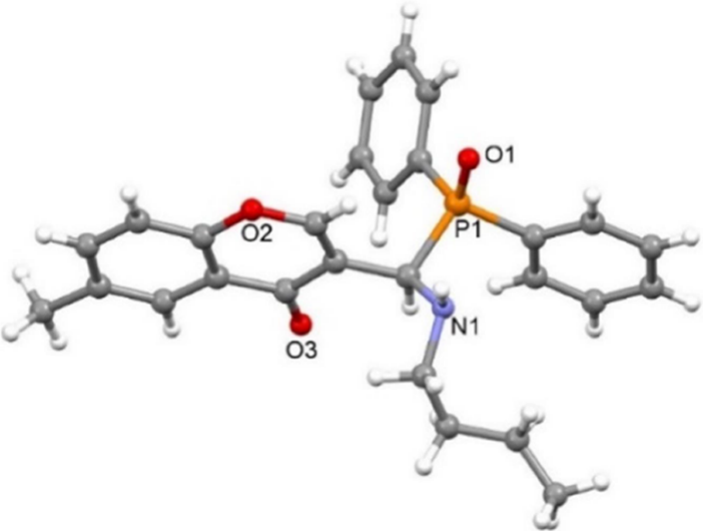
Molecular structure of **1a**.

The catalyst-free Kabachnik–Fields reaction of 3-formyl-6-methylchromone, butylamine and diphenylphosphine oxide (DPPO) was studied with respect to the heating mode, temperature, and reaction time (Table 1). First, the condensation was carried out in acetonitrile, at 80 °C for 1 h in an oil bath. The conversion was not complete, and surprisingly beside the corresponding chromonyl-substituted α-aminophosphine oxide (**1a**), a phosphinoyl-functionalized 3-(butylamino)methylene chromanone (**2a**) was formed as the major component (Table 1, entry 1). When repeating the reaction under microwave (MW) heating, the conversion was complete; however, the proportion did not change (Table 1, entry 2). After column chromatography, the phosphinoyl-functionalized 3-(butylamino)methylene chromanone (**2a**) was obtained in a yield of 86%. In order to increase the ratio of the α-aminophosphine oxide product (**1a**), the temperature was decreased to 60 °C, and it was found that the ratio of products **1a** and **2a** was reversed (64:36) (Table 1, entry 3). When carrying out the reaction at room temperature for 1 h without MW irradiation, the ratio of product **1a** increased to 76%, which did not change further after longer reaction time (4 h) (Table 1, entries 4 and 5) or at lower temperature (0 °C), where the conversion was lower, but the product composition was similar (Table 1, entry 6). After 4 h reaction time at ambient temperature and column chromatography, **1a** was isolated in a yield of 49%. By slow evaporation of the acetonitrile solution of compound **1a**, single crystals suitable for the X-ray diffraction (XRD) study were obtained, which revealed the molecular structure of compound **1a** (Figure 1). In the crystal lattice, the amino group of **1a** molecules is involved in the formation of hydrogen bonded dimer units via centrosymmetric N–H···O=P interaction (Figure S1, Table S1 in the Supporting Information). These dimers are further connected into a chain along the *c* axis through π···π interactions between two adjacent chromenyl C4–C9 rings with a centroid-to-centroid distance of 3.683 Å (Figure S2).

**Table 1:**
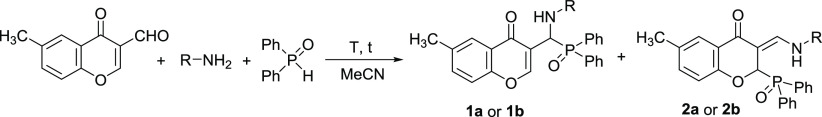
Catalyst-Free Reaction of 3-Formyl-6-methylchromone, Butylamine or Aniline, and Diphenylphosphine Oxide^a^

						product composition^b^ [%]		
entry	R	mode of heating	*T* [°C]	*t* [h]	conversion^b^ [%]	**1a** (R = Bu) or **1b** (R = Ph)	**2a** (R = Bu) or **2b** (R = Ph)	yield^c^ [%]
**1**	Bu	Δ	80	1	90	7	93	
**2**	Bu	MW	80	1	100	7	93	86 (**2a**)
**3**	Bu	MW	60	1	98	64	36	
**4**	Bu	-	25	1	55	76	24	
**5**	Bu	-	25	4	100	76	24	49 (**1a**)
**6**	Bu	-	0	1	29	68	32	
**7**	Ph	-	25	0.5	82	100	0	
**8**	Ph	-	25	1	100	100	0	94 (**1b**)
**9**	Ph	MW	80	1	100	100	0	
**10**	Ph	MW	120	1	100	100	0	

a Reactants: 3-formyl-6-methylchromone (1 mmol), butylamine or aniline (1 mmol), diphenylphosphine oxide (1 mmol).

b Determined by ^31^P NMR.

c Isolated yield.

After that, the catalyst-free reaction of 3-formyl-6-methylchromone, aniline, and diphenylphosphine oxide was studied (Table 1, entries 7–9). Performing the condensation at room temperature for 30 min without MW irradiation, the conversion was already 82%, and the chromonyl-substituted α-aminophosphine oxide (**1b**) was formed selectively (Table 1, entry 7). Increasing the reaction time to 1 h, no diphenylphosphine oxide remained in the mixture, and product **1b** was isolated in a yield of 94% (Table 1, entry 8). In order to investigate the formation of the phosphinoyl-functionalized 3-(phenylamino)methylene chromanone (**2b**), the reaction was also carried out at 80 °C for 1 h in an MW reactor; however, no product **2b** was detected even at a higher temperature of 120 °C (Table 1, entries 9 and 10).

In the next part, the reaction of 3-formyl-6-methylchromone, aniline, and diphenylphosphine oxide was carried out in the presence of basic catalysts to investigate the formation of the phosphinoyl-functionalized 3-(phenylamino)methylene chromanone (**2b**) (Table 2). When using 10 mol % of pentamethyldiethylenetriamine (PMDTA) as the catalyst and performing the reaction in acetonitrile at 80 °C for 1 h in an MW reactor, the conversion was almost complete (98%), and the mixture mainly contained chromonyl-substituted α-hydroxyphosphine oxide (**3**) (50%); however, besides the chromonyl-substituted α-aminophosphine oxide (**1b**), 28% of 3-(phenylamino)methylene chromanone derivative (**2b**) was also formed (Table 2, entry 1). By increasing the amount of PMDTA to 20 mol %, the ratio of products **1b** and **2b** increased to 24 and 34%, respectively (Table 2, entry 2). When switching to Hünig’s base (*N,N*-diisopropylethylamine, DIPEA) and repeating the reaction at 80 °C for 1 h, the conversion was 90%; however, the ratio of compounds **1b**, **2b**, and **3** changed to 47:30:13, which means that in this case, the formation of the α-aminophosphine oxide derivative (**1b**) was more favorable (Table 2, entry 3). When the condensation was performed in the presence of 20 mol % of DIPEA at a higher temperature (100 °C) and for a longer reaction time (2 h), a complete conversion was achieved, and the phosphinoyl-functionalized 3-(phenylamino)methylene chromanone (**2b**) was the main component (54%) (Table 2, entry 4). One can conclude that when using aniline in the three-component reaction, (*Z*)-3-[(amino)methylene]-2-(diphenylphosphoryl)-6-methylchroman-4-one (**2b**) can only be synthesized under harsher conditions, in the presence of a basic catalyst at a higher temperature with a longer reaction time, however, not selectively.

**Table 2:**
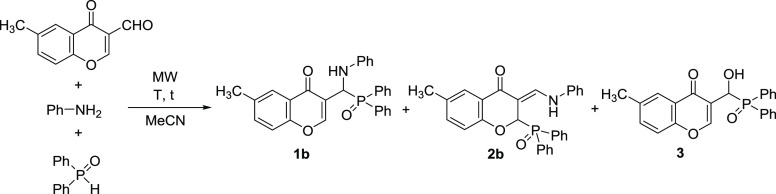
MW-Assisted Three-Component Reaction of 3-Formyl-6-methylchromone, Aniline, and Diphenylphosphine Oxide in the Presence of a Base^a^

				composition^b^ [%]			
entry	catalyst	*T* [°C]	*t* [h]	DPPO	**1b**	**2b**	**3**
1	PMDTA (10 mol %)	80	1	2	20	28	50
2	PMDTA (20 mol %)	80	1	0	24	34	42
3	DIPEA (10 mol %)	80	1	10	47	30	13
4	DIPEA (20 mol %)	100	2	0	33	54	13

a Reactants: 3-Formyl-6-methylchromone (1 mmol), aniline (1 mmol), and diphenylphosphine oxide (1 mmol).

b Determined by ^31^P NMR.

**Scheme 1:**
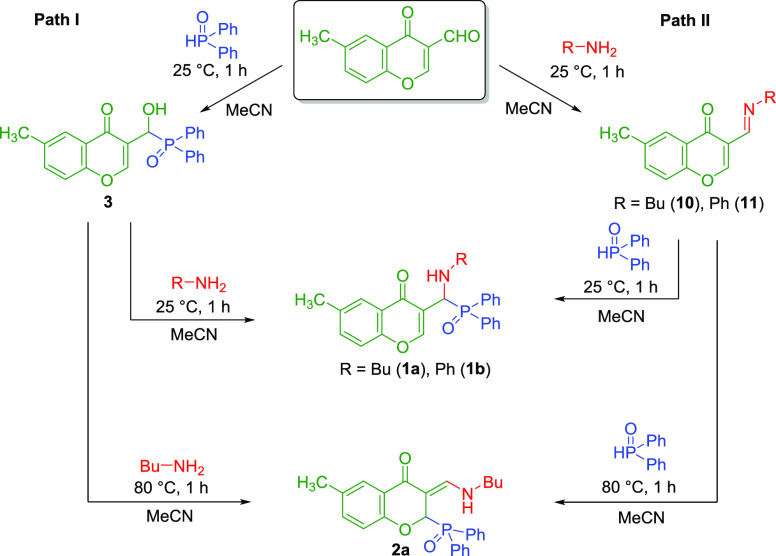
Possible Reaction Paths to **1a**/**1b** and **2a**

During the investigation of the mechanism of the formation of products **1a**/**1b** and **2a**/**2b**, it could be established that two different reaction paths (Path I and Path II) could be considered, as depicted in Scheme 1.

In order to get a deeper insight into the mechanism (Scheme 1), different intermediates involved in the postulated reaction paths were synthesized. Moreover, density functional theory (DFT) calculations were performed to support our findings and help to understand the reaction mechanism at the ωB97X-D/6-31G* level (more details and comparison of different levels of theory in the SI).

First, the thermodynamic stability of compounds **1a**, **1b** and **2a**, **2b** was calculated. According to our DFT calculations at the ωB97X-D/6-31G* level, the energy difference between the two isomers is tiny. **2a** is more stable by 1.0 kcal/mol than **1a**; in contrast, **2b** is less stable by 1.1 kcal/mol than **1b**. Application of different levels of theory does not give significant differences in energy (Table S2). It is important to mention that these energy differences are small (they are actually below the accuracy of the theoretical level); therefore, no significant thermodynamic control could be expected. The direct (monomolecular) transformation of **1a** to **2a** and that of **1b** to **2b** were calculated, and as it can be expected, monomolecular barriers are extremely high (120.0 and 80.1 kcal/mol, respectively). It is in agreement with the high stability of the isolated solid compound (**1b**), which was heated until 120 °C, and no decomposition or change was observed based on ^31^P NMR signals.

**Scheme 2:**
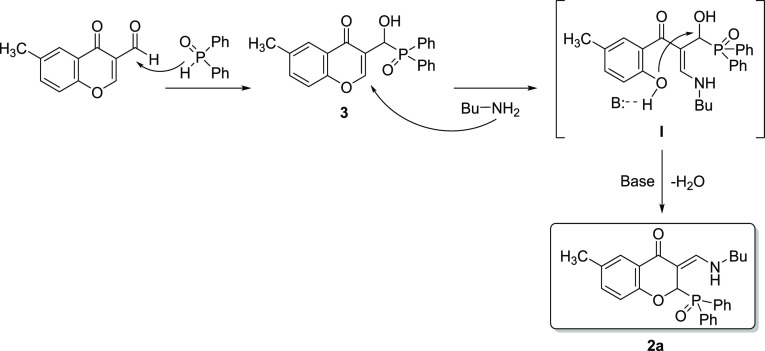
Mechanism of the Formation of (*Z*)-3-[(Butylamino)methylene]-2-(diphenylphosphoryl)-6-methylchroman-4-one (**2a**)

After the evaluation of the thermodynamic situation and exclusion of the direct transformation of **1a/2a** to **1b/2b**, the reaction of 3-formyl-6-methylchromone with diphenylphosphine oxide was carried out (Scheme 1/Path I). Complete conversion was achieved in acetonitrile at room temperature after 1 h, and the corresponding chromonyl-substituted α-hydroxyphosphine oxide (**3**) was formed in a yield of 98%. After that, derivative **3**, which can be considered as an intermediate of the three-component reaction, was reacted further with aniline at room temperature for 1 h. This reaction was complete, and only the desired chromonyl-substituted α-aminophosphine oxide (**1b**) was formed. A similar reaction with butylamine (at 25 °C for 1 h) gives compound **1a** as the main product; however, the conversion was only 58%, which did not change significantly when the reaction was allowed to stir for a longer time. When repeating this reaction at a higher temperature (80 °C for 1 h), the 2-phosphinoyl- and 3-(butylamino)methylene-substituted 6-methylchroman-4-one derivative (**2a**) was obtained with a conversion of 93%, while a similar reaction with aniline did not give the corresponding **2b** product. One noticeable difference between the two reactions sets is the amine’s basicity, which seems crucial toward the products **2a** and **2b**. Our DFT calculations further supported this finding, and the postulated mechanism of the formation of **2a** and **2b** is shown in Scheme 2. The nucleophilic attack of the amine and the ring opening step has a high barrier in both cases (61.3 and 73.9 kcal/mol, respectively), and in the transition states, the H changes its position from the amine nitrogen toward the ring’s oxygen. Usually, the transition state of proton migration could be stabilized by polar solvents and by the presence of a coordinative water molecule (it is a byproduct of the reaction); therefore, a lower barrier could be expected for the real system, where solvent-assisted proton transport may significantly reduce this barrier (which was calculated without coordinative water/solvent molecules in a recent study).

The following step is the nucleophilic attack of the phenolic −OH group. All attempts to localize the corresponding transition states failed in our hand, and further scan calculations showed that the reaction could not proceed this way. During these scan calculations, the distance between the phenolic oxygen atom and the α-carbon atom was gradually decreased by 0.1 Å, at each bond length the geometry of the structure was optimized, and the energy of the system was investigated. The energy of the system increased strictly monotonic, without any local maxima, which indicates that no local minima could be expected for an intermediate, in which the phenolic oxygen was bonded to the α-carbon atom. On the other hand, after deprotonation of the acidic phenolic −OH group, and calculating the system as an anion, the attack of the −O^–^ to the α-carbon atom is barrierless. This simple model clearly indicates the crucial role of the basic conditions. While aliphatic amines, which are more basic than aniline (Δp*K*_a_ ∼ 3), provide sufficiently basic conditions, the use of less basic aniline in the reaction without any additional base renders the ring closing step impossible. In the presence of a catalytic amount of external base, the reaction proceeds smoothly due to lower energy barriers of the pathway, including deprotonation–protonation steps.

**Scheme 3:**
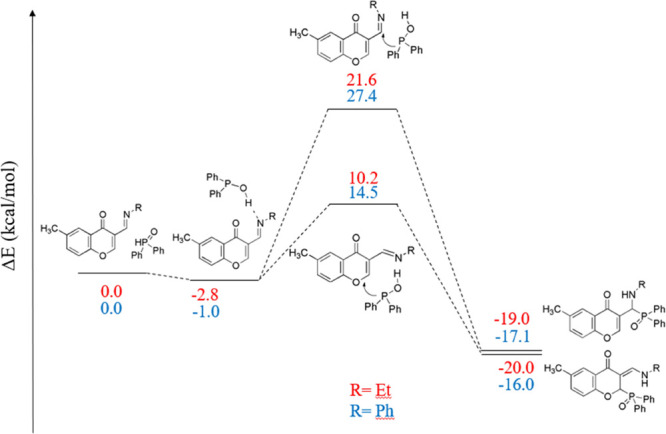
Energy Profile Computed for Addition of Diphenylphosphinous Acid to the Corresponding Schiff Bases Relative stabilities (at the ωB97XD/6-31G* level of theory) are shown in kcal/mol, with respect to the reactant state. 10 and their derivatives were calculated with ethyl substituents at the nitrogen atoms (instead of the *n*-butyl).

In the next part, we have investigated the second possible reaction route (Scheme 1/Path II). The condensation of 3-formyl-6-methylchromone and butylamine or aniline was carried out at room temperature. Schiff base **10** (R = Bu) was formed in a conversion of 74%, and Schiff base **11** (R = Ph) was obtained with a somewhat higher conversion (78%). Then, the reaction mixtures containing imine **10** or **11** were reacted further with diphenylphosphine oxide at room temperature. In both cases, the reactions were incomplete after 1 h. When starting from imine **11** only the corresponding α-aminophosphine oxide derivative (**1b**) was formed with 76% conversion; however, in case of Schiff base **10** (R = Bu) the conversion was lower (50%), and besides α-aminophosphine oxide **1a** (32%), the phosphinoyl-functionalized 3-(butylamino)methylene chromanone (**2a**) was also formed (18%). Because of the low conversion, the reaction of imine **10** and diphenylphosphine oxide was repeated at 80 °C (with 1 h reaction time). In this case, the 3-(butylamino)methylene chromanone derivative (**2a**) was formed as the major product (66% conversion). In view of these data, this reaction route (Path II, starting from the corresponding Schiff bases **10** or **11**) seems to be much slower than Path I. One possible explanation could be the significantly higher barrier of the reaction between the corresponding Schiff base and of diphenylphosphine oxide (compared to the barriers in Path I). Despite the fact that several direct additions of diphenylphosphine oxide to multiple bonds were reported,^17^ these reactions require harsher reaction conditions (high temperature or strong bases (such as KOH and *t*-BuOK)). We were not able to localize any transition state between the corresponding Schiff bases (**10** and **11**) and diphenylphosphine oxide toward compounds **1a**, **2a** and **1b**, **2b**. On the one hand, the reaction could proceed through the tautomer form of diphenylphosphine oxide (diphenylphosphinous acid).^18^ The nucleophilic attack and the migration of the proton have medium high barriers 10.2–27.4 kcal/mol (Scheme 3). On the other hand, it is important to highlight that the presence of the less stable tautomer form of diphenylphosphine oxide (even if it is stabilized by the H-bond with the amine base) is required for this reaction route. Finally, the model system was investigated in which diphenylphosphine oxide was deprotonated. In this particular case, the addition is barrierless, in agreement with the former finding in case of harsher reaction conditions.^17^ Alternatively, the corresponding Schiff bases (**10** and **11**) are able to hydrolyze to the corresponding starting compounds (aldehyde and amine), and the reaction could proceed through Path I as well. In view of our experimental and theoretical results, both reaction paths are possible.

**Scheme 4:**
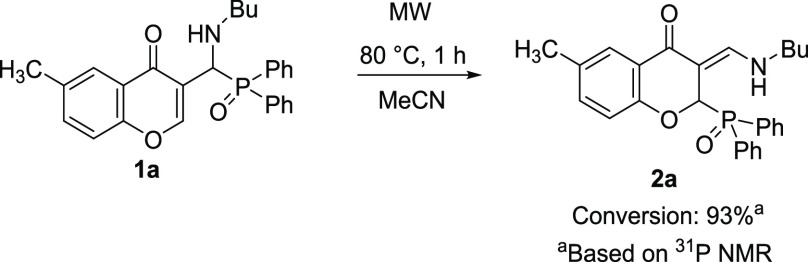
Study of the Formation of Compound **2a** from **1a**

Finally, the formation of phosphinoyl-functionalized 3-(butylamino)methylene chromanone (**2a**) was investigated from the corresponding α-aminophosphine oxide (**1a**) in acetonitrile solution (Scheme 4). The reaction mixture was heated to 80 °C for 1 h in an MW reactor. Based on ^31^P NMR spectroscopy, the (*Z*)-3-[(butylamino)methylene]-2-(diphenylphosphoryl)-6-methylchroman-4-one (**2a**) was formed in a conversion of 93%. As the direct (monomolecular) transformation was already excluded, compound **1a** should first decompose to α-hydroxyphosphine oxide **3** and butylamine in solution and then thermodynamically more stable **2a** forms (see Scheme 2).

**Scheme 5:**
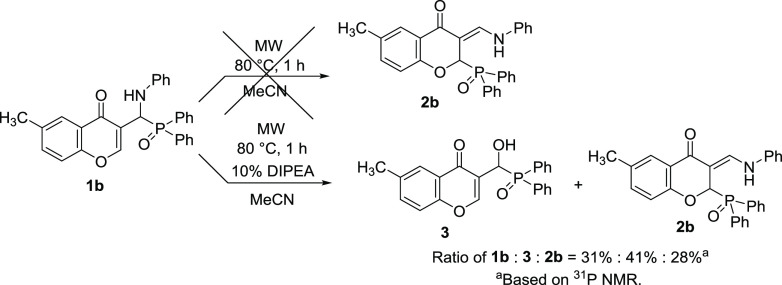
Study of the Formation of Compound **2b** from **1b**

A similar reaction with α-aminophosphine oxide **1b** was also performed in acetonitrile under the same conditions. However, the formation of the phosphinoyl-functionalized 3-(phenylamino)methylene chromanone (**2b**) was not observed at all (Scheme 5). Carrying out an experiment in the presence of 10 mol % of DIPEA, α-hydroxyphosphine oxide (**3**) and the enamine-type derivative (**2b**) were formed from the α-aminophosphine oxide (**1b**). This also confirms that the formation of the phosphinoyl-functionalized 3-(phenylamino)methylene chromanone (**2b**) is not possible without a basic catalyst, if the nitrogen atom is substituted with an aromatic moiety. Furthermore, the low conversion was in agreement with the somewhat lower (1.1 kcal/mol) stability of compound **2b** compared to **1b**.

**Scheme 6:**
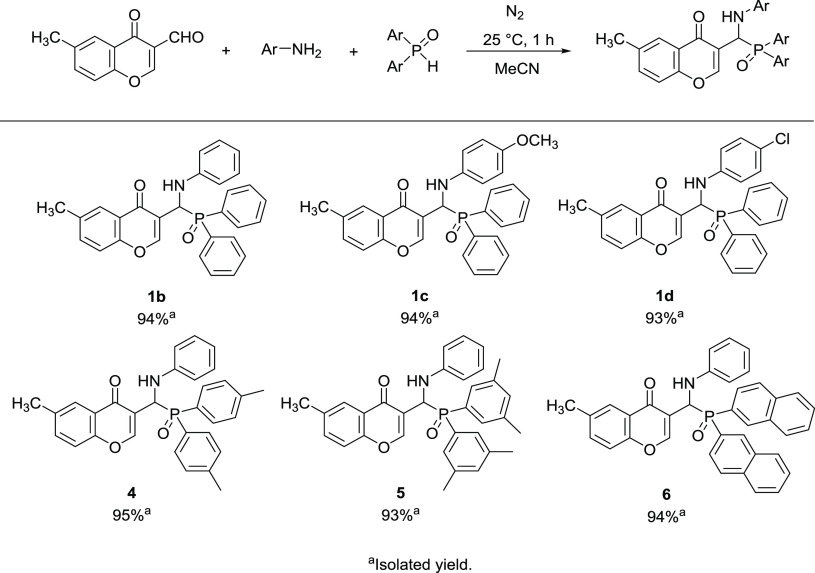
Three-component Reaction of 3-Formyl-6-methylchromone, Aromatic Amines, and Secondary Phosphine Oxides

After optimization and understanding the reaction mechanism, our aim was to synthesize a small library of structurally related new derivatives by the extension of the model reaction (Scheme 6). First, the Kabachnik–Fields reaction of 3-formyl-6-methylchromone, aromatic amines, and secondary phosphine oxides was performed under the optimized conditions (at 25 °C for 1 h, without a catalyst, in acetonitrile) found earlier (Table 1, entry 7). The condensation of 3-formyl-6-methylchromone, aniline, *p*-anisidine or 4-chloroaniline, and diphenyl phosphine oxide resulted in the corresponding 3-[(diarylphosphoryl)(phenylamino)methyl]-6-methyl-4*H*-chromen-4-one derivatives (**1b**–**d**) in excellent yields (93–94%). Neither the electron-donating methoxy group nor the electron-withdrawing chloro-substituent caused a significant difference in selectivity, leading to the formation of the α-aminophosphine-oxide derivatives (**1c** and **1d**) as the major products. 3-Formyl-6-methylchromone and aniline were also reacted with bis(*p*-tolyl)-, bis(3,5-dimethylphenyl)- or with bis(2-naphthyl)phosphine oxide, where further three new chromonyl-substituted α-aminophosphine-oxides were synthesized (**4**, **5**, and **6**) selectively in yields of 93–95%.

**Scheme 7:**
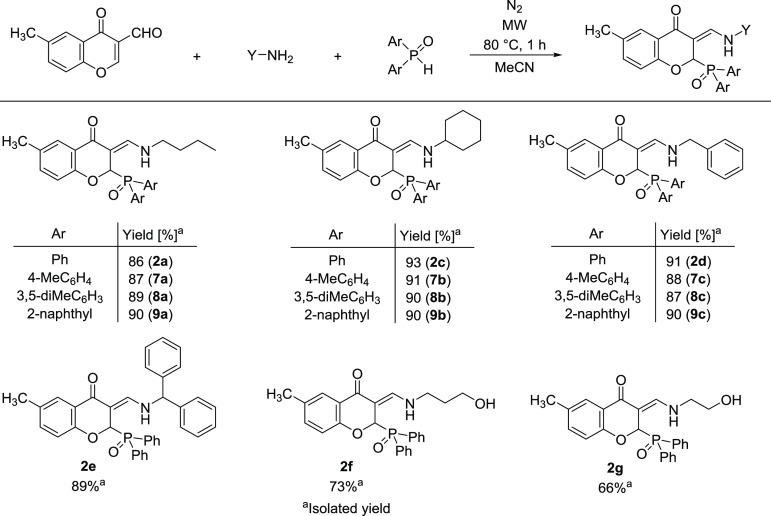
MW-Assisted Reaction of 3-Formyl-6-methylchromone, Aliphatic Amines, and Secondary Phosphine Oxides

In the next series of experiments, the catalyst-free reaction of 3-formyl-6-methylchromone, aliphatic amines (butyl-, cyclohexyl-, benzyl- or benzhydrylamine), or aminoalcohols (ethanol- or propanolamine) and various secondary phosphine oxides (diphenyl-, bis(*p*-tolyl)-, bis(3,5-dimethylphenyl)- or bis(2-naphthyl)phosphine oxide) was performed in an MW reactor at 80 °C for 1 h in acetonitrile (Scheme 7). Based on the results, in all cases, the major products were the phosphinoyl-functionalized 3-(amino)methylene chromanones (**2a**–**g**, **7a**–**c, 8a**–**c, 9a**–**c**). First, butylamine was used as the aliphatic amine, and four 3-[(butylamino)methylene]-2-(diarylphosphoryl)-6-methylchroman-4-one derivatives (**2a**, **7a**, **8a**, and **9a**) were prepared in high yields (86–90%). After that, the three-component reaction was carried out with cyclohexylamine, and the four new enamine-type derivatives (**2c**, **7c**, **8c**, and **9c**) were obtained in somewhat higher yields (90–93%). In addition, further four 3-[(benzylamino)methylene]-2-(diarylphosphoryl)-6-methylchroman-4-ones (**2d**, **7d**, **8d**, and **9d**) were synthesized using benzylamine as the amine component and were also isolated in high yields (87–91%). The reaction was also carried out with benzhydrylamine, and the target enamine-type derivative (**2e**) was prepared in a yield of 89%. Finally, aminoalcohols were applied as the amine component, which were somewhat less reactive based on the lower yields (66–73%) obtained.

As a conclusion, chromonyl-substituted α-aminophosphine oxides (**1a**–**d** and **4**–**6**) and phosphinoyl-functionalized 3-(amino)methylene chromanones (**2a**–**g** and **7a**–**c**, **8a**–**c** and **9a**–**c**) could be synthesized in two different pathways. In case of Path I, a hydroxyphosphine oxide derivative (**3**) was formed in the condensation of 3-formyl-6-methylchromone and diphenylphosphine oxide. The next step was the addition of amines, where the desired products were formed. In Path II, the first step was the reaction of 3-formyl-6-methylchromone and amines. The Schiff bases (**10** and **11**) formed were then reacted with diphenylphosphine oxide. Based on our results, the reaction was complete and faster through Path I compared to Path II.

In all, seven 3-[(amino)(diphenylphosphoryl)methyl]-6-methyl-4*H*-chromen-4-ones (**1a**–**d** and **4**–**6**) and 16 (*Z*)-3-[(amino)methylene]-2-(diarylphosphoryl)-6-methylchroman-4-one derivatives (**2a**–**g** and **7a**–**c**, **8a**–**c** and **9a**–**c**) were synthesized in good to high yields. All products are new compounds.

### 2.2 Biological Activity Studies

The *in vitro* cytotoxicity of the several synthesized compounds was also investigated. The cytotoxicity assays were carried out using the human lung adenocarcinoma (A549) cell line, mouse fibroblast (NIH/3T3) as a healthy cell line, and human promyelocytic leukemia (HL60) cell line. During the measurements, the fluorescent Resazurin assay as described previously was applied.^19^ For the A549 and NIH/3T3 cell lines, doxorubicin was the positive control (IC_50_ = 0.31 ± 0.24 and 5.65 ± 0.81 μM, respectively), while for HL60, it was bortezomib (IC_50_ = 7.42 ± 2.60 nM). The IC_50_ values (50% inhibiting concentration) obtained are shown in Table 3.

**Table 3:**
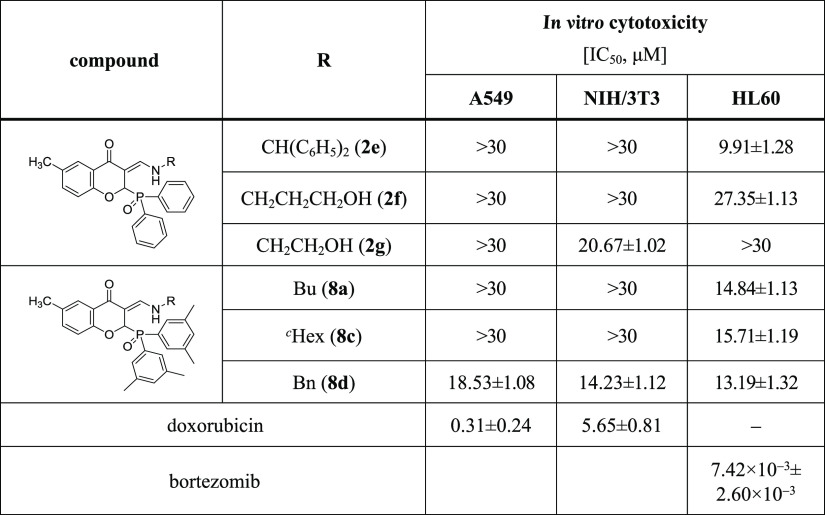
*In Vitro* Cytotoxicity of (*Z*)-3-[(Amino)methylene]-2-(diarylphosphoryl)-6-methylchroman-4-ones^a^

a Data were expressed as mean ± standard deviation.

According to the results obtained, some (*Z*)-3-[(amino)methylene]-2-(diarylphosphoryl)-6-methylchroman-4-one analogues were active. *N*-Benzhydryl- (**2e**) and *N*-(3-hydroxypropyl) derivatives (**2f**) showed moderate activity against HL60 cells (IC_50_ = 9.91 ± 1.28 and 27.35 ± 1.13 μM, respectively). (*Z*)-3-[(Ethanolamino)methylene]-2-(diphenylphosphoryl)-6-methylchroman-4-one (**2g**) was slightly effective against the mouse fibroblast (NIH/3T3) cell line (IC_50_ = 20.67 ± 1.02 μM). Furthermore, IC_50_ values of (*Z*)-3-[(amino)methylene]-2-[bis(3,5-dimethylphenyl)phosphoryl]-6-methylchroman-4-ones (**8a**, **8c**, **8d**) were in a range of 15 μM in the HL60 cell line. Compound **8d** also showed modest activity against human lung adenocarcinoma and mouse fibroblast cell lines as well (IC_50_ = 18.53 ± 1.08 and 14.23 ± 1.12 μM, respectively).

The *in vitro* cytotoxicity of (*Z*)-3-[(benzhydrylamino)methylene]-2-(diphenylphosphoryl)-6-methylchroman-4-one (**2e**) against the HL60 cell line was the most significant among all derivatives. In addition, (*Z*)-3-[(benzylamino)methylene]-2-[bis(3,5-dimethylphenyl)phosphoryl]-6-methylchroman-4-one (**8d**) was also found to be a promising candidate, due to its activity in all of the investigated cell lines (A549, NIH/3T3 and HL60).

## 3 Conclusions

In summary, we have developed a novel and practical catalyst-free method for the synthesis of new chromonyl-substituted α-aminophosphine oxides (**1a**–**d** and **4**–**6**) by the Kabachnik–Fields reaction of 6-methyl-3-formylchromone, secondary phosphine oxides, and primary amines at ambient temperature with a short reaction time. This procedure means a promising approach to attain these new heterocycles, since it applies mild and easily operational conditions (no special reagents, catalysts or additives, no heating). In addition, we have shown that by carrying out the catalyst-free three-component reaction with aliphatic amines or aminoalcohols at higher temperature (80 °C) under MW irradiation, enamine-type derivatives (**2a**–**g** and **7a**–**c**, **8a**–**c** and **9a**–**c**) were formed instead of chromonyl-substituted α-aminophosphine oxides (**1a**–**d** and **4**–**6**). The methodology was applied for the synthesis of a wide range of phosphinoyl-functionalized 3-(amino)methylene chromanones (**2a**–**g** and **7a**–**c**, **8a**–**c** and **9a**–**c**), which form a new family of compounds in the literature. In case of aromatic amines, the enamine-type derivatives could be only prepared in the presence of a base; however, this reaction was not complete. Detailed experimental and quantum chemical studies have revealed that the phosphinoyl-functionalized 3-(amino)methylene chromanone derivatives could be formed by a ring opening of the chromone ring. This transformation depends on the basicity of the amines used in the synthesis; therefore, it can easily take place with aliphatic amines. In case of aromatic amines, an additional base had to be used.

Altogether, seven 3-[(amino)(diphenylphosphoryl)methyl]-6-methyl-4*H*-chromen-4-ones (**1a**–**d** and **4**–**6**) and 16 (*Z*)-3-[(amino)methylene]-2-(diarylphosphoryl)-6-methylchroman-4-one derivatives (**2a**–**g**, **7**–**9**) were synthesized in good to high yields (66–95%); among them, several enamine-type derivatives showed modest activity against the HL-60 cell line. The crystal structure of compound **1a** was also studied by single-crystal XRD analysis. Furthermore, an intermediate (**3**) supporting the mechanism of the reaction was also identified and isolated.

## 4 Experimental Section

### 4.1 General Information

All starting materials were purchased from commercial sources and were used without further purification. The MW-assisted reactions were carried out in a 300 W CEM Discover focused MW reactor (CEM Microwave Technology Ltd., Buckingham, UK) equipped with a pressure controller using 10–20 W irradiation under isothermal conditions. The reactions under conventional heating were carried out in an oil bath.

High-performance liquid chromatography-mass spectrometry measurements were performed with an Agilent 1200 liquid chromatography system coupled with a 6130 quadrupole mass spectrometer equipped with an ESI ion source (Agilent Technologies, Palo Alto, CA, USA). Analysis was performed at 40 °C on a Gemini C18 column (150 mm × 4.6 mm, 3 μm; Phenomenex, Torrance, CA, USA) with a mobile phase flow rate of 0.6 mL/min. The composition of eluent A was 0.1% (NH_4_)(HCOO) in water; eluent B was 0.1% (NH_4_)(HCOO) and 8% water in acetonitrile, 0–3 min 5% B, 3–13 min gradient, 13–20 min 100% B. The injection volume was 2 μL. The chromatographic profile was registered at 254 nm. The MSD operating parameters were as follows: positive ionization mode, scan spectra from *m/z* 120 to 1000, drying gas temperature 300 °C, nitrogen flow rate 12 L/min, nebulizer pressure 60 psi, and capillary voltage 4000 V.

High-resolution mass spectrometric measurements were performed using a Sciex 5600+ Q-TOF mass spectrometer in positive electrospray mode.

The ^1^H, ^13^C, and ^31^P NMR spectra were taken in CDCl_3_ solution on a Bruker AV-300 spectrometer operating at 300, 75.5, and 121.5 MHz, respectively. The chemical shifts (δ) are reported in parts per million (ppm) and downfield relative to 85% H_3_PO_4_, as well as TMS, the coupling constants (*J*) are reported in Hz.

### 4.2 General Procedure for the Synthesis of 3-[(Amino)(diarylphosphoryl)methyl)]-6-methyl-4*H*-chromen-4-one Derivatives (**1a**–**d**, **4**–**6**)

A mixture of 1.0 mmol of 6-methyl-3-formylchromone (0.12 g), 1.0 mmol of secondary phosphine oxide (0.20 g of diphenylphosphine oxide, 0.23 g of bis(*p*-tolyl)phosphine oxide, 0.26 g of bis(3,5-dimethylphenyl)phosphine oxide or 0.30 g di(naphthalene-2-yl)phosphine oxide), and 1.0 mmol of amine (0.10 mL of butylamine, 0.09 mL of aniline, 0.12 g of *p*-anisidine or 0.13 g of 4-chloroaniline) was stirred in 1 mL of acetonitrile at 25 °C for 1–4 h. The products were purified by column chromatography using silica gel as the solid phase and dichloromethane-methanol (97:3) as the eluent. The following products were thus prepared.

#### 4.2.1 3-[(Butylamino)(diphenylphosphoryl)methyl]-6-methyl-4*H*-chromen-4-one (**1a**)

The title compound was isolated as a colorless solid (0.22 g, 49% yield). Mp: 187–188 °C; ^1^H NMR (300 MHz, CDCl_3_) δ 0.76 (t, *J*_HH_ = 7.4, 3H), 1.10–1.21 (m, 2H), 1.27–1.36 (m, 2H), 1.92 (brs, 1H), 2.39 (s, 3H), 2.43–2.50 (m, 1H), 2.56–2.63 (m, 1H), 5.15 (d, ^2^*J*_HP_ = 8.2, 1H), 7.24–7.34 (m, 4H), 7.40 (dd, *J*_HH_ = 8.6, *J*_HH_ = 2.4, 1H), 7.44–7.57 (m, 3H), 7.68–7.75 (m, 2H), 7.83 (d, *J*_HH_ = 2.4, 1H), 7.91–7.99 (m, 2H), 8.41 (d, *J*_HH_ = 2.7, 1H); ^13^C NMR (75 MHz, CDCl_3_) δ 13.8, 20.1, 20.9, 31.9, 47.8 (d, ^3^*J*_CP_ = 11.9), 50.3 (d, ^1^*J*_CP_ = 82.5), 118.0, 120.8 (d, ^2^*J*_CP_ = 0.9), 122.9, 125.2, 128.2 (d, ^3^*J*_CP_ = 11.6), 128.5 (d, ^3^*J*_CP_ = 11.8), 131.0 (d, ^2^*J*_CP_ = 9.1), 131.5 (d, ^2^*J*_CP_ = 9.1), 131.7 (d, *J*_CP_ = 3.1), 131.8 (d, *J*_CP_ = 2.6), 132.10 (d, ^1^*J*_CP_ = 103.4), 132.12 (d, ^1^*J*_CP_ = 97.7), 134.9, 135.2, 154.4, 156.0 (d, ^3^*J*_CP_ = 4.7), 176.4 (d, ^3^*J*_CP_ = 5.0); ^31^P (121.5 MHz, CDCl_3_) δ 32.2; [M + H]^+^_found_ = 446.1879, C_27_H_29_NO_3_P requires: 446.1877.

#### 4.2.2 3-[(Phenylamino)(diphenylphosphoryl)methyl]-6-methyl-4*H*-chromen-4-one (**1b**)

The title compound was isolated as a yellow solid (0.44 g, 94% yield). Mp: 239–240 °C; ^1^H NMR (300 MHz, CDCl_3_) δ 2.39 (s, 3H), 5.17 (brs, 1H), 5.95 (d, ^2^*J*_HP_ = 10.3, 1H), 6.64–6.72 (m, 3H), 7.10 (t, *J*_HH_ = 7.7, 2H), 7.22–7.29 (m, 3H), 7.31 (d, *J*_HH_ = 6.9, 1H), 7.39 (d, *J*_HH_ = 6.5, 1H), 7.43–7.51 (m, 2H), 7.51–7.57 (m, 1H), 7.69 (dd, *J*_HH_ = 11.7, *J*_HH_ = 7.4, 2H), 7.77 (d, *J*_HH_ = 2.2, 1H), 7.96 (dd, *J*_HH_ = 11.7, *J*_HH_ = 7.5, 2H), 8.30 (d, *J*_HH_ = 3.0, 1H); ^13^C NMR (75 MHz, CDCl_3_) δ 20.9, 45.3 (d, ^1^*J*_CP_ = 77.8), 113.5, 117.9, 118.5, 120.1 (d, ^2^*J*_CP_ = 1.1), 122.4, 124.9, 128.1 (d, ^3^*J*_CP_ = 12.3), 128.8 (d, ^3^*J*_CP_ = 11.9), 129.4, 129.7 (d, ^1^*J*_CP_ = 101.2), 131.3 (d, ^2^*J*_CP_ = 9.2), 131.4 (d, ^2^*J*_CP_ = 9.4), 132.1 (d, *J*_CP_ = 2.9), 132.3 (d, *J*_CP_ = 3.0), 134.9, 135.2, 145.5 (d, ^3^*J*_CP_ = 10.3), 145.9 (d, ^1^*J*_CP_ = 91.2), 154.3, 155.7 (d, ^3^*J*_CP_ = 4.5), 176.0 (d, ^3^*J*_CP_ = 3.3); ^31^P (121.5 MHz, CDCl_3_) δ 33.8; [M + Na]^+^_found_ = 488.1386, C_29_H_24_NO_3_NaP requires: 488.1386.

#### 4.2.3 3-[(4-Methoxyphenylamino)(diphenylphosphoryl)methyl]-6-methyl-4*H*-chromen-4-one (**1c**)

The title compound was isolated as a yellow solid (0.47 g, 94% yield). Mp: 224–225 °C; ^1^H NMR (300 MHz, CDCl_3_) δ 2.38 (s, 3H), 3.66 (s, 3H), 4.73 (brs, 1H), 5.89 (d, ^2^*J*_HP_ = 10.5, 1H), 6.66 (q, *J*_HH_ = 8.9, 4H), 7.20–7.33 (m, 4H), 7.38 (dd, *J*_HH_ = 8.6, *J*_HH_ = 2.3, 1H), 7.41–7.56 (m, 3H), 7.69 (dd, *J*_HH_ = 10.9, *J*_HH_ = 7.6, 2H), 7.77 (d, *J*_HH_ = 2.3, 1H), 7.94 (dd, *J*_HH_ = 7.4, *J*_HH_ = 4.2, 2H), 8.32 (d, *J*_HH_ = 3.1, 1H); ^13^C NMR (75 MHz, CDCl_3_) δ 20.9, 46.1 (d, ^1^*J*_CP_ = 78.2), 55.7, 114.8, 115.0, 118.0, 120.2 (d, ^2^*J*_CP_ = 1.1), 122.7, 125.0, 128.2 (d, ^3^*J*_CP_ = 12.0), 128.8 (d, ^3^*J*_CP_ = 11.9), 129.9 (d, ^1^*J*_CP_ = 101.0), 131.2 (d, ^1^*J*_CP_ = 98.3), 131.36 (d, ^2^*J*_CP_ = 9.4), 131.42 (d, ^2^*J*_CP_ = 9.5), 132.1 (d, *J*_CP_ = 2.9), 132.3 (d, *J*_CP_ = 3.1), 134.9, 135.2, 139.7 (d, ^3^*J*_CP_ = 11.2), 152.7, 154.4, 155.8 (d, ^3^*J*_CP_ = 4.5), 176.1 (d, ^3^*J*_CP_ = 3.5); ^31^P (121.5 MHz, CDCl_3_) δ 33.7; [M + Na]^+^_found_ = 518.1496, C_30_H_26_NO_4_NaP requires: 518.1491.

#### 4.2.4 3-[(4-Chlorophenylamino)(diphenylphosphoryl)methyl]-6-methyl-4*H*-chromen-4-one (**1d**)

The title compound was isolated as a yellow solid (0.46 g, 93% yield). Mp: 235–236 °C; ^1^H NMR (300 MHz, CDCl_3_) δ 2.39 (s, 3H), 5.34 (brs, 1H), 5.90 (d, ^2^*J*_HP_ = 10.4, 1H), 6.61 (d, *J*_HH_ = 8.7, 2H), 7.03 (d, *J*_HH_ = 8.7, 2H), 7.21–7.34 (m, 4H), 7.40 (dd, *J*_HH_ = 8.5, *J*_HH_ = 2.2, 1H), 7.43–7.58 (m, 3H), 7.68 (dd, *J*_HH_ = 11.8, *J*_HH_ = 6.8, 2H), 7.77 (d, *J*_HH_ = 2.2, 1H), 7.93 (dd, *J*_HH_ = 11.5, *J*_HH_ = 6.8, 2H), 8.33 (d, *J*_HH_ = 3.0, 1H); ^13^C NMR (75 MHz, CDCl_3_) δ 20.9, 45.5 (d, ^1^*J*_CP_ = 77.6), 114.8, 118.0, 119.9 (d, ^2^*J*_CP_ = 1.6), 122.6, 123.2, 125.0, 128.2 (d, ^3^*J*_CP_ = 12.1), 128.9 (d, ^3^*J*_CP_ = 11.8), 129.2, 130.8 (d, ^1^*J*_CP_ = 91.0), 130.9 (d, ^1^*J*_CP_ = 98.7), 131.2 (d, ^2^*J*_CP_ = 9.4), 131.4 (d, ^2^*J*_CP_ = 9.6), 132.3 (d, *J*_CP_ = 2.8), 132.4 (d, *J*_CP_ = 2.8), 135.1, 135.4, 144.4 (d, ^3^*J*_CP_ = 10.7), 154.4, 155.8 (d, ^3^*J*_CP_ = 4.7), 176.0 (d, ^3^*J*_CP_ = 3.5); ^31^P (121.5 MHz, CDCl_3_) δ 33.8; [M + Na]^+^_found_ = 522.0996, C_29_H_23_NO_3_NaPCl requires: 522.0995.

#### 4.2.5 3-[(Phenylamino)(di-*p*-tolylphosphoryl)methyl]-6-methyl-4*H*-chromen-4-one (**4**)

The title compound was isolated as a yellow solid (0.47 g, 95% yield). Mp: 245–246 °C; ^1^H NMR (300 MHz, CDCl_3_) δ 2.20 (s, 3H), 2.36 (s, 3H), 2.38 (s, 3H), 5.16 (brs, 1H), 5.88 (dd, ^2^*J*_HP_ = 10.0, *J*_HH_ = 6,2, 1H), 6.62–6.70 (m, 3H), 7.04 (dd, *J*_HH_ = 7.9, *J*_HH_ = 2.7, 2H), 7.08 (t, *J*_HH_ = 7.7, 2H), 7.21–7.28 (m, 3H), 7.37 (dd, *J*_HH_ = 8.5, *J*_HH_ = 2.3, 1H), 7.55 (dd, *J*_HH_ = 11.5, *J*_HH_ = 7.9, 2H), 7.78 (d, *J*_HH_ = 2.3, 1H), 7.81 (dd, *J*_HH_ = 11.3, *J*_HH_ = 8.0, 2H), 8.30 (d, *J*_HH_ = 3.0, 1H); ^13^C NMR (75 MHz, CDCl_3_) δ 20.9, 21.5, 21.7, 45.4 (d, ^1^*J*_CP_ = 77.5), 113.5, 118.0, 118.3, 120.4 (d, ^2^*J*_CP_ = 1.2), 122.8, 125.0, 126.7 (d, ^1^*J*_CP_ = 104.5), 128.0 (d, ^1^*J*_CP_ = 101.1), 128.9 (d, ^3^*J*_CP_ = 12.4), 129.4, 130.0 (d, ^3^*J*_CP_ = 12.2), 131.3 (d, ^2^*J*_CP_ = 9.8), 131.4 (d, ^2^*J*_CP_ = 9.9), 134.8, 135.1, 142.5 (d, *J*_CP_ = 2.7), 142.7 (d, *J*_CP_ = 2.8), 145.7 (d, ^3^*J*_CP_ = 10.6), 154.4, 155.7 (d, ^3^*J*_CP_ = 4.7), 176.1 (d, ^3^*J*_CP_ = 3.5); ^31^P (121.5 MHz, CDCl_3_) δ 34.2; [M + H]^+^_found_ = 494.1879, C_31_H_29_NO_3_P requires: 494.1886.

#### 4.2.6 3-{(Phenylamino)[bis(3,5-dimethylphenyl)phosphoryl]methyl}-6-methyl-4*H*-chromen-4-one (**5**)

The title compound was isolated as a yellow solid (0.48 g, 93% yield). Mp: 229–230 °C; ^1^H NMR (300 MHz, CDCl_3_) δ 2.15 (s, 6H), 2.34 (s, 6H), 2.43 (s, 3H), 5.14 (brs, 1H), 5.91 (dd, ^2^*J*_HP_ = 9.7, *J*_HH_ = 7.9, 1H), 6.63–6.75 (m, 3H), 6.92 (s, 1H), 7.04–7.19 (m, 3H), 7.22–7.35 (m, 3H), 7.40 (dd, *J*_HH_ = 8.6, *J*_HH_ = 2.3, 1H), 7.57 (d, *J*_HH_ = 11.7, 2H), 7.83 (d, *J*_HH_ = 2.2, 1H), 8.31 (d, *J*_HH_ = 3.0, 1H); ^13^C NMR (75 MHz, CDCl_3_) δ 21.0, 21.1, 21.4, 45.5 (d, ^1^*J*_CP_ = 76.2), 113.6, 117.9, 118.3, 120.5 (d, ^2^*J*_CP_ = 1.2), 122.9, 124.9, 128.77 (d, ^2^*J*_CP_ = 9.5), 128.84 (d, ^2^*J*_CP_ = 9.6), 129.3, 129.7 (d, ^1^*J*_CP_ = 99.7), 130.6 (d, ^1^*J*_CP_ = 98.5), 133.8 (d, *J*_CP_ = 3.0), 134.1 (d, *J*_CP_ = 3.1), 134.1, 135.1, 137.9 (d, ^3^*J*_CP_ = 12.6), 138.5 (d, ^3^*J*_CP_ = 12.3), 145.9 (d, ^3^*J*_CP_ = 10.3), 154.3, 155.6 (d, ^3^*J*_CP_ = 4.3), 176.1 (d, ^3^*J*_CP_ = 3.3); ^31^P (121.5 MHz, CDCl_3_) δ 34.7; [M + H]^+^_found_ = 522.2204, C_33_H_33_NO_3_P requires: 522.2192.

#### 4.2.7 3-{(Phenylamino)[di(naphthalen-2-yl)phosphoryl]methyl}-6-methyl-4*H*-chromen-4-one (**6**)

The title compound was isolated as a yellow solid (0.53 g, 94% yield). Mp: 223–224 °C; ^1^H NMR (300 MHz, CDCl_3_) δ 2.28 (s, 3H), 5.30 (t, *J*_HH_ = 8.8, 1H), 6.21 (dd, ^2^*J*_HP_ = 10.6, *J*_HH_ = 9.3, 1H), 6.67 (t, *J*_HH_ = 7.4, 1H), 6.73 (d, *J*_HH_ = 8.02, 2H), 7.10 (t, *J*_HH_ = 7.8, 2H), 7.17 (d, *J*_HH_ = 8.5, 1H), 7.29 (dd, *J*_HH_ = 8.6, *J*_HH_ = 2.3, 1H), 7.39–7.58 (m, 4H), 7.65 (d, *J*_HH_ = 2.3, 1H), 7.68–7.80 [7.73 (d, *J*_HH_ = 3.1) total int. 4H], 7.81–7.94 (m, 3H), 7.96–8.02 (m, 1H), 8.39 (d, *J*_HH_ = 13.3, 1H), 8.41 (d, *J*_HH_ = 3.0, 1H), 8.56 (d, *J*_HH_ = 13.4, 1H); ^13^C NMR (75 MHz, CDCl_3_) δ 21.0, 45.6 (d, ^1^*J*_CP_ = 78.1), 113.8, 118.0, 118.7, 120.4, 122.9, 125.1, 126.0 (d, *J*_CP_ = 1.5), 126.1 (d, *J*_CP_ = 2.6), 126.9, 127.1, 127.4 (d, ^1^*J*_CP_ = 101.6), 127.7, 127.9, 128.25 (d, *J*_CP_ = 12.1), 128.27, 128.5, 128.6 (d, ^1^*J*_CP_ = 98.9), 128.9 (d, *J*_CP_ = 11.9), 129.1, 129.2, 129.5, 132.4 (d, *J*_CP_ = 13.2), 132.8 (d, *J*_CP_ = 12.9), 133.6 (d, *J*_CP_ = 8.5), 133.9 (d, *J*_CP_ = 8.3), 134.9 (d, *J*_CP_ = 2.5), 135.0, 135.1 (d, *J*_CP_ = 2.4), 135.3, 145.8 (d, ^3^*J*_CP_ = 10.8), 154.5, 156.0 (d, ^3^*J*_CP_ = 4.5), 176.3 (d, ^3^*J*_CP_ = 3.4); ^31^P (121.5 MHz, CDCl_3_) δ 34.3; [M + H]^+^_found_ = 566.1891, C_37_H_29_NO_3_P requires: 566.1879.

### 4.3 General Procedure for the Synthesis of (*Z*)-3-[(Amino)methylene]-2-(diarylphosphoryl)-6-methylchroman-4-one Derivatives (**2a**–**g**, **7**–**9**)

A mixture of 1 mmol (0.12 g) of 6-methyl-3-formylchromone, 1.0 mmol of secondary phosphine oxides (0.20 g of diphenylphosphine oxide, 0.23 g of bis(*p*-tolyl)phosphine oxide, 0.26 g of bis(3,5-dimethylphenyl)phosphine oxide or 0.30 g bis(naphthalen-2-ylphoshine oxide), and 1.0 mmol of amines (0.10 mL of butylamine, 0.12 mL of cyclohexylamine, 0.11 mL of benzyl amine, 0.17 mL of benzhydrylamine, 0.06 mL of ethanolamine, or 0.08 mL of propanolamine) was stirred in 1 mL of acetonitrile at 60–80 °C for 1 h under a pressure-controlled 300 W in a CEM Discover MW reactor. The products were purified by column chromatography using silica gel as the solid phase and dichloromethane-methanol (97:3) as the eluent. The following products were thus prepared:

#### 4.3.1 (*Z*)-3-[(Butylamino)methylene]-2-(diphenylphosphoryl)-6-methylchroman-4-one (**2a**)

The title compound was isolated as a light-yellow solid (0.38 g, 86% yield). Mp: 192–193 °C; ^1^H NMR (300 MHz, CDCl_3_) δ 0.95 (t, *J*_HH_ = 7.3, 3H), 1.30–1.48 (m, 2H), 1.54–1.68 (m, 2H), 2.12 (s, 3H), 3.24–3.39 (m, 2H), 5.66 (d, ^2^*J*_HP_ = 4.6, 1H), 6.62 (d, *J*_HH_ = 8.3, 1H), 6.95 (dd, *J*_HH_ = 8.3, *J*_HH_ = 2.2, 1H), 7.02–7.16 [7.12 (d, *J*_HH_ = 2.4) total int. 4H], 7.22–7.33 (m, 1H), 7.34–7.46 (m, 2H), 7.50–7.65 (m, 3H), 8.02–8.15 (m, 2H), 10.45 (brdt, *J*_HH_ = 12.2, *J*_HH_ = 5.3, 1H); ^13^C NMR (75 MHz, CDCl_3_) δ 13.6, 19.6, 20.3, 32.9, 49.2, 78.6 (d, ^1^*J*_CP_ = 82,1), 91.6, 115.7, 122.5, 125.7, 127.7 (d, ^3^*J*_CP_ = 11.6), 128.5 (d, ^3^*J*_CP_ = 11.2), 129.7 (d, ^1^*J*_CP_ = 92.3), 130.56 (d, ^1^*J*_CP_ = 91.6), 130.64, 131.5 (d, *J*_CP_ = 3.0), 132.10 (d, *J*_CP_ = 3.4), 132.14 (d, ^2^*J*_CP_ = 8.6), 132.3 (d, ^2^*J*_CP_ = 9.7), 133.8, 153.4 (d, ^3^*J*_CP_ = 3.5), 154.5, 179.9 (d, ^3^*J*_CP_ = 1.2); ^31^P (121.5 MHz, CDCl_3_) δ 26.1; [M + H]^+^_found_ = 446.1877, C_27_H_29_NO_3_P requires: 446.1879.

#### 4.3.2 (*Z*)-3-[(Phenylamino)methylene]-2-(diphenylphosphoryl)-6-methylchroman-4-one (**2b**)

The title compound was isolated as a yellow solid (0.18 g, 38% yield). Mp: 243–244 °C; ^1^H NMR (300 MHz, CDCl_3_) δ 2.18 (s, 3H), 5.85 (d, ^2^*J*_HP_ = 5.3, 1H), 6.69 (d, ^2^*J*_HP_ = 8.3, 1H), 7.05 (dd, *J*_HH_ = 8.2, *J*_HH_ = 2.3, 1H), 7.08–7.20 (m, 6H), 7.22 (d, *J*_HH_ = 2.3, 1H), 7.30–7.41 (m, 3H), 7.43–7.54 [7.49 (dd, *J*_HH_ = 10.8, *J*_HH_ = 7.5), total int. 3H], 7.60 (dd, *J*_HH_ = 7.6, *J*_HH_ = 2.7, 2H), 8.08–8.17 (m, 2H), 12.22 (d, *J*_HH_ = 12.3 Hz, 1H); ^13^C NMR (75 MHz, CDCl_3_) δ 20.4, 78.5 (d, ^1^*J*_CP_ = 81.1), 95.6, 113.6, 116.2, 116.7, 116.8, 117.9, 118.5, 122.1, 124.2, 125.8, 128.0 (d, ^3^*J*_CP_ = 11.6), 128.7 (d, ^3^*J*_CP_ = 11.4), 129.4, 129.7, 129.9, 130.6, 131.1, 131.3, 131.8 (d, *J*_CP_ = 2.8), 132.2 (d, ^2^*J*_CP_ = 8.9), 132.4 (d, *J*_CP_ = 2.9), 132.5 (d, ^2^*J*_CP_ = 9.8), 134.9, 139.7, 144.0 (d, ^3^*J*_CP_ = 4.2), 155.1, 181.3; ^31^P (121.5 MHz, CDCl_3_) δ 26.4; [M + Na]^+^_found_ = 488.1388, C_29_H_24_NO_3_NaP requires: 488.1386.

#### 4.3.3 (*Z*)-3-[(Cyclohexylamino)methylene]-2-(diphenylphosphoryl)-6-methylchroman-4-one (**2c**)

The title compound was isolated as a light-yellow solid (0.44 g, 93% yield). Mp: 198–199 °C; ^1^H NMR (300 MHz, CDCl_3_) δ 1.18–1.49 (m, 5H), 1.56–1.64 (m, 1H), 1.74–1.84 (m, 2H), 1.90–2.06 (m, 2H), 2.12 (s, 3H), 3.17–3.26 (m, 1H), (d, ^2^*J*_HP_ = 4.1, 1H), 6.62 (d, *J*_HH_ = 8.3, 1H), 6.95 (dd, *J*_HH_ = 8.2, *J*_HH_ = 2.5, 1H), 7.07–7.19 [7.10 (d, *J*_HH_ = 3.0) total int. 4H], 7.25–7.32 (m, 1H), 7.40 (dd, *J*_HH_ = 11.1, *J*_HH_ = 7.7, 2H) 7.52–7.63 (m, 3H), 8.09 (t, *J*_HH_ = 9.4, 2H), 10.54 (brdt, *J*_HH_ = 13.4, *J*_HH_ = 7.5, 1H); ^13^C NMR (75 MHz, CDCl_3_) δ 20.2, 24.3, 25.1, 33.80, 33.82, 57.4, 78.7 (d, ^1^*J*_CP_ = 81.9), 91.4, 115.7, 122.5, 125.4, 127.7 (d, ^3^*J*_CP_ = 11.5), 128.5 (d, ^3^*J*_CP_ = 11.2), 129.7 (d, ^1^*J*_CP_ = 92.3), 130.56 (d, ^1^*J*_CP_ = 91.3), 130.59, 131.4 (d, *J*_CP_ = 2.8), 132.06 (d, *J*_CP_ = 2.8), 132.10 (d, ^2^*J*_CP_ = 8.6), 132.3 (d, ^2^*J*_CP_ = 9.8), 133.8, 151.5 (d, ^3^*J*_CP_ = 3.5), 154.5, 179.7 (d, ^3^*J*_CP_ = 0.9); ^31^P (121.5 MHz, CDCl_3_) δ 25.9; [M + H]^+^_found_ = 472.2030, C_29_H_31_NO_3_P requires: 472.2036.

#### 4.3.4 (*Z*)-3-[(Benzylamino)methylene]-2-(diphenylphosphoryl)-6-methylchroman-4-one (**2d**)

The title compound was isolated as a light-yellow solid (0.44 g, 91% yield). Mp: 196–197 °C; ^1^H NMR (300 MHz, CDCl_3_) δ 2.12 (s, 3H), 4.49 (qd, *J*_HH_ = 15.1, *J*_HH_ = 5.9, 2H), 5.68 (d, ^2^*J*_HP_ = 4.5, 1H), 6.63 (d, *J*_HH_ = 8.4, 1H), 6.97 (d, *J*_HH_ = 8.4, 1H), 7.06–7.14 [7.09 (d, *J*_HH_ = 3.1) total int. 3H], 7.18 (d, *J*_HH_ = 12.8, 1H), 7.25–7.33 (m, 4H), 7.33–7.46 (m, 4H), 7.52–7.63 (m, 3H), 8.09 (t, *J*_HH_ = 9.0, 2H), 10.66 (brdt, *J*_HH_ = 11.7, *J*_HH_ = 6.1, 1H); ^13^C NMR (75 MHz, CDCl_3_) δ 20.3, 53.1, 78.6 (d, ^1^*J*_CP_ = 81.8), 92.5, 115.8, 122.4, 125.6, 127.3, 127.78 (d, ^3^*J*_CP_ = 11.7), 127.84, 128.6 (d, ^3^*J*_CP_ = 11.2), 128.9, 129.7 (d, ^1^*J*_CP_ = 92.5), 130.5 (d, ^1^*J*_CP_ = 91.5), 130.7, 131.5 (d, *J*_CP_ = 2.9), 132.10 (d, ^2^*J*_CP_ = 8.7), 132.15 (d, *J*_CP_ = 2.3), 132.4 (d, ^2^*J*_CP_ = 9.8), 134.1, 137.3, 153.0 (d, ^3^*J*_CP_ = 3.7), 154.7, 180.3 (d, ^3^*J*_CP_ = 1.4); ^31^P (121.5 MHz, CDCl_3_) δ 26.2; [M + H]^+^_found_ = 480.1738, C_30_H_27_NO_3_P requires: 480.1723.

#### 4.3.5 (*Z*)-3-[(Benzhydrylamino)methylene]-2-(diphenylphosphoryl)-6-methylchroman-4-one (**2e**)

The title compound was isolated as a yellow solid (0.49 g, 89% yield). Mp: 182–183 °C; ^1^H NMR (300 MHz, CDCl_3_) δ 2.14 (s, 3H), 5.60–5.66 [5.64 (d, ^2^*J*_HP_ = 5.1) total int. 2H], 6.62 (d, *J*_HH_ = 8.3, 1H), 6.98 (dd, *J*_HH_ = 8.1, *J*_HH_ = 2.3, 1H), 7.08–7.16 [7.11 (d, *J*_HH_ = 3.9, C_5_H) total int. 3H], 7.18 (s), 7.22–7.40 (m, 11H), 7.40–7.47 (m, 2H), 7.47–7.54 (m, 2H), 7.55–7.62 (m, 1H), 8.03 (dd, *J*_HH_ = 10.6, *J*_HH_ = 7.4, 2H), 11.11 (brdt, *J*_HH_ = 12.6, *J*_HH_ = 7.0, 1H); ^13^C NMR (75 MHz, CDCl_3_) δ 20.4, 66.5, 78.6 (d, ^1^*J*_CP_ = 82.1), 93.3, 116.0, 122.5, 125.4, 127.4, 127.5, 127.89, 127.92 (d, ^3^*J*_CP_ = 11.0), 128.0, 128.6 (d, ^3^*J*_CP_ = 11.3), 128.98, 128.99, 129.8 (d, ^1^*J*_CP_ = 92.8), 130.5 (d, ^1^*J*_CP_ = 91.8), 130.9, 131.6 (d, *J*_CP_ = 2.7), 132.17 (d, ^2^*J*_CP_ = 8.5), 132.19 (d, *J*_CP_ = 2.7), 132.4 (d, ^2^*J*_CP_ = 9.8), 134.4, 140.7, 141.1, 151.7 (d, ^3^*J*_CP_ = 3.7), 154.9, 180.8 (d, ^3^*J*_CP_ = 1.5); ^31^P (121.5 MHz, CDCl_3_) δ 26.4; [M + H]^+^_found_ = 556.2037, C_36_H_31_NO_3_P requires: 556.2036.

#### 4.3.6 (*Z*)-3-[(Propanolamino)methylene]-2-(diphenylphosphoryl)-6-methylchroman-4-one (**2f**)

The title compound was isolated as a yellow solid (0.33 g, 73% yield). Mp: 176–177 °C; ^1^H NMR (300 MHz, CDCl_3_) δ 1.72–1.82 (m, 1H), 1.87–1.96 (m, 1H), 2.11 (s, 3H), 3.51–3.59 (m, 3H), 3.77–3.84 (m, 1H), 3.84–3.92 (m, 1H), 5.71 (d, ^2^*J*_HP_ = 4.2, 1H), 6.60 (d, *J*_HH_ = 8.3, 1H), 6.93 (dd, *J*_HH_ = 8.3, *J*_HH_ = 2.4, 1H), 7.02–7.10 [7.08 (d, *J*_HH_ = 2.4) total int. 2H], 7.15–7.39 (m, 5H), 7.53–7.66 (m, 3H), 8.11 (dd, *J*_HH_ = 10.9, *J*_HH_ = 7.2, 2H), 10.22 (brdt, *J*_HH_ = 13.5, *J*_HH_ = 6.7, 1H); ^13^C NMR (75 MHz, CDCl_3_) δ 20.5, 32.3, 46.3, 57.8, 78.8 (d, ^1^*J*_CP_ = 81.7), 90.8, 116.0, 122.4, 125.7, 128.0 (d, ^3^*J*_CP_ = 11.6), 128.9 (d, ^3^*J*_CP_ = 11.3), 129.3 (d, ^1^*J*_CP_ = 92.1), 130.5 (d, ^1^*J*_CP_ = 92.2), 130.8, 131.8 (d, *J*_CP_ = 2.8), 132.3 (d, ^2^*J*_CP_ = 8.8), 132.57 (d, *J*_CP_ = 3.5), 132.62 (d, ^2^*J*_CP_ = 10.4), 134.1, 154.7, 154.8 (d, ^3^*J*_CP_ = 3.1), 179.9 (d, ^3^*J*_CP_ = 0.9); ^31^P (121.5 MHz, CDCl_3_) δ 25.9; [M + H]^+^_found_ = 448.1668, C_26_H_27_NO_4_P requires: 448.1672.

#### 4.3.7 (*Z*)-3-[(Ethanolamino)methylene]-2-(diphenylphosphoryl)-6-methylchroman-4-one (**2g**)

The title compound was isolated as a yellow solid (0.29 g, 66% yield). Mp: 181–182 °C; ^1^H NMR (300 MHz, CDCl_3_) δ 2.12 (s, 3H), 3.40–3.51 (m, 2H), 3.79 (t, *J*_HH_ = 5.0, 3H), 5.68 (d, ^2^*J*_HP_ = 4.2, 1H), 6.60 (d, *J*_HH_ = 8.2, 1H), 6.94 (dd, *J*_HH_ = 8.3, *J*_HH_ = 2.2, 1H), 7.03–7.13 [7.10 (d, *J*_HH_ = 2.3) total int. 2H], 7.14–7.41 (m, 5H), 7.49–7.68 (m, 3H), 8.04–8.17 (m, 2H), 10.41 (brdt, *J*_HH_ = 13.4, *J*_HH_ = 6.0, 1H); ^13^C NMR (75 MHz, CDCl_3_) δ 20.3, 51.5, 62.4, 78.8 (d, ^1^*J*_CP_ = 81.7), 91.7, 115.9, 122.4, 125.6, 127.9 (d, ^3^*J*_CP_ = 11.5), 128.7 (d, ^3^*J*_CP_ = 11.2), 129.4 (d, ^1^*J*_CP_ = 92.6), 130.6 (d, ^1^*J*_CP_ = 91.8), 130.7, 131.6 (d, *J*_CP_ = 3.0), 132.1 (d, ^2^*J*_CP_ = 8.9), 132.4 (d, *J*_CP_ = 2.7), 132.5 (d, ^2^*J*_CP_ = 10.2), 134.1, 153.1 (d, ^3^*J*_CP_ = 3.4), 154.6, 180.0 (d, ^3^*J*_CP_ = 0.9); ^31^P (121.5 MHz, CDCl_3_) δ 26.3; [M + H]^+^_found_ = 434.1518, C_25_H_25_NO_4_P requires: 434.1515.

#### 4.3.8 (*Z*)-3-[(Butylamino)methylene]-2-(di-p-tolylphosphoryl)-6-methylchroman-4-one (**7a**)

The title compound was isolated as a light-yellow solid (0.41 g, 87% yield). Mp: 186–187 °C; ^1^H NMR (300 MHz, CDCl_3_) δ 0.95 (t, *J*_HH_ = 7.3, 3H), 1.31–1.49 (m, 2H), 1.54–1.67 (m, 2H), 2.15 (s, 3H), 2.23 (s, 3H), 2.44 (s, 3H), 3.24–3.39 (m, 2H), 5.60 (d, ^2^*J*_HP_ = 4.8, 1H), 6.59 (d, *J*_HH_ = 8.3, 1H), 6.89 (dd, *J*_HH_ = 8.2, *J*_HH_ = 2.8, 2H), 6.95 (dd, *J*_HH_ = 8.4, *J*_HH_ = 2.4, 1H), 7.01–7.17 [7.11 (d, *J*_HH_ = 2.3) total int. 2H], 7.23–7.30 (m, 2H), 7.35 (dd, *J*_HH_ = 8.1, *J*_HH_ = 2.7, 2H), 7.96 (dd, *J*_HH_ = 10.4, *J*_HH_ = 8.1, 2H), 10.46 (brdt, *J*_HH_ = 12.2, *J*_HH_ = 5.3, 1H); ^13^C NMR (75 MHz, CDCl_3_) δ 13.6, 19.7, 20.3, 21.4, 21.7, 32.9, 49.2, 78.6 (d, ^1^*J*_CP_ = 82.5), 91.8, 115.7, 122.5, 125.5, 126.5 (d, ^1^*J*_CP_ = 95.2), 127.3 (d, ^1^*J*_CP_ = 94.3), 128.4 (d, ^3^*J*_CP_ = 11.9), 129.3 (d, ^3^*J*_CP_ = 11.5), 130.5, 132.2 (d, ^2^*J*_CP_ = 9.1), 132.4 (d, ^2^*J*_CP_ = 10.2), 133.7, 142.2 (d, *J*_CP_ = 2.8), 142.5 (d, *J*_CP_ = 2.4), 153.4 (d, ^3^*J*_CP_ = 3.9), 154.6, 180.0 (d, ^3^*J*_CP_ = 1.1); ^31^P (121.5 MHz, CDCl_3_) δ 26.5; [M + H]^+^_found_ = 474.2189, C_29_H_33_NO_3_P requires:474.2192.

#### 4.3.9 (*Z*)-3-[(Cyclohexylamino)methylene]-2-(di-p-tolylphosphoryl)-6-methylchroman-4-one (**7b**)

The title compound was isolated as a light-yellow solid (0.45 g, 91% yield). Mp: 205–206 °C; ^1^H NMR (300 MHz, CDCl_3_) δ 1.22–1.52 (m, 5H), 1.59–1.67 (m, 1H), 1.76–1.86 (m, 2H), 1.92–2.07 (m, 2H), 2.17 (s, 3H), 2.25 (s, 3H), 2.46 (s, 3H), 3.19–3.29 (m, 1H), 5.62 (d, ^2^*J*_HP_ = 4.4, 1H), 6.62 (d, *J*_HH_ = 8.3, 1H), 6.92 (d, *J*_HH_ = 5.1, 1H), 6.97 (d, *J*_HH_ = 7.6, 2H), 7.12–7.22 [7.14 (d, *J*_HH_ = 2.8) total int. 2H], 7.28 (dd, *J*_HH_ = 7.5, *J*_HH_ = 3.9, 2H), 7.37 (d, *J*_HH_ = 4.7, 2H), 7.98 (dd, *J*_HH_ = 10.4, *J*_HH_ = 7.5, 2H), 10.56 (brdt, *J*_HH_ = 13.3, *J*_HH_ = 7.3, 1H); ^13^C NMR (75 MHz, CDCl_3_) δ 20.4, 21.5, 21.7, 24.4, 25.3, 33.92, 33.93, 57.4, 78.8 (d, ^1^*J*_CP_ = 82.3), 91.8, 115.8, 122.6, 125.5, 126.6 (d, ^1^*J*_CP_ = 94.9), 127.4 (d, ^1^*J*_CP_ = 94.0), 128.5 (d, ^3^*J*_CP_ = 11.8), 129.3 (d, ^3^*J*_CP_ = 11.5), 130.5, 132.3 (d, ^2^*J*_CP_ = 9.0), 132.4 (d, ^2^*J*_CP_ = 10.1), 133.7, 142.2 (d, *J*_CP_ = 3.0), 142.6 (d, *J*_CP_ = 2.8), 151.6 (d, ^3^*J*_CP_ = 3.2), 154.5, 179.9 (d, ^3^*J*_CP_ = 1.1); ^31^P (121.5 MHz, CDCl_3_) δ 26.2; [M + H]^+^_found_ = 500.2359, C_31_H_35_NO_3_P requires: 500.2349.

#### 4.3.10 (*Z*)-3-[(Benzylamino)methylene]-2-(di-p-tolylphosphoryl)-6-methylchroman-4-one (**7c**)

The title compound was isolated as a light-yellow solid (0.45 g, 88% yield). Mp: 198–199 °C; ^1^H NMR (300 MHz, CDCl_3_) δ 2.13 (s, 3H), 2.22 (s, 3H), 2.43 (s, 3H), 4.48 (qd, *J*_HH_ = 15.2, *J*_HH_ = 6.1, 2H), 5.62 (d, ^2^*J*_HP_ = 4.8, 1H), 6.59 (d, *J*_HH_ = 8.3, 1H), 6.87 (d, *J*_HH_ = 5.3, 2H), 6.95 (dd, *J*_HH_ = 8.3, *J*_HH_ = 8.3, 1H), 7.11 (d, *J*_HH_ = 2.4, 1H), 7.18 (d, *J*_HH_ = 10.9, 1H), 7.23–7.39 (m, 9H), 7.95 (dd, *J*_HH_ = 10.5, *J*_HH_ = 7.8, 2H), 10.64 (brdt, *J*_HH_ = 12.7, *J*_HH_ = 6.1, 1H); ^13^C NMR (75 MHz, CDCl_3_) δ 20.3, 21.5, 21.7, 53.2, 78.6 (d, ^1^*J*_CP_ = 82.4), 92.8, 115.9, 122.5, 125.6, 127.30 (d, ^1^*J*_CP_ = 94.8), 127.34, 127.5 (d, ^1^*J*_CP_ = 95.2), 127.9, 128.5 (d, ^3^*J*_CP_ = 11.9), 129.0, 129.3 (d, ^3^*J*_CP_ = 11.6), 130.6, 132.2 (d, ^2^*J*_CP_ = 9.0), 132.4 (d, ^2^*J*_CP_ = 10.1), 134.0, 137.4, 142.3 (d, *J*_CP_ = 2.9), 142.6 (d, *J*_CP_ = 2.8), 153.0 (d, ^3^*J*_CP_ = 3.7), 154.8, 180.5 (d, ^3^*J*_CP_ = 1.2); ^31^P (121.5 MHz, CDCl_3_) δ 26.5; [M + H]^+^_found_ = 508.2044, C_32_H_31_NO_3_P requires: 508.2036.

#### 4.3.11 (*Z*)-2-[Bis(3,5-dimethylphenyl)phosphoryl]-3-[(butylamino)methylene]-6-methyl-chroman-4-one (**8a**)

The title compound was isolated as a light-yellow solid (0.45 g, 89% yield). Mp: 145–146 °C; ^1^H NMR (300 MHz, CDCl_3_) δ 0.96 (t, *J*_HH_ = 7.3, 3H), 1.35–1.50 (m, 2H), 1.55–1.68 (m, 2H), 2.08 (s, 6H), 2.15 (s, 3H), 2.40 (s, 6H), 3.25–3.40 (m, 2H), 5.58 (d, ^2^*J*_HP_ = 4.9, 1H), 6.62 (d, *J*_HH_ = 8.3, 1H), 6.87 (s, 1H), 6.92–7.02 (m, 3H, ArH), 7.06 (dd, *J*_HH_ = 12.8, *J*_HH_ = 1.9, 1H), 7.12 (d, *J*_HH_ = 2.3, 1H), 7.22 (s, 1H), 7.69 (d, *J*_HH_ = 10.8, 2H), 10.43 (brdt, *J*_HH_ = 11.5, *J*_HH_ = 5.9, 1H); ^13^C NMR (75 MHz, CDCl_3_) δ 13.6, 19.6, 20.3, 20.9, 21.4, 32.9, 49.2, 78.8 (d, ^1^*J*_CP_ = 81.2), 91.8, 115.7, 122.5, 125.3, 129.3 (d, ^1^*J*_CP_ = 91.5), 129.6 (d, ^2^*J*_CP_ = 8.7), 130.2 (d, ^2^*J*_CP_ = 9.8), 130.3, 130.4 (d, ^1^*J*_CP_ = 90.6), 133.1 (d, *J*_CP_ = 3.0), 133.5, 133.7 (d, *J*_CP_ = 2.9), 137.3 (d, ^3^*J*_CP_ = 12.1), 138.1 (d, ^3^*J*_CP_ = 11.8), 153.2 (d, ^3^*J*_CP_ = 3.4), 154.7, 179.9 (d, ^3^*J*_CP_ = 0.9); ^31^P (121.5 MHz, CDCl_3_) δ 26,5; [M + H]^+^_found_ = 502.2503, C_31_H_37_NO_3_P requires: 502.2505.

#### 4.3.12 (*Z*)-2-[Bis(3,5-dimethylphenyl)phosphoryl]-3-[(cyclohexylamino)methylene]-6-methylchroman-4-one (**8b**)

The title compound was isolated as a light-yellow solid (0.47 g, 90% yield). Mp: 155–156 °C; ^1^H NMR (300 MHz, CDCl_3_ δ 1.18–1.50 (m, 5H), 1.54–1.63 (m, 1H), 1.71–1.84 (m, 2H), 1.88–1.95 (m, 1H), 1.95–2.02 (m, 1H), 2.07 (s, 6H), 2.23 (s, 3H), 2.39 (s, 6H), 3.16–3.26 (m, 1H), 5.57 (d, ^2^*J*_HP_ = 4.5, 1H), 6.60 (d, *J*_HH_ = 8.2, 1H), 6.86 (s, 1H), 6.92–6.99 [6.95 (d, *J*_HH_ = 11.6) total int. 3H], 7.09–7.15 [7.11 (d, *J*_HH_ = 2.8) total int. 2H], 7.20 (s, 1H), 7.67 (d, *J*_HH_ = 10.9, 2H), 10.49 (brdt, *J*_HH_ = 12.8, *J*_HH_ = 7.4, 1H); ^13^C NMR (75 MHz, CDCl_3_) δ 20.3, 20.9, 21.4, 24.3, 25.2, 33.87, 33.90, 57.3, 78.9 (d, ^1^*J*_CP_ = 81.0), 91.4, 115.8, 122.6, 125.4, 129.4 (d, ^1^*J*_CP_ = 91.5), 129.7 (d, ^2^*J*_CP_ = 8.6), 130.2 (d, ^2^*J*_CP_ = 9.7), 130.4, 130.5 (d, ^1^*J*_CP_ = 90.4), 133.2 (d, *J*_CP_ = 3.1), 133.5, 133.8 (d, *J*_CP_ = 2.8), 137.4 (d, ^3^*J*_CP_ = 12.2), 138.2 (d, ^3^*J*_CP_ = 11.8), 151.3 (d, ^3^*J*_CP_ = 3.3), 154.5, 179.8 (d, ^3^*J*_CP_ = 0.9); ^31^P (121.5 MHz, CDCl_3_) δ 26.6; [M + H]^+^_found_ = 528.2667, C_33_H_39_NO_3_P requires: 528.2662.

#### 4.3.13 (*Z*)-2-[Bis(3,5-dimethylphenyl)phosphoryl]-3-[(benzylamino)methylene]-6-methylchroman-4-one (**8c**)

The title compound was isolated as a light-yellow solid (0.47 g, 87% yield). Mp: 182–182 °C; ^1^H NMR (300 MHz, CDCl_3_) δ 2.05 (s, 6H), 2.13 (s, 3H), 2.39 (s, 6H), 4.48 (qd, *J*_HH_ = 15.3, *J*_HH_ = 6.1, 2H), 5.61 (d, ^2^*J*_HP_ = 5.0, 1H), 6.62 (d, *J*_HH_ = 8.3, 1H), 6.86 (s, 1H), 6.93–7.01 [6.97 (d, *J*_HH_ = 10.6) total int. 3H], 7.10 (d, *J*_HH_ = 2.4, 1H), 7.16 (dd, *J*_HH_ = 12.8, *J*_HH_ = 2.0, 1H), 7.21 (s, 1H), 7.28–7.39 (m, 6H), 7.68 (d, *J*_HH_ = 11.0, 2H), 10.61 (brdt, *J*_HH_ = 12.7, *J*_HH_ = 6.1, 1H); ^13^C NMR (75 MHz, CDCl_3_) δ 20.4, 21.0, 21.5, 53.2, 78.8 (d, ^1^*J*_CP_ = 81.0), 92.7, 115.9, 122.5, 125.6, 127.3, 127.9, 128.9, 129.3 (d, ^1^*J*_CP_ = 91.5), 129.7 (d, ^2^*J*_CP_ = 8.7), 130.3 (d, ^2^*J*_CP_ = 9.9), 130.38 (d, ^1^*J*_CP_ = 90.2), 130.44, 133.3 (d, *J*_CP_ = 3.1), 133.85 (d, *J*_CP_ = 2.5), 133.86, 137.5 (d, ^3^*J*_CP_ = 12.1), 137.6, 138.2 (d, ^3^*J*_CP_ = 11.8), 152.9 (d, ^3^*J*_CP_ = 3.7), 154.9, 180.4 (d, ^3^*J*_CP_ = 1.0); ^31^P (121.5 MHz, CDCl_3_) δ 26.7; [M + H]^+^_found_ = 536.2352, C_32_H_35_NO_3_P requires: 536.2349.

#### 4.3.14 (*Z*)-3-[(Butylamino)methylene]-2-[di(naphthalen-2-yl)phosphoryl]-6-methylchroman-4-one (**9a**)

The title compound was isolated as a light-yellow solid (0.49 g, 90% yield). Mp: 192–193 °C; ^1^H NMR (300 MHz, CDCl_3_) δ 0.93 (t, *J*_HH_ = 7.2, 3H), 1.31–1.46 (m, 2H), 1.82 (s, 3H), 1.51–1.64 (m, 2H), 3.23–3.37 (m, 2H), 5.80 (d, ^2^*J*_HP_ = 4.3, 1H), 6.63 (d, *J*_HH_ = 9.0, 1H), 6.80 (d, *J*_HH_ = 3.3, 1H), 6.81 (d, *J*_HH_ = 6.8, 1H), 7.13 (d, *J*_HH_ = 12.9, 1H), 7.36–7.52 (m, 3H), 7.52–7.67 (m, 4H), 7.72 (d, *J*_HH_ = 7.7, 1H), 7.88–8.16 (m, 5H), 8.76 (d, *J*_HH_ = 12.5, 1H), 10.47 (brdt, *J*_HH_ = 13.5, *J*_HH_ = 5.5, 1H); ^13^C NMR (75 MHz, CDCl_3_) δ 13.6, 19.6, 20.0, 32.9, 49.2, 79.0 (d, ^1^*J*_CP_ = 82.2), 91.9, 115.6, 122.6, 125.3, 126.3, 126.89 (d, *J*_CP_ = 3.8), 126.91, 127.0 (d, *J*_CP_ = 3.4), 127.3 (d, ^1^*J*_CP_ = 92.6), 127.4 (d, *J*_CP_ = 11.2), 127.5, 127.85, 127.87 (d, ^1^*J*_CP_ = 91.6), 128.0, 128.29, 128.30 (d, *J*_CP_ = 11.0), 128.7, 129.1, 130.8, 131.9 (d, *J*_CP_ = 12.9), 132.6 (d, *J*_CP_ = 12.4), 133.7, 134.3 (d, *J*_CP_ = 8.4), 134.6 (d, *J*_CP_ = 2.3), 134.8 (d, *J*_CP_ = 10.2), 134.9 (d, *J*_CP_ = 2.4), 153.3 (d, ^3^*J*_CP_ = 3.4), 154.4, 179.9 (d, ^3^*J*_CP_ = 1.2); ^31^P (121.5 MHz, CDCl_3_) δ 26.6; [M + H]^+^_found_ = 546.2211, C_35_H_33_NO_3_P requires: 546.2192.

#### 4.3.15 (*Z*)-3-[(Cyclohexylamino)methylene]-2-[di(naphthalen-2-yl)phosphoryl]-6-methylchroman-4-one (**9b**)

The title compound was isolated as a light-yellow solid (0.51 g, 90% yield). Mp: 171–172 °C; ^1^H NMR (300 MHz, CDCl_3_) δ 1.16–1.49 (m, 5H), 1.53–1.68 (m, 1H), 1.68–2.07 [1.84 (s) total int. 7H], 3.12–3.27 (m, 1H), 5.82 (d, ^2^*J*_HP_ = 3.8, 1H), 6.65 (d, *J*_HH_ = 7.9, 1H), 6.82 (d, *J*_HH_ = 2.5, 1H), 6.83 (d, *J*_HH_ = 8.3, 1H), 7.17 (d, *J*_HH_ = 11.2, 1H), 7.39–7.56 (m, 3H), 7.56–7.71 (m, 4H), 7.75 (d, *J*_HH_ = 7.9, 1H), 7.88–8.18 (m, 5H), 8.78 (d, *J*_HH_ = 12.5, 1H), 10.55 (brdt, *J*_HH_ = 13.1, *J*_HH_ = 7.5, 1H); ^13^C NMR (75 MHz, CDCl_3_) δ 20.1, 24.4, 25.2, 33.88, 33.89, 57.4, 79.2 (d, ^1^*J*_CP_ = 81.8), 91.8, 115.7, 122.7, 125.4, 126.4, 126.90 (d, *J*_CP_ = 3.4), 126.94, 127.1 (d, *J*_CP_ = 3.4), 127.4 (d, ^1^*J*_CP_ = 92.4), 127.5 (d, *J*_CP_ = 11.2), 127.6, 127.9, 127.95 (d, ^1^*J*_CP_ = 91.5), 128.02, 128.36, 128.38 (d, *J*_CP_ = 11.1), 128.8, 129.2, 130.9, 132.0 (d, *J*_CP_ = 12.7), 132.7 (d, *J*_CP_ = 12.2), 133.8, 134.4 (d, *J*_CP_ = 8.3), 134.7 (d, *J*_CP_ = 2.2), 134.8 (d, *J*_CP_ = 9.8), 134.9 (d, *J*_CP_ = 2.6), 151.4 (d, ^3^*J*_CP_ = 3.4), 154.5, 179.8 (d, ^3^*J*_CP_ = 0.9); ^31^P (121.5 MHz, CDCl_3_) δ 26.6; [M + H]^+^_found_ = 572.2358, C_37_H_35_NO_3_P requires: 572.2349.

#### 4.3.16 (*Z*)-3-[(Benzylamino)methylene]-2-[di(naphthalen-2-yl)phosphoryl]-6-methylchroman-4-one (**9c**)

The title compound was isolated as a light-yellow solid (0.52 g, 90% yield). Mp: 200–201 °C; ^1^H NMR (300 MHz, CDCl_3_) δ 1.81 (s, 3H), 4.52 (qd, *J*_HH_ = 15.1, *J*_HH_ = 6.4, 2H), 5.82 (d, ^2^*J*_HP_ = 4.5, 1H), 6.64 (d, *J*_HH_ = 8.3, 1H), 6.77 (s, 1H), 6.83 (d, *J*_HH_ = 9.8, 1H), 7.20–7.39 (m, 6H), 7.40–7.54 (m, 3H), 7.54–7.68 (m, 3H), 7.73 (d, *J*_HH_ = 8.0, 1H), 7.85–8.16 (m, 6H), 8.75 (d, *J*_HH_ = 12.7, 1H), 10.65 (brd, *J*_HH_ = 12.4, 1H); ^13^C NMR (75 MHz, CDCl_3_) δ 20.0, 53.2, 78.9 (d, ^1^*J*_CP_ = 82.1), 92.6, 115.8, 122.5, 125.4, 126.5, 126.9 (d, *J*_CP_ = 4.0), 127.00, 127.04 (d, *J*_CP_ = 3.7), 127.4, 127.5, 127.6 (d, *J*_CP_ = 11.4), 127.7 (d, ^1^*J*_CP_ = 88.5), 127.93, 127.95, 128.00 (d, ^1^*J*_CP_ = 91.6), 128.1, 128.48, 128.50 (d, *J*_CP_ = 11.2), 128.8, 128.9, 129.2, 131.0, 132.0 (d, *J*_CP_ = 12.7), 132.7 (d, *J*_CP_ = 12.3), 134.1, 134.3 (d, *J*_CP_ = 8.4), 134.7 (d, *J*_CP_ = 2.2), 134.9 (d, *J*_CP_ = 8.2), 135.0 (d, *J*_CP_ = 1.5), 137.3, 153.3 (d, ^3^*J*_CP_ = 3.8), 154.6, 180.4 (d, ^3^*J*_CP_ = 1.0); ^31^P (121.5 MHz, CDCl_3_) δ 29.4; [M + H]^+^_found_ = 580.2044, C_37_H_35_NO_3_P requires: 580.2036.

### 4.4 General Procedure for the Synthesis of 3-[(Diphenylphosphoryl)(hydroxy)methyl]-6-methyl-4*H*-chromen-4-one (**3**)

A mixture of 1.0 mmol (0.12 g) of 6-methyl-3-formylchromone and 1.0 mmol (0.20 g) of diphenylphosphine oxide was stirred in 1 mL of acetonitrile at 25 °C for 1 h. The product was purified by column chromatography using silica gel as the solid phase and dichloromethane:methanol (97:3) as the eluent.

#### 4.4.1 3-[(Diphenylphosphoryl)(hydroxy)methyl]-6-methyl-4H-chromen-4-one (**3**)

The title compound was isolated as a light-yellow solid (0.38 g, 98% yield). Mp: 190–192 °C; ^1^H NMR (300 MHz, CDCl_3_) δ 2.41 (s, 3H), 4.98 (s, 1H), 5.87–5.97 (m, 1H), 7.29 (d, *J*_HH_ = 4.7, 1H), 7.35–7.40 (m, 2H), 7.41–7.52 (m, 4H), 7.74–7.89 (m, 3H), 7.89–8.01 (m, 2H), 8.35–8.47 (m, 1H); ^13^C NMR (75 MHz, CDCl_3_) δ 20.9, 66.2 (d, ^1^*J*_CP_ = 97.8), 118.0, 119.3 (d, ^2^*J*_CP_ = 5.8), 123.1, 124.9, 128.4 (d, ^3^*J*_CP_ = 11.6), 128.6 (d, ^3^*J*_CP_ = 12.1), 130.3 (d, ^1^*J*_CP_ = 97.0), 131.4 (d, ^1^*J*_CP_ = 106.1), 131.6 (d, ^2^*J*_CP_ = 9.0), 132.0 (d, ^2^*J*_CP_ = 9.0), 132.1 (d, *J*_CP_ = 2.7), 132.3 (d, *J*_CP_ = 2.6), 135.2, 135.4, 154.3, 156.0 (d, ^3^*J*_CP_ = 6.7), 177.0 (d, ^3^*J*_CP_ = 5.5); ^31^P (121.5 MHz, CDCl_3_) δ 29.8; [M + H]^+^_found_ = 391.1112, C_23_H_20_NO_4_P requires: 391.1099.

### 4.5 Single-Crystal XRD Measurements

Single-crystal XRD data of **1a** were collected on an Agilent Technologies SuperNova Dual diffractometer using Cu-Kα radiation (λ = 1.54184 Å) at room temperature. The data were processed using CrysAlis Pro.^20^ The structure was solved by SHELXT^21^ using intrinsic phasing and refined by a full-matrix least-squares procedure based on *F*^2^ with SHELXL^22^ using the Olex2 program suite.^23^ All the nonhydrogen atoms were refined anisotropically. Carbon atoms C13–C15 part of the butyl substituent were found to have large thermal motions, and their atomic displacement parameters were restrained to have similar *U^ij^* components. Hydrogen atoms were readily located in difference Fourier maps and were subsequently treated as riding atoms in geometrically idealized positions. Hydrogen atoms attached to the amine nitrogen atom were refined restraining the bonding distance. The crystallographic data are listed in Table S1.

### 4.6 DFT Calculations

All calculations have been carried out with the Gaussian16^24^ and MRCC program^25^ packages. Full geometry optimization was performed at the ωB97XD/6-31G* level of theory for each molecule. Minima are characterized by only positive eigenvalues, and transition states by a single negative eigenvalue of the Hessian. As the molecular number changes during reactions, the Gibbs free energies should be considered to describe the thermodynamic and kinetic profiles of these processes. On the other hand, it should be mentioned that the utilization of electronic energies was chosen on purpose, as these values were shown to be the most robust and reliable.^26^ In order to minimize the effect of the entropy (caused by the molecular number changes during reactions), the Van der Waals complex of the two investigated molecules was calculated and he energy of this complex was used during the evaluation of reaction energies and activation barriers. **1a** and **2a** (and their derivatives) were calculated with ethyl substituents at the nitrogen atoms (instead of the *n*-butyl) to reduce the computational time.

### 4.7 Biological Evaluation

#### 4.7.1 Cell Culture

Cells were purchased from the American Type Culture Collection (ATCC, Manassas, Virginia, USA). Human lung adenocarcinoma A549 cells were maintained in Dulbecco’s modified Eagle’s medium (DMEM), while mouse fibroblast NIH/3T3 and human promyelocytic leukemia HL-60 cells were maintained in Roswell Park Memorial Institute 1640 medium (RPMI-1640) containing 10% FCS. Media were supplemented with 2 mM GlutaMAX, 100 U/mL penicillin, and 100 μg/mL streptomycin (Life Technologies, Carlsbad, California, USA). Cell cultures were maintained at 37 °C in a humidified incubator in an atmosphere of 5% CO_2_ (Sanyo, Japan).

#### 4.7.2 Cytotoxicity Assay

Cytotoxicity of the synthesized molecules was determined on A549, NIH/3T3, and HL-60 cells using the fluorescent Resazurin assay as described previously.^19^

Briefly, cells (A549 and NIH/3T3: 6000, HL-60: 120.000 cells/well) were seeded into 96-well plates (Corning Life Sciences) in media and incubated overnight. Test compounds were dissolved in 3-fold amounts of dimethyl sulfoxide (DMSO). Cells were treated with an increasing concentration of test compounds (1–30 μM). Positive controls were doxorubicin for A549 and NIH/3T3 (IC_50_ = 0.31 ± 0.24 and 5.65 ± 0.81 μM, respectively), and bortezomib for HL60 (IC_50_ = 7.42 ± 2.60 nM). Cell viability was determined after 72 h incubation. Resazurin reagent (Sigma-Aldrich) was added at a final concentration of 25 μg/mL. After a 2 h incubation for at 37 °C, 5% CO_2_, fluorescence (530 nm excitation/580 nm emission) was recorded on a multimode microplate reader (Cytofluor4000, PerSeptive Biosytems). Viability was calculated with relation to untreated control cells and blank wells containing media without cells. IC_50_ values (50% inhibiting concentration) were calculated by GraphPad Prism 5 (La Jolla, CA, USA).

## Supporting Information Available

Additional XRD and DFT calculations data and copies of the ^1^H, ^13^C and ^31^P NMR spectra of all products (PDF)

## Accession Codes

CCDC 2202806 contains the supplementary crystallographic data for this paper. These data can be obtained free of charge via www.ccdc.cam.ac.uk/data_request/cif, or by emailing. data_request@ccdc.cam.ac.uk, or by contacting The Cambridge Crystallographic Data Centre, 12 Union Road, Cambridge CB2 1EZ, UK; fax: +44 1223 336033.
