# Supplementary material for: Three-Component Reaction of 3-Formyl-6-Methylchromone, Primary Amines, and Secondary Phosphine Oxides: A Synthetic and Mechanistic Study
Source: ACS Omega. 2022 Dec 30;8(2):2698–711. doi: 10.1021/acsomega.2c07333 (PMC9850473; doi:10.1021/acsomega.2c07333)
Supplement: Supplementary file 1 — ao2c07333_si_001.pdf [file ao2c07333_si_001.pdf]

Supporting Information

for

Three-component Reaction of 3-Formyl-6-methylchromone, Primary Amines and Secondary Phosphine Oxides: A Synthetic and Mechanistic Study

Nóra Popovics-Tóth,[a] Trinh Dang Tran Bao,[a] Ádám Tajti,[a] Béla Mátravölgyi,[a] Zsolt Kelemen,[b] Franc Perdih,[c] László Hackler Jr.,[d] László G. Puskás[d] and Erika Bálint[a*]

Address:

[a]*Department of Organic Chemistry and Technology, Budapest University of Technology and Economics, H-1111 Budapest, Budafoki út 8., Hungary*

[b]*Department of Inorganic and Analytical Chemistry, Budapest University of Technology and Economics, H-1111 Budapest, Szent Gellért tér 4., Hungary*

[c]*Faculty of Chemistry and Chemical Technology, University of Ljubljana, SI-1000 Ljubljana, Slovenia*

[d]*Anthelos Ltd. Alsó kikötő sor 11/D, 6726 Szeged, Hungary*

Email:

Erika Bálint* - balint.erika@vbk.bme.hu;

*Corresponding author

Table of contents

Single Crystal X-ray Diffraction Measurements S2

DFT Calculations S4

^{1}H NMR, ^{13}C NMR and ^{31}P NMR spectra S5

Single Crystal X-ray Diffraction Measurements

Table S1. Crystal Data and Structure Refinement for Compound 1a

	**1a**
**CCDC Number**	2202806
**Empirical formula**	$C_{27}H_{28}NO_3P$
**Formula weight**	445.47
***T*/K**	293(2)
**Crystal system**	Triclinic
**Space group**	P–1
***a*/Å**	8.9713(6)
***b*/Å**	12.6467(7)
***c*/Å**	12.7764(7)
***α*/°**	61.012(6)
***β*/°**	79.547(6)
***γ*/°**	72.655(6)
***V*/Å^3**	1209.13(15)
Z	2
D_{calc} **/g cm^{-3}**	1.224
μ**/mm^{-1}**	1.226
***F*(000)**	472.0
**Reflections collected**	8496
**Data/restraints/parameters**	4590/19/295
R_{int}	0.0203
**GOF, *S***	1.033
R_1, wR_2 [$I \geq 2\sigma(I)$]	0.0691, 0.1974
R_1, wR_2 **[all data]**	0.0812, 0.2172
$\Delta\rho_{min}$, $\Delta\rho_{max}$ [e Å^{-3}]	0.64/–0.53

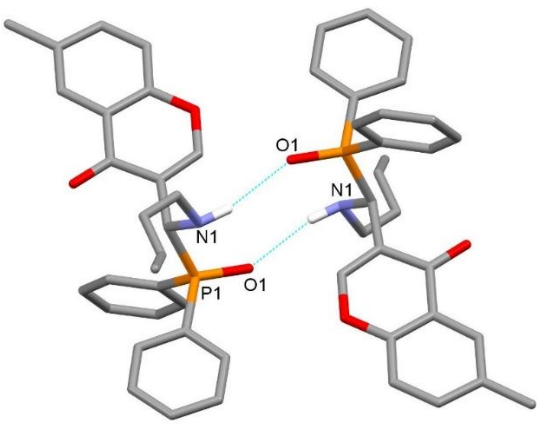

Figure S1. Hydrogen Bonded Dimer Unit in 1a *via* Centrosymmetric N–H···O=P Interaction

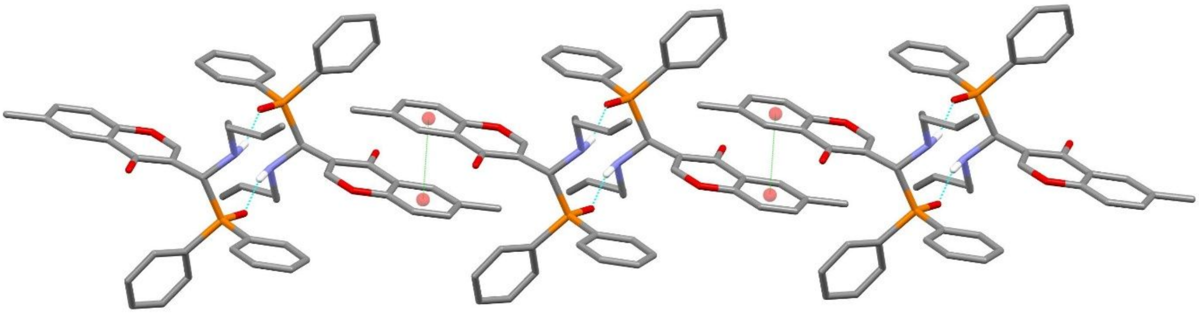

Figure S2. Layer Formation in 1a through C–H···O=P Hydrogen Bonding and π···π Interactions

DFT Calculations

Table S2. Relative Stability of 1a-1b and 2a-2b at Different Level of Theory and in kcal/mol Unit

	ωB97XD/6-31G*	ωB97XD/6-311+G**	ωB97XD/def2-TZVP	M06-2X/6-31G*	B3LYP/6-31G*	local-CCSD(T)/def2-TZVP// ωB97XD/6-31G*
ΔE_{2a-1a}	-1.0	-0.3	0.0	-1.2	-2.5	1.8
ΔE_{2b-1b}	1.1	1.1	1.9	1.9	1.9	-3.5

^{1}H NMR, ^{13}C NMR and ^{31}P NMR spectra

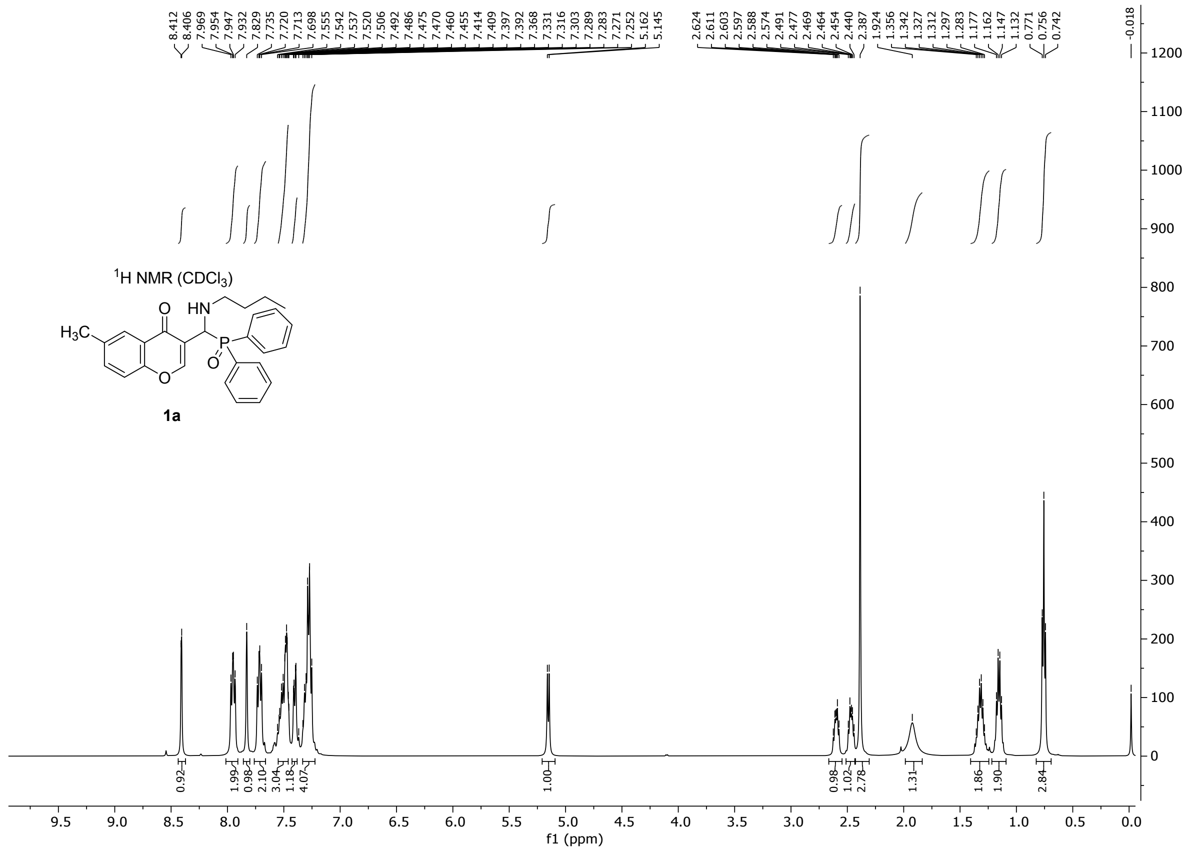

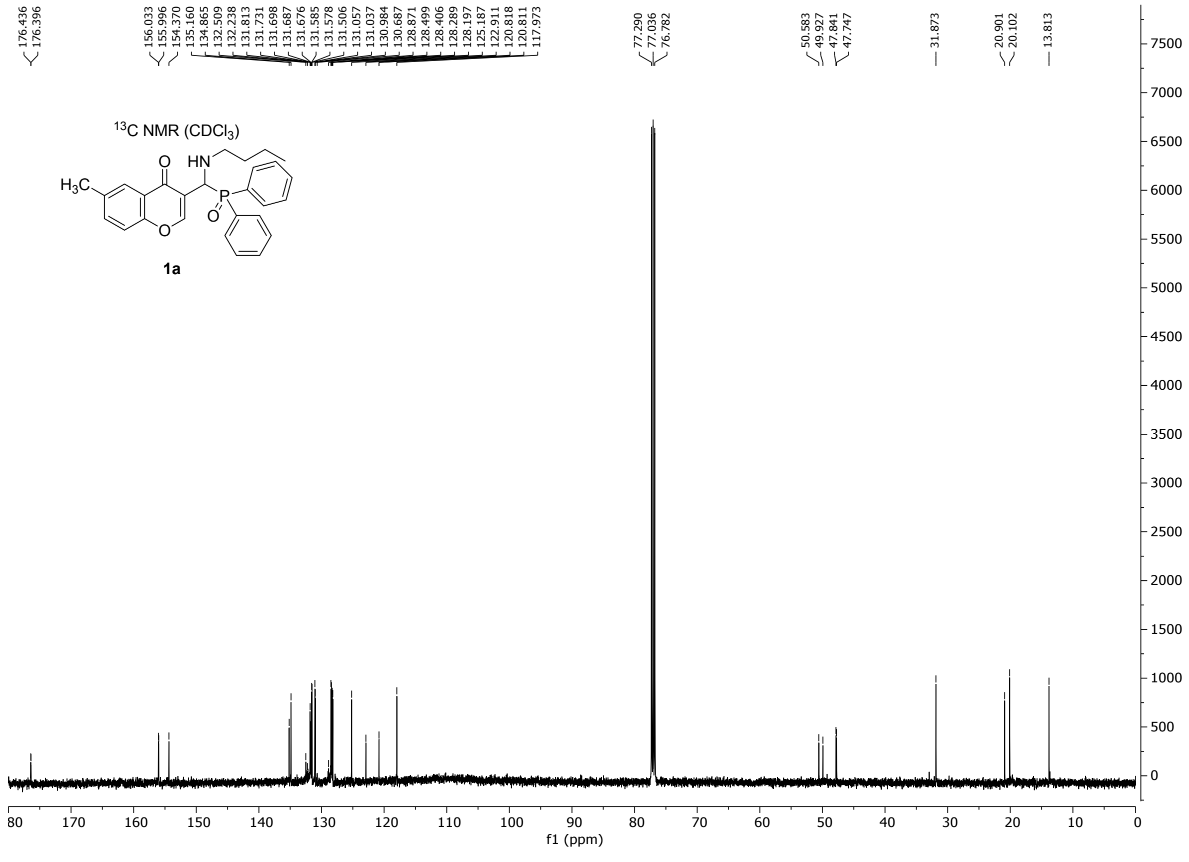

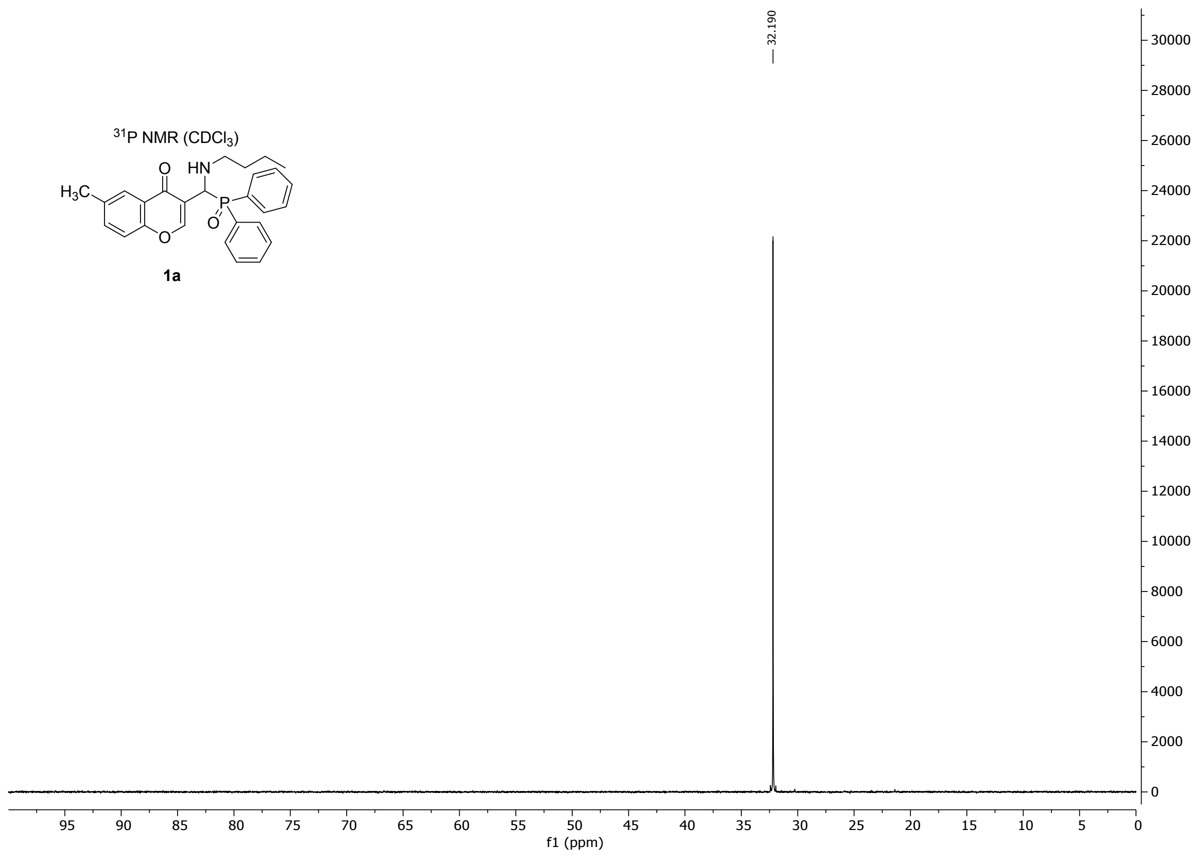

^{31}P NMR (CDCl$_3$)
1a
32.190
f1 (ppm)

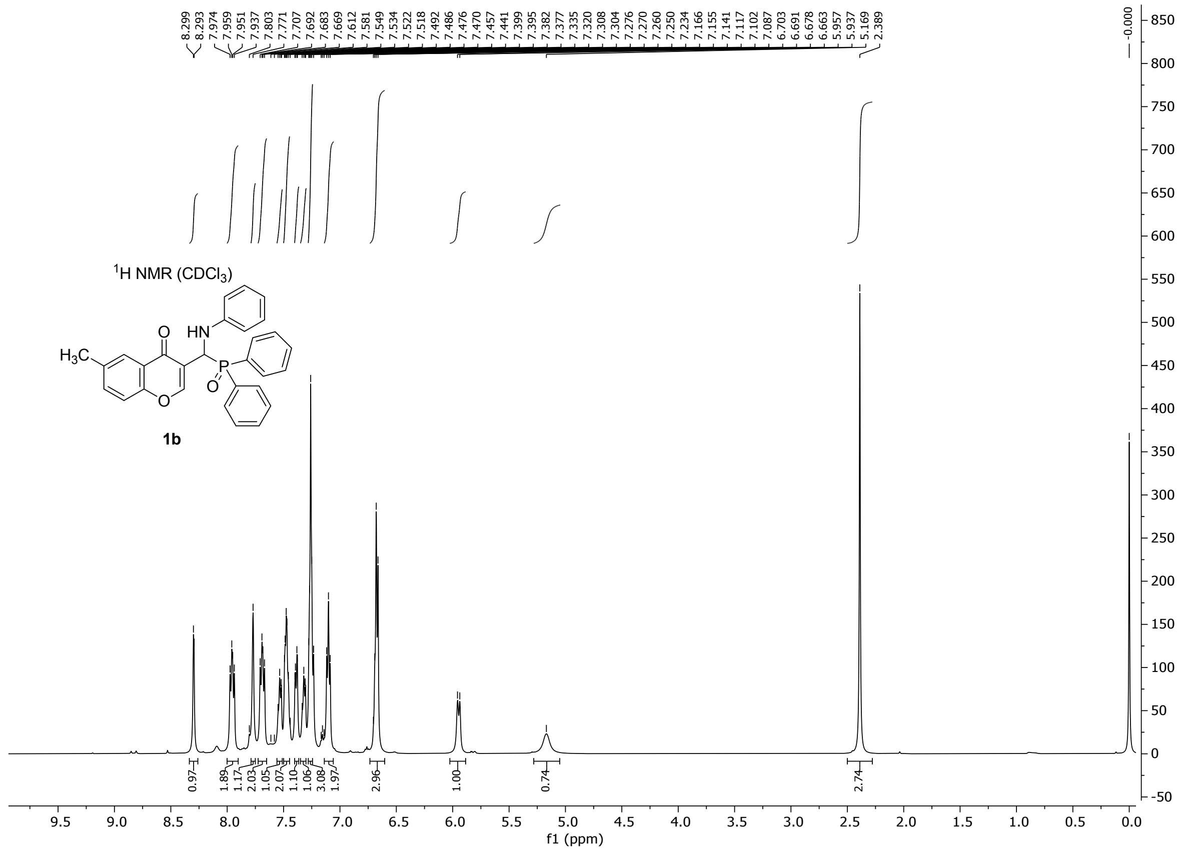

^{1}H NMR (CDCl$_3$)
1b
f1 (ppm)

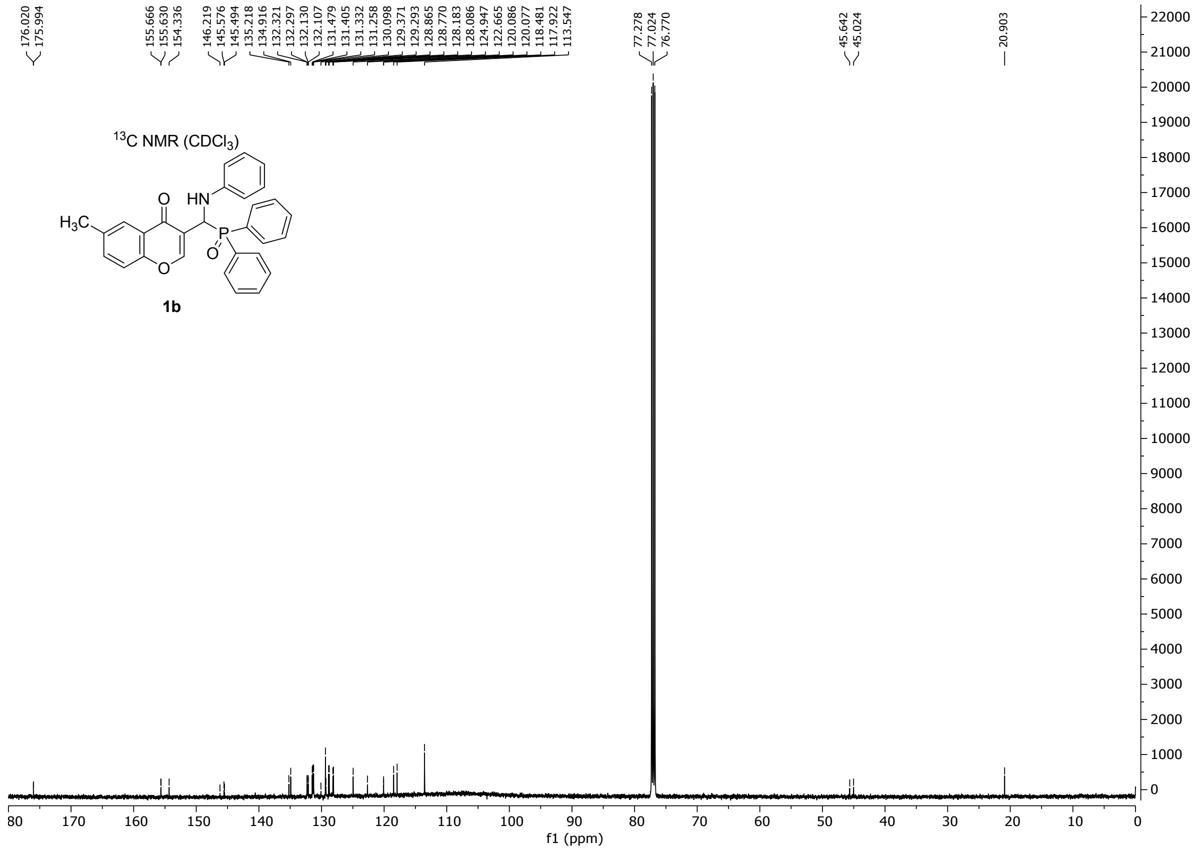
176.020
175.994
155.666
155.630
154.336
146.219
145.576
145.494
135.218
134.916
132.321
132.297
132.130
132.107
131.479
131.405
131.332
131.258
130.098
129.371
129.293
128.865
128.770
128.183
128.086
124.947
122.665
120.086
120.077
118.481
117.922
113.547
77.278
77.024
76.770
45.642
45.024
20.903
13C NMR (CDCl3)
HN
O
H3C
P
O
O
1b
f1 (ppm)

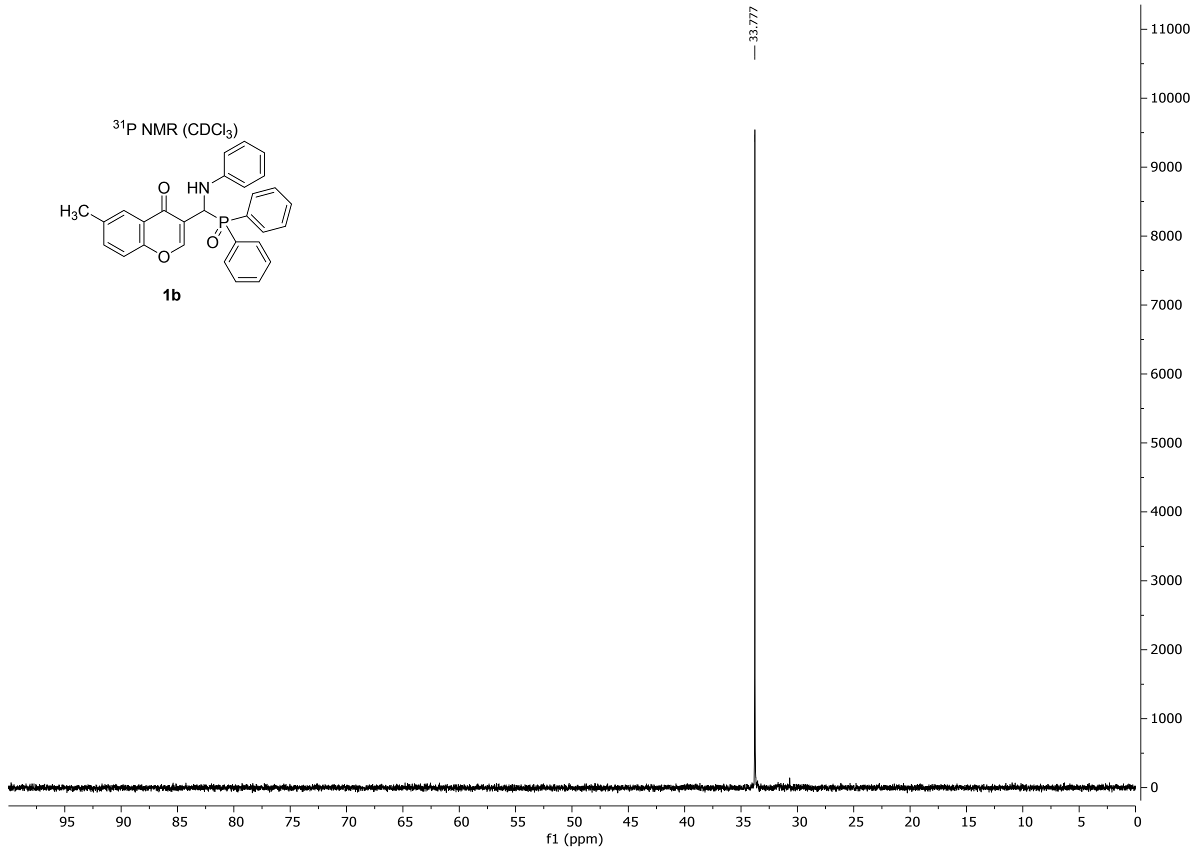
33.777
31P NMR (CDCl3)
HN
O
H3C
P
O
O
1b
f1 (ppm)

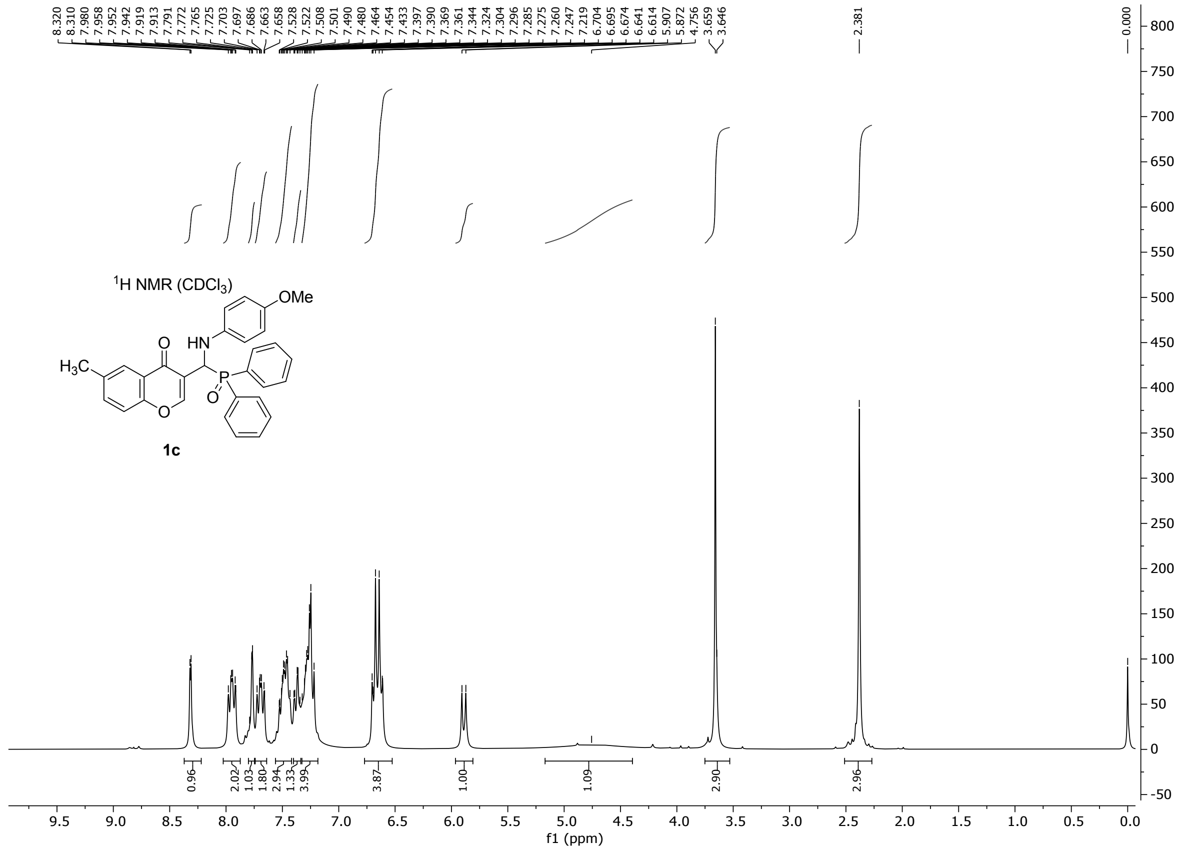
1H NMR (CDCl3)
OMe
HN
O
H3C
P
O
O
1c
f1 (ppm)

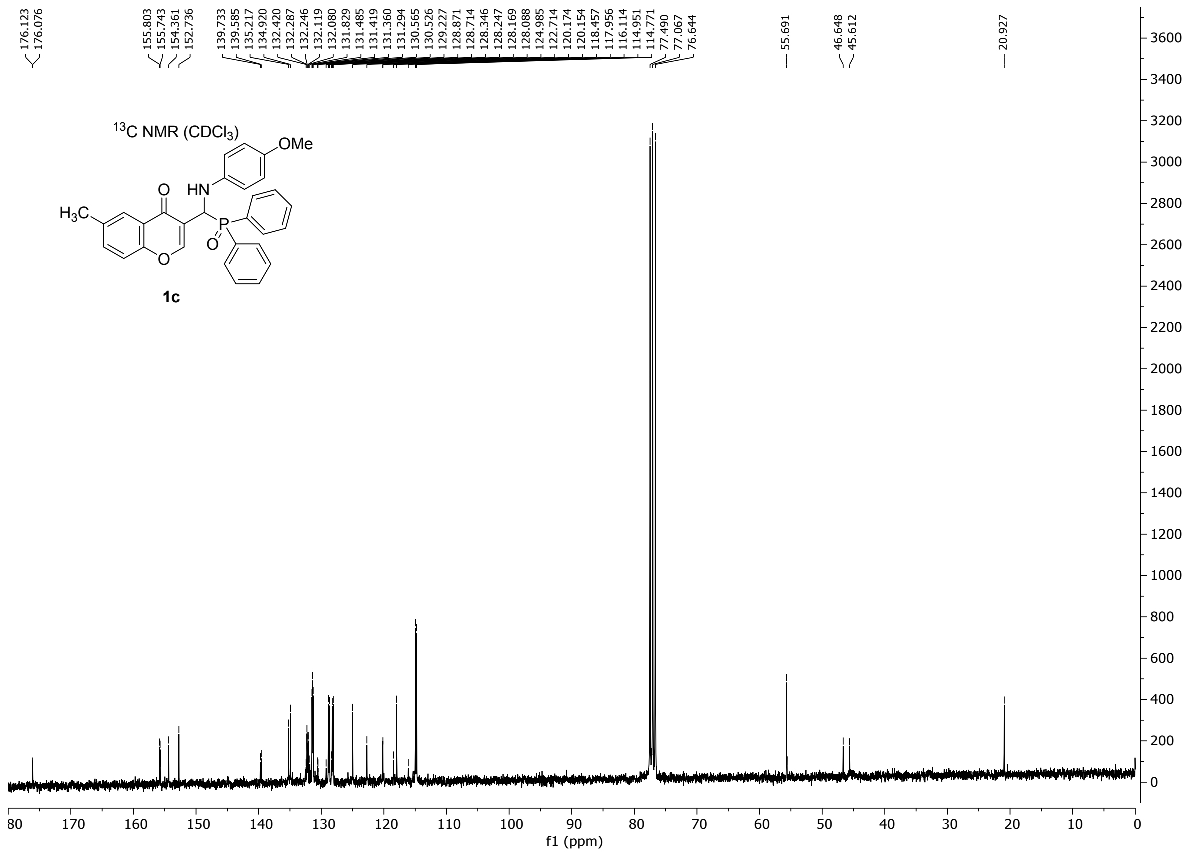
13C NMR (CDCl3)
OMe
HN
O
H3C
P
O
O
1c
f1 (ppm)

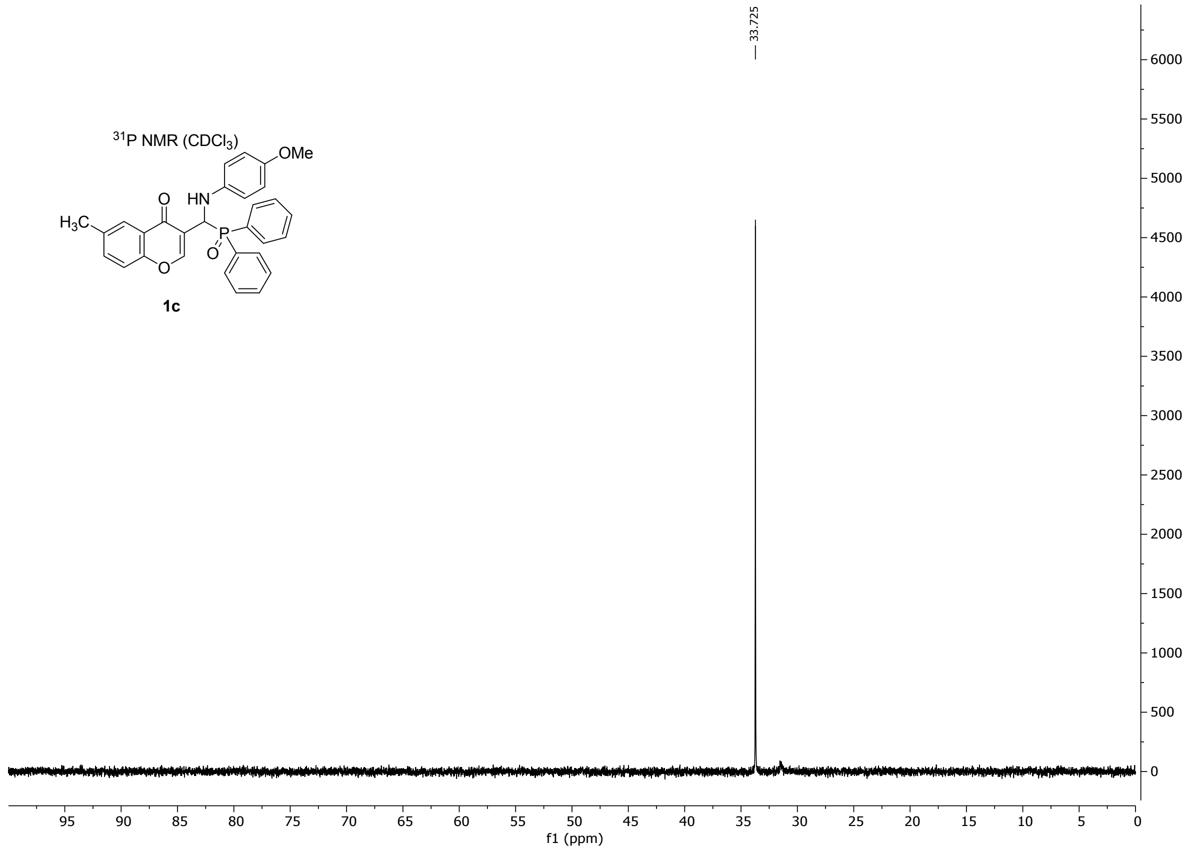

31P NMR (CDCl3)
OMe
HN
O
H3C
P
O
1c
33.725
f1 (ppm)

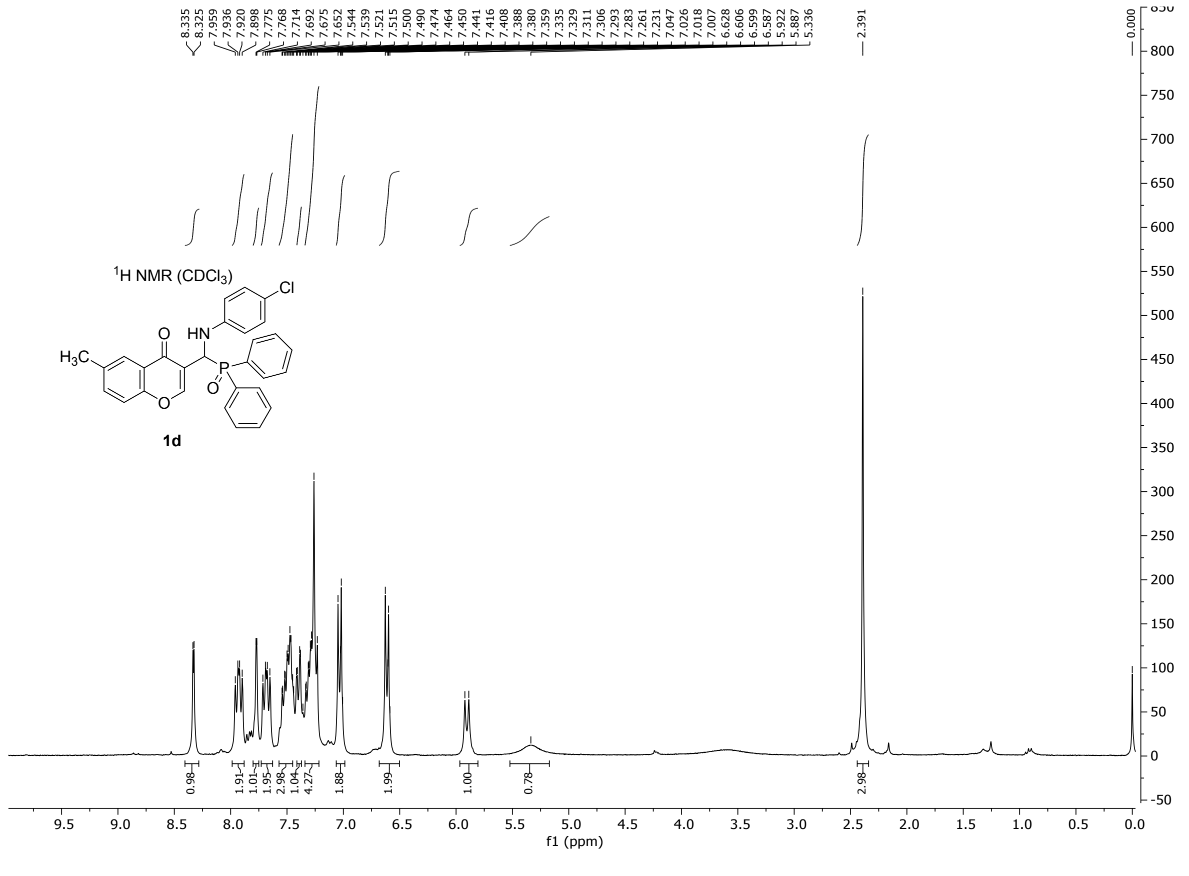

1H NMR (CDCl3)
Cl
HN
O
H3C
P
O
1d
2.391
0.000
f1 (ppm)

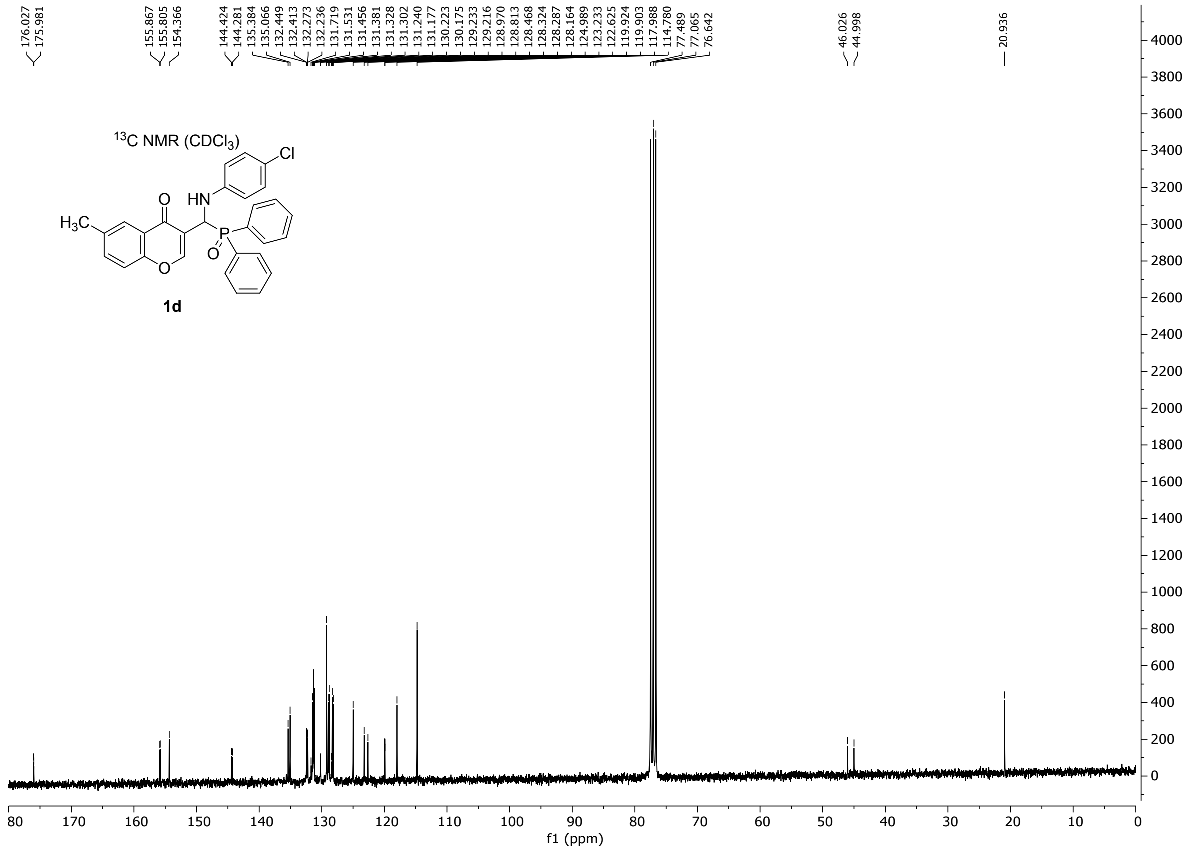
13C NMR (CDCl3)
1d
f1 (ppm)

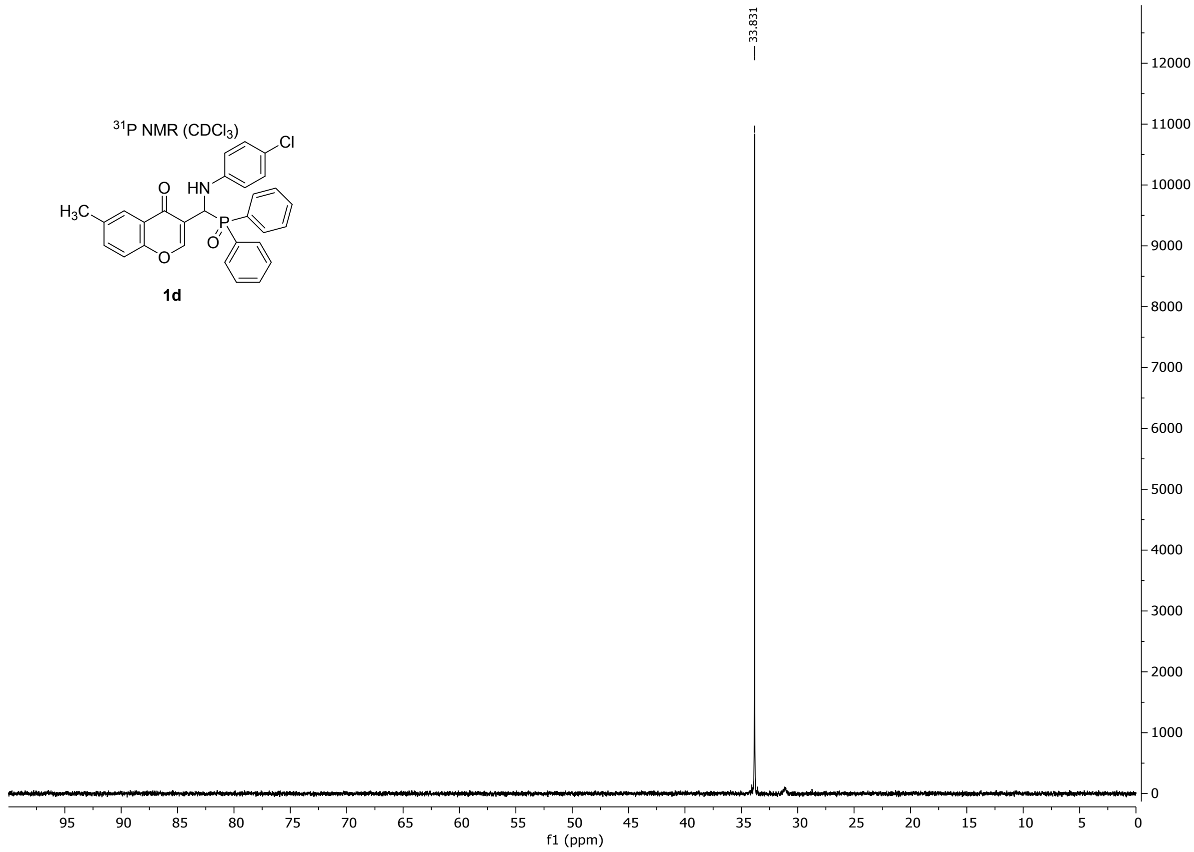
31P NMR (CDCl3)
1d
33.831
f1 (ppm)

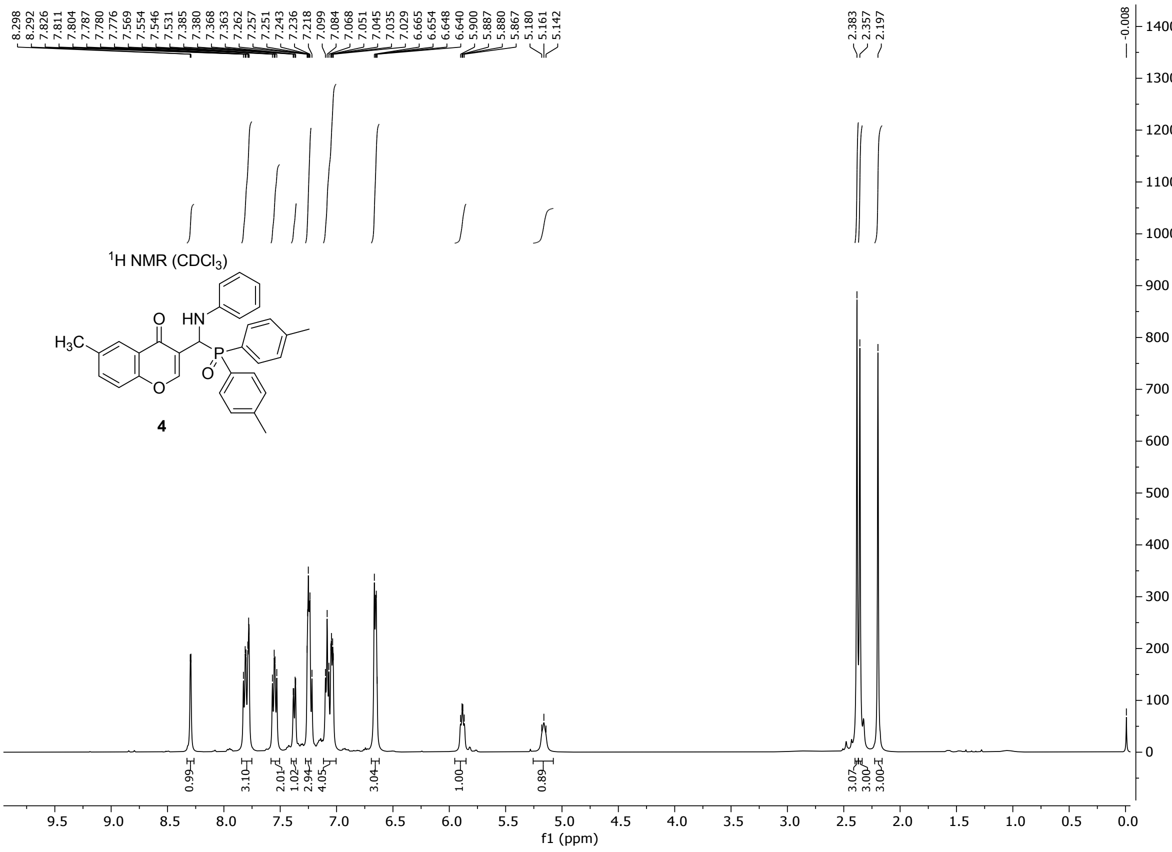
8.298 8.292 7.826 7.811 7.804 7.787 7.780 7.776 7.569 7.554 7.546 7.531 7.385 7.380 7.368 7.363 7.262 7.257 7.251 7.243 7.236 7.218 7.099 7.084 7.068 7.051 7.045 7.035 7.029 6.665 6.654 6.648 6.640 5.900 5.887 5.880 5.867 5.180 5.161 5.142
2.383 2.357 2.197
-0.008
^{1}H NMR (CDCl$_3$)
4
0.99 3.10 2.01 1.02 2.94 4.05 3.04 1.00 0.89 3.07 3.00 3.00
f1 (ppm)

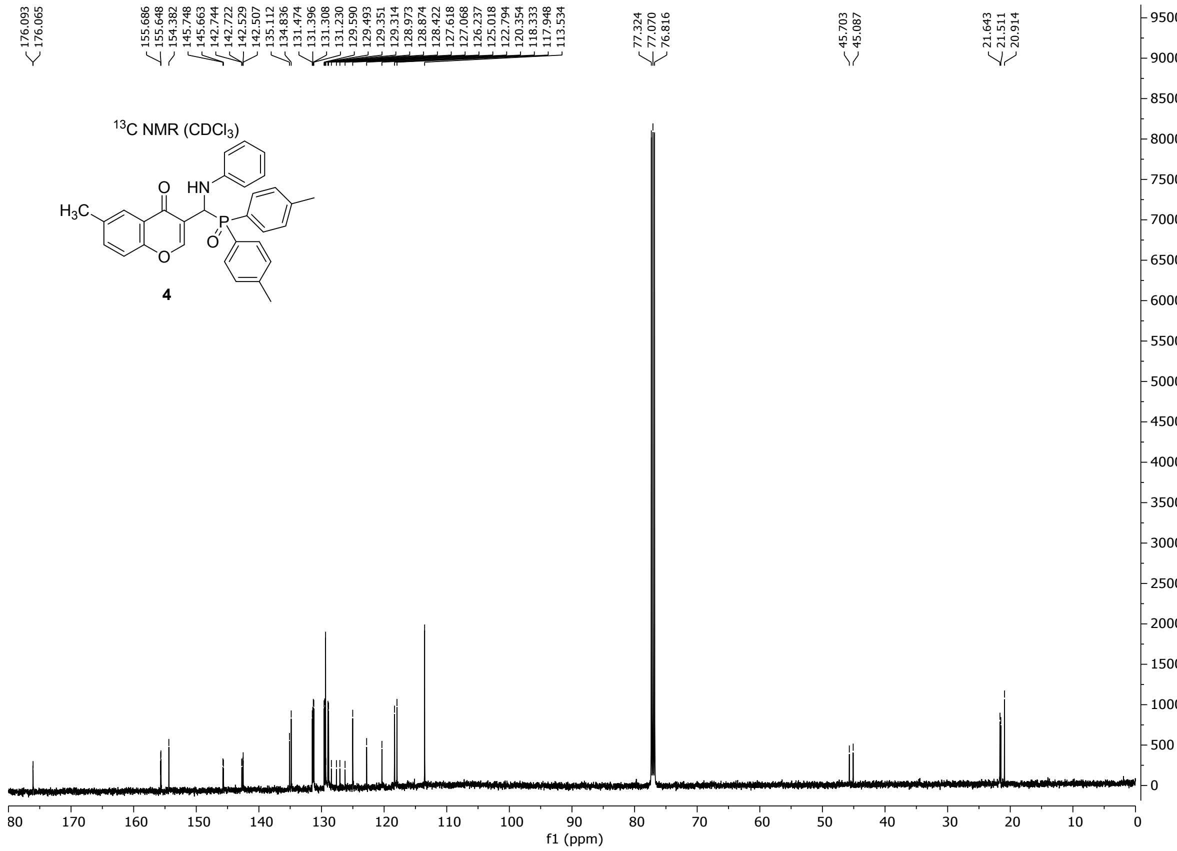
176.093 176.065
155.686 155.648 154.382 145.748 145.663 142.744 142.722 142.529 142.507 135.112 134.836 131.474 131.396 131.308 131.230 129.590 129.493 129.351 129.314 128.973 128.874 128.422 127.618 127.068 126.237 125.018 122.794 120.354 118.333 117.948 113.534
77.324 77.070 76.816
45.703 45.087
21.643 21.511 20.914
^{13}C NMR (CDCl$_3$)
4
f1 (ppm)

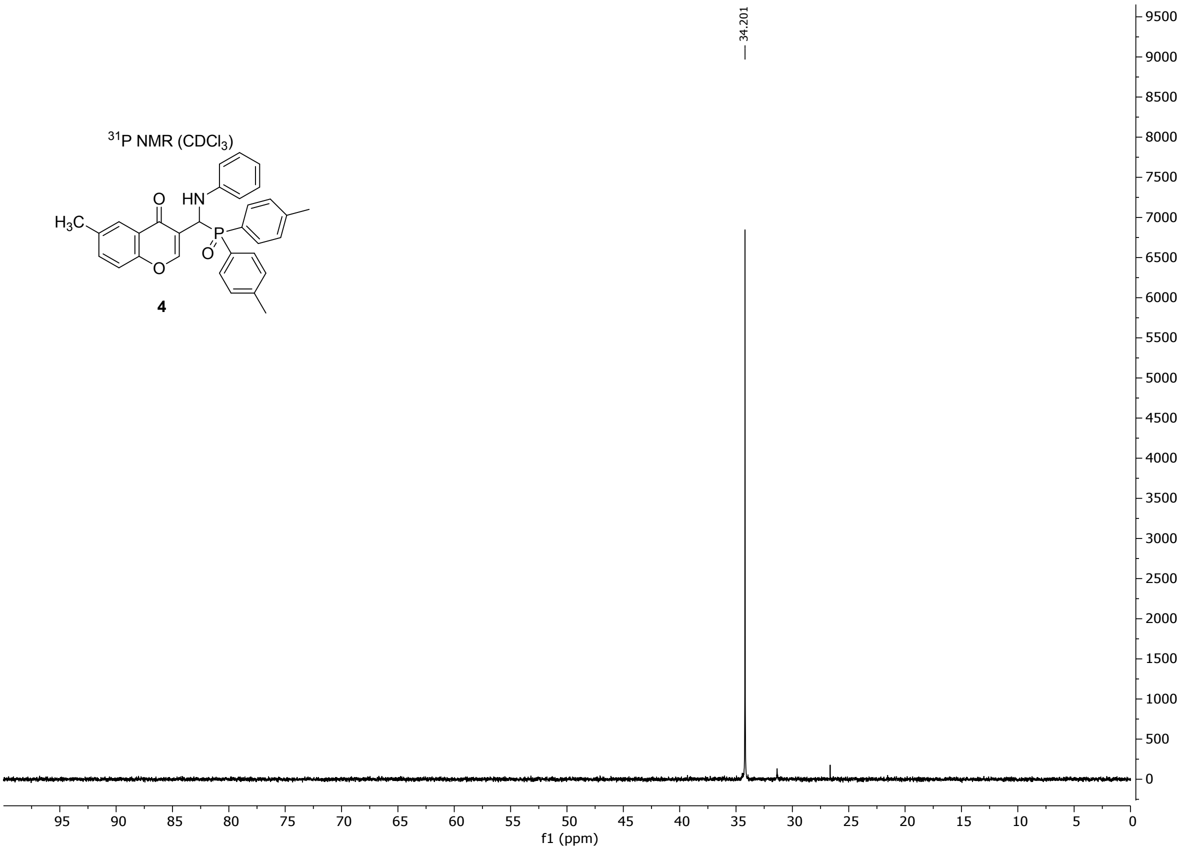

34.201
31P NMR (CDCl3)
H3C
O
HN
P
O
O
4
f1 (ppm)

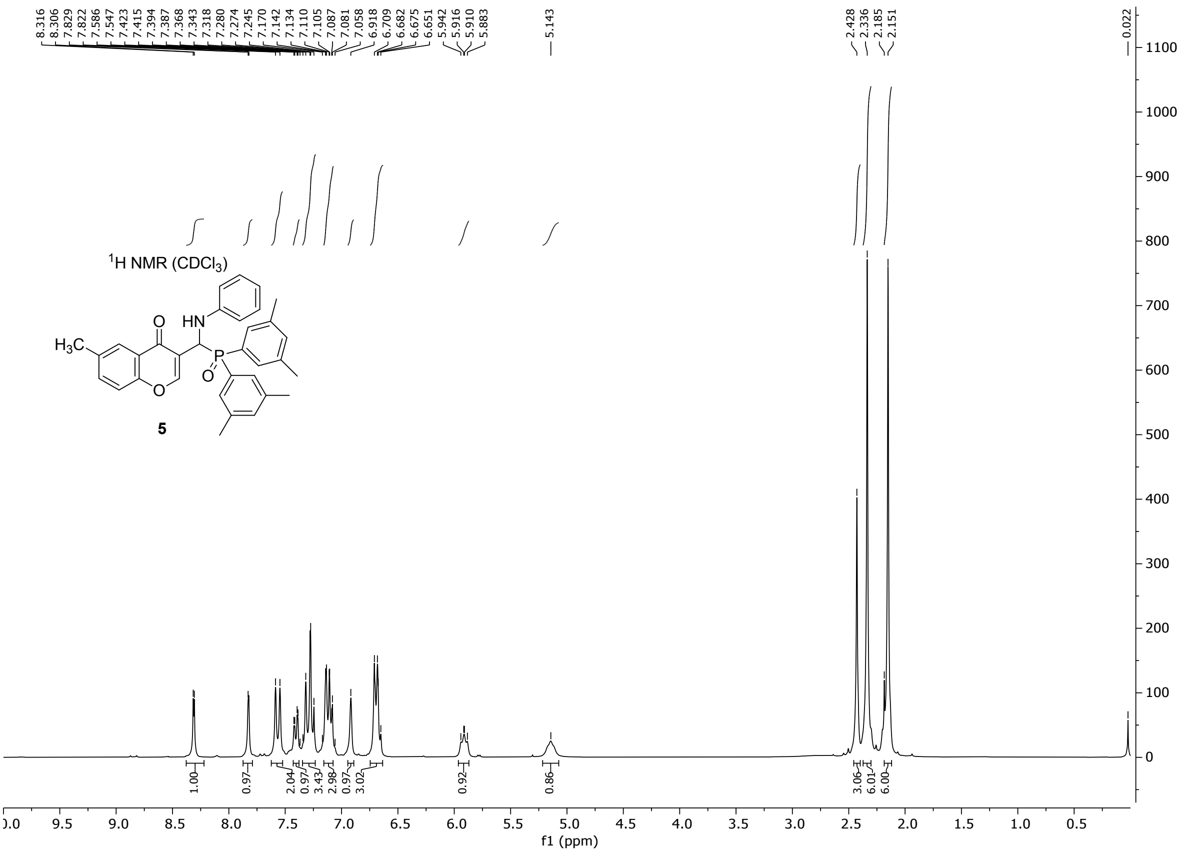

8.316 8.306 7.829 7.822 7.586 7.547 7.423 7.415 7.394 7.387 7.368 7.343 7.318 7.280 7.274 7.245 7.170 7.142 7.134 7.110 7.105 7.087 7.081 7.058 6.918 6.709 6.682 6.675 6.651 5.942 5.916 5.910 5.883
5.143
2.428 2.336 2.185 2.151
0.022
1H NMR (CDCl3)
H3C
O
HN
P
O
O
5
1.00 0.97 2.04 0.97 3.43 2.98 0.97 3.02 0.92 0.86 3.06 6.01 6.00
f1 (ppm)

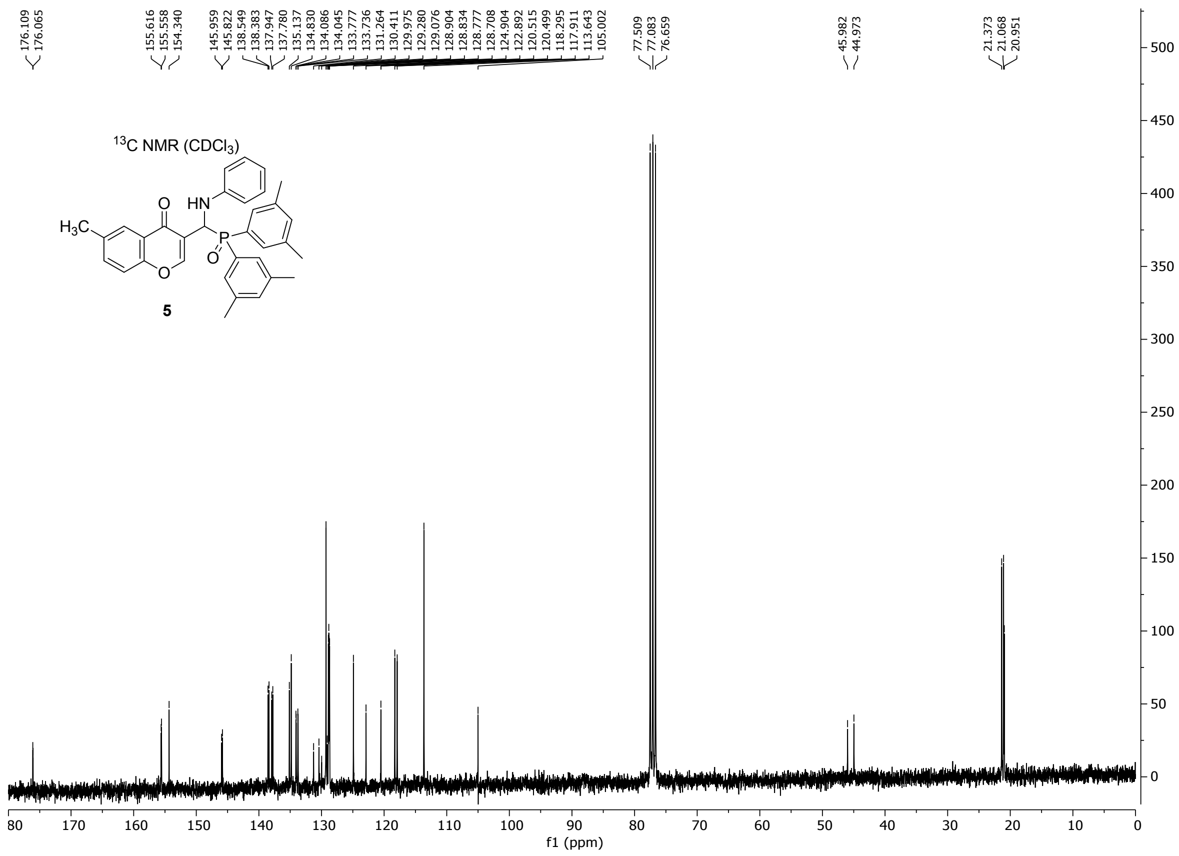

176.109
176.065
155.616
155.558
154.340
145.959
145.822
138.549
138.383
137.947
137.780
135.137
134.830
134.086
134.045
133.777
133.736
131.264
130.411
129.975
129.280
129.076
128.904
128.834
128.777
128.708
124.904
122.892
120.515
120.499
118.295
117.911
113.643
105.002
77.509
77.083
76.659
45.982
44.973
21.373
21.068
20.951
^{13}C NMR ($CDCl_3$)
5
f1 (ppm)

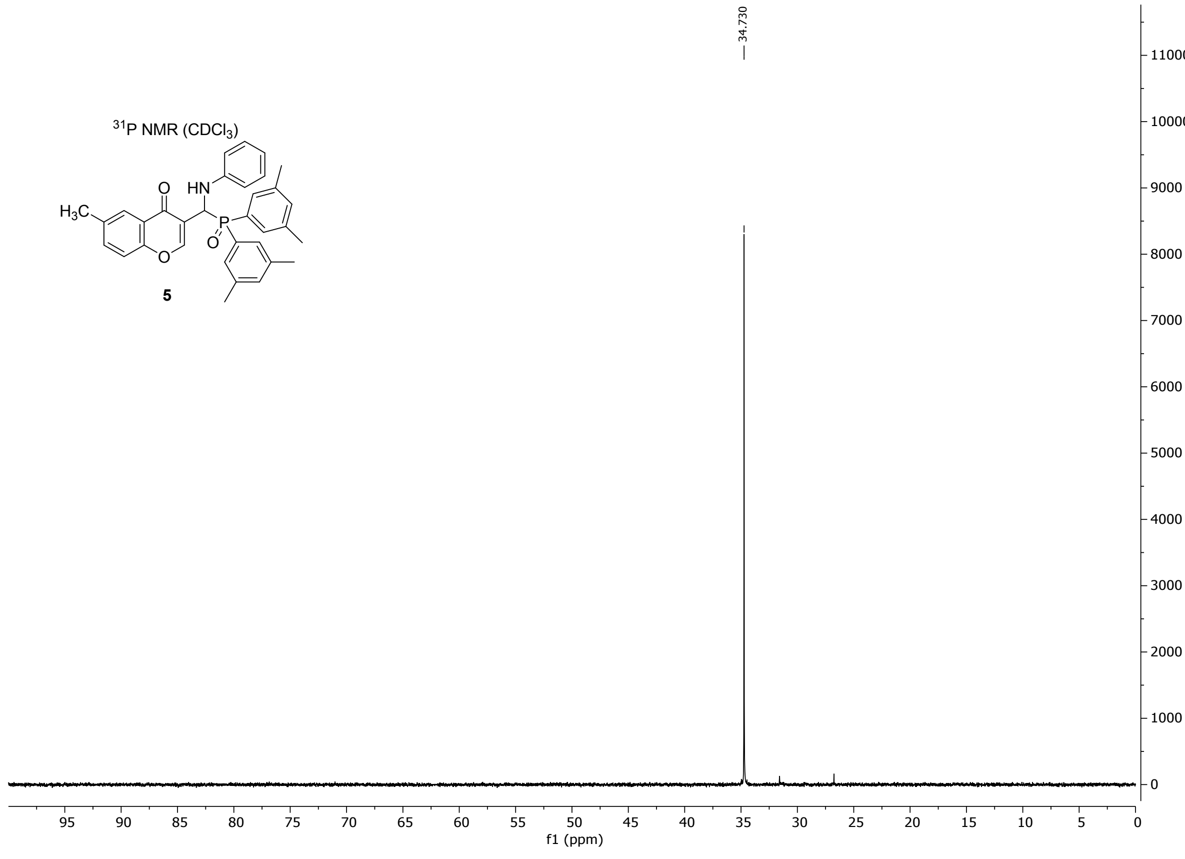

34.730
^{31}P NMR ($CDCl_3$)
5
f1 (ppm)

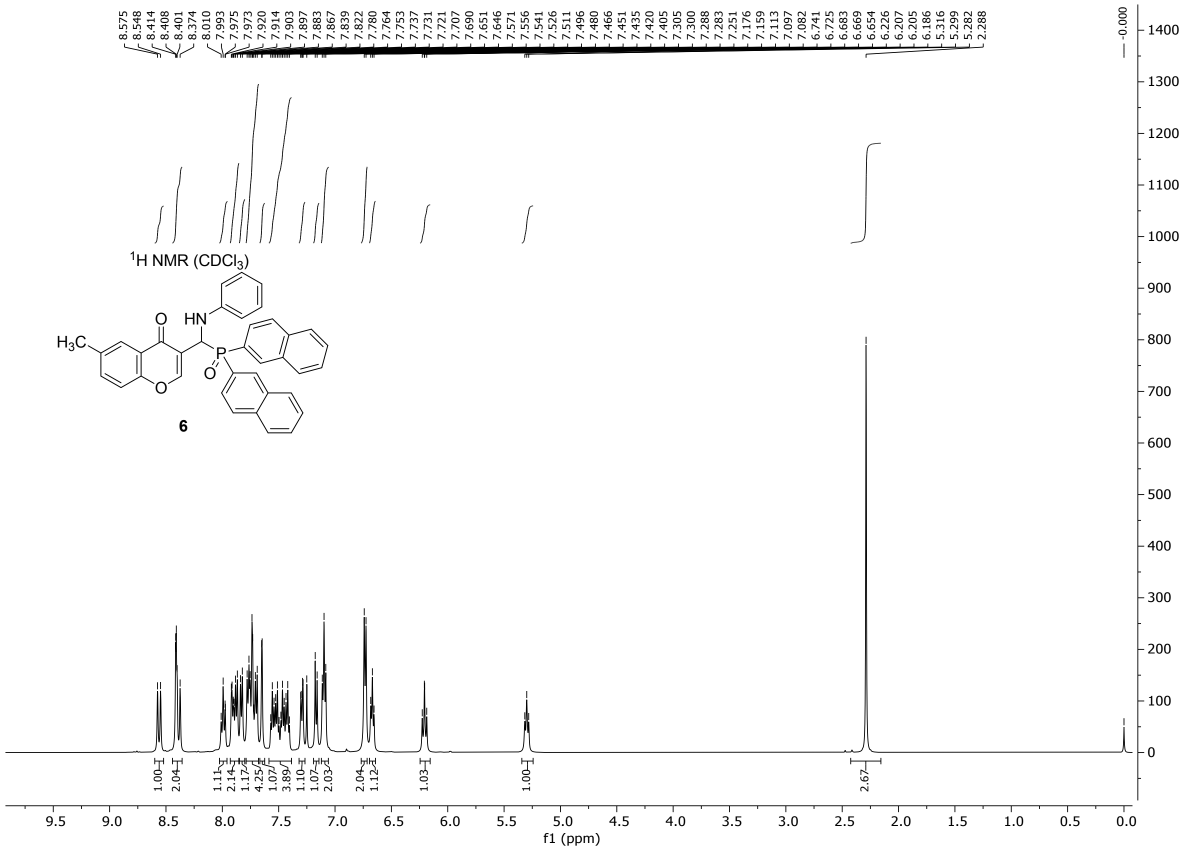
8.575
8.548
8.414
8.408
8.401
8.374
8.010
7.993
7.975
7.973
7.920
7.914
7.903
7.897
7.883
7.867
7.839
7.822
7.780
7.764
7.753
7.737
7.731
7.721
7.707
7.690
7.651
7.646
7.571
7.556
7.541
7.526
7.511
7.496
7.480
7.466
7.451
7.435
7.420
7.405
7.305
7.300
7.288
7.283
7.251
7.176
7.159
7.113
7.097
7.082
6.741
6.725
6.683
6.669
6.654
6.226
6.207
6.205
6.186
5.316
5.299
5.282
2.288
-0.000
1H NMR (CDCl3)
O
HN
H3C
P
O
O
6
1.00
2.04
1.11
2.14
1.17
1.17
4.25
1.07
3.89
1.10
1.07
2.03
2.04
1.12
1.03
1.00
2.67
f1 (ppm)

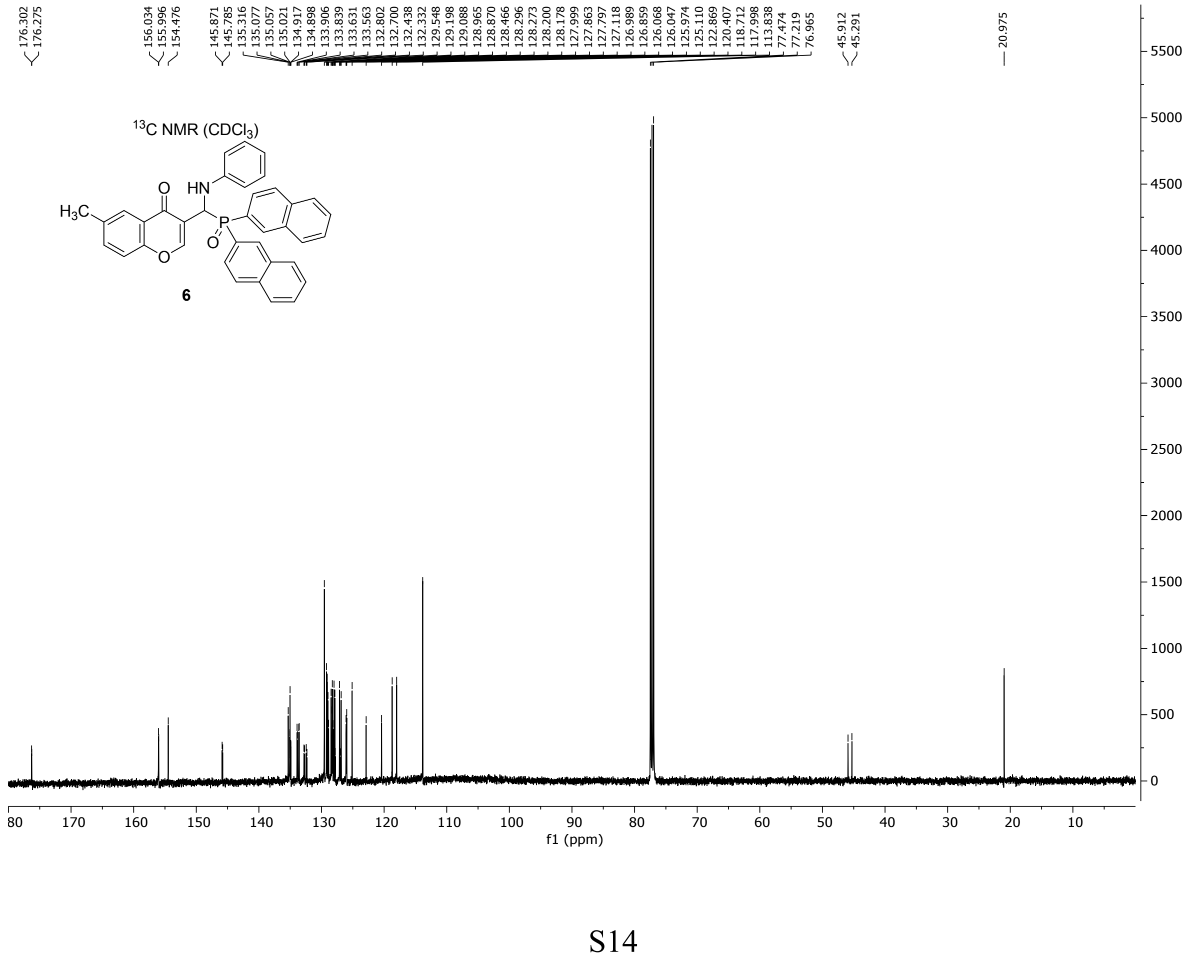
176.302
176.275
156.034
155.996
154.476
145.871
145.785
135.316
135.077
135.057
135.021
134.917
134.898
133.906
133.839
133.631
133.563
132.802
132.700
132.438
132.332
129.548
129.198
129.088
128.965
128.870
128.466
128.296
128.273
128.200
128.178
127.999
127.863
127.797
127.118
126.989
126.859
126.068
126.047
125.974
125.110
122.869
120.407
118.712
117.998
113.838
77.474
77.219
76.965
45.912
45.291
20.975
13C NMR (CDCl3)
O
HN
H3C
P
O
O
6
f1 (ppm)

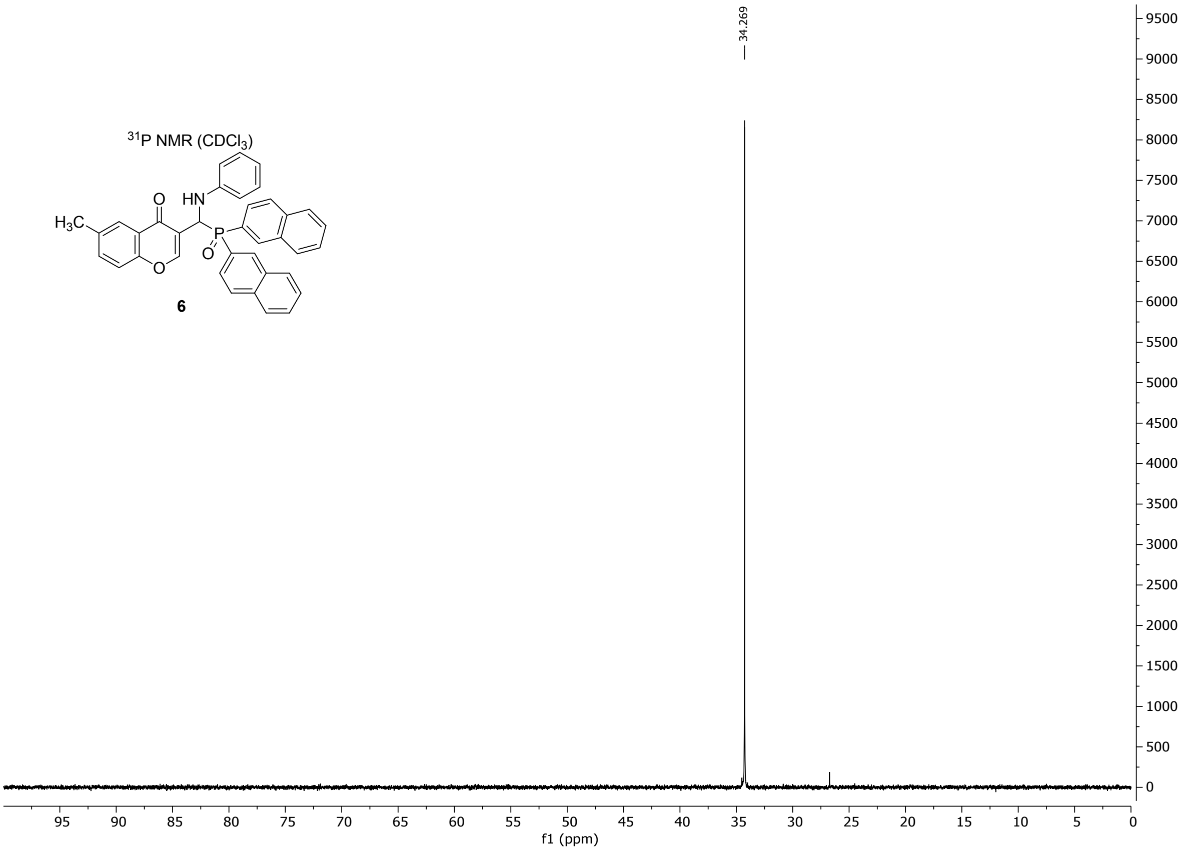

6

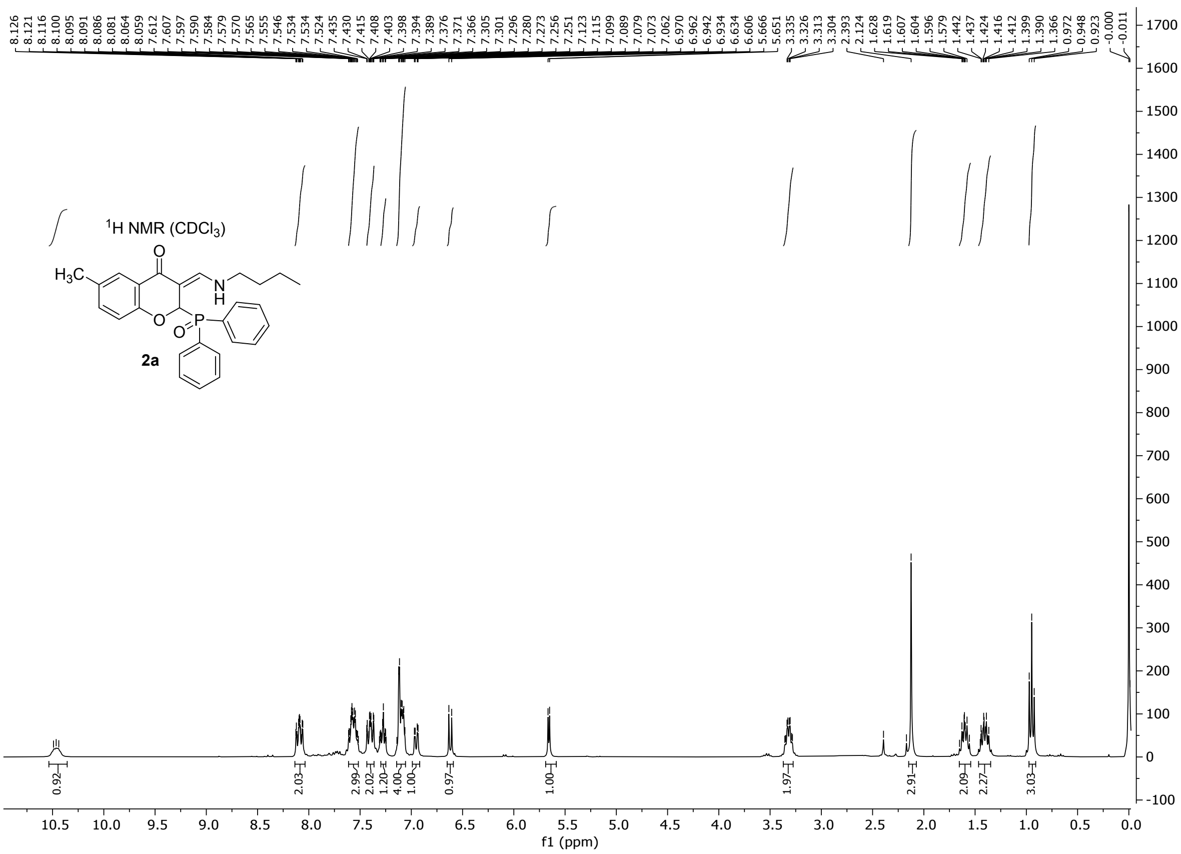

2a

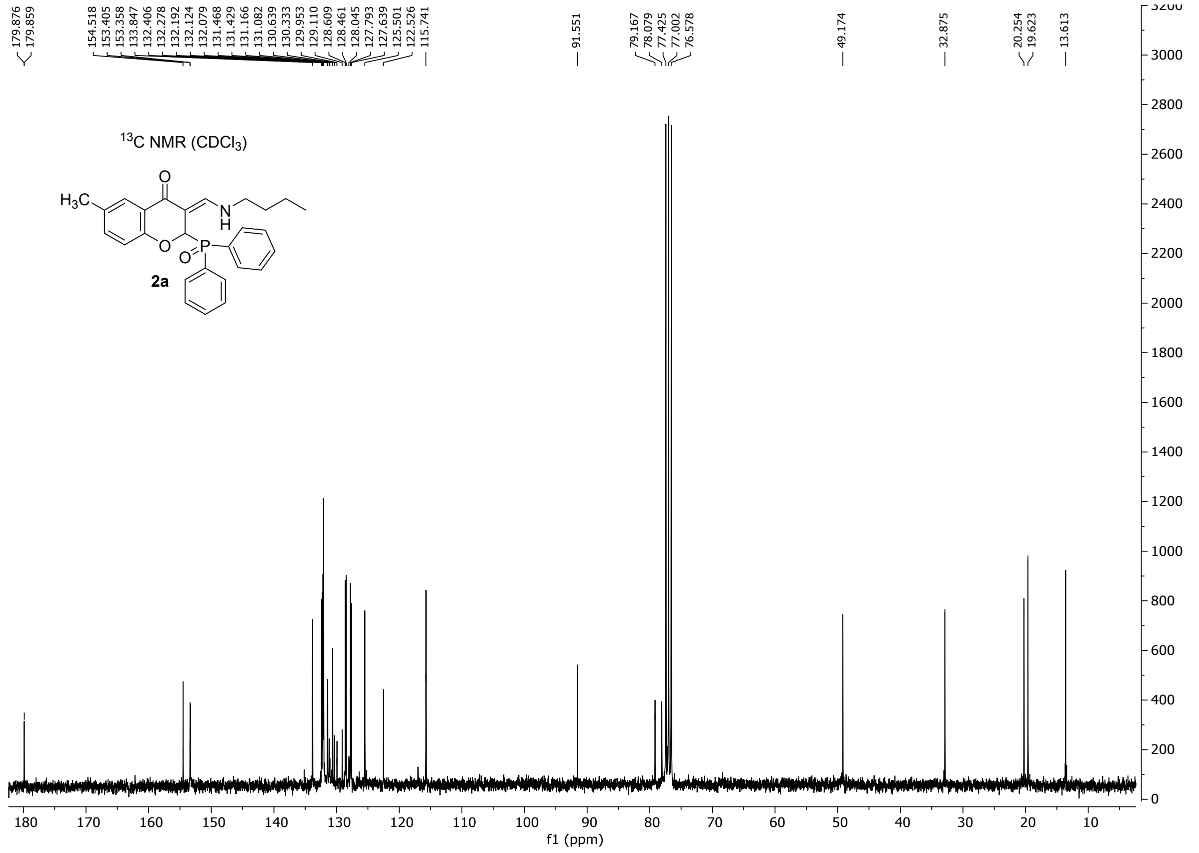

13C NMR (CDCl3)
2a
f1 (ppm)

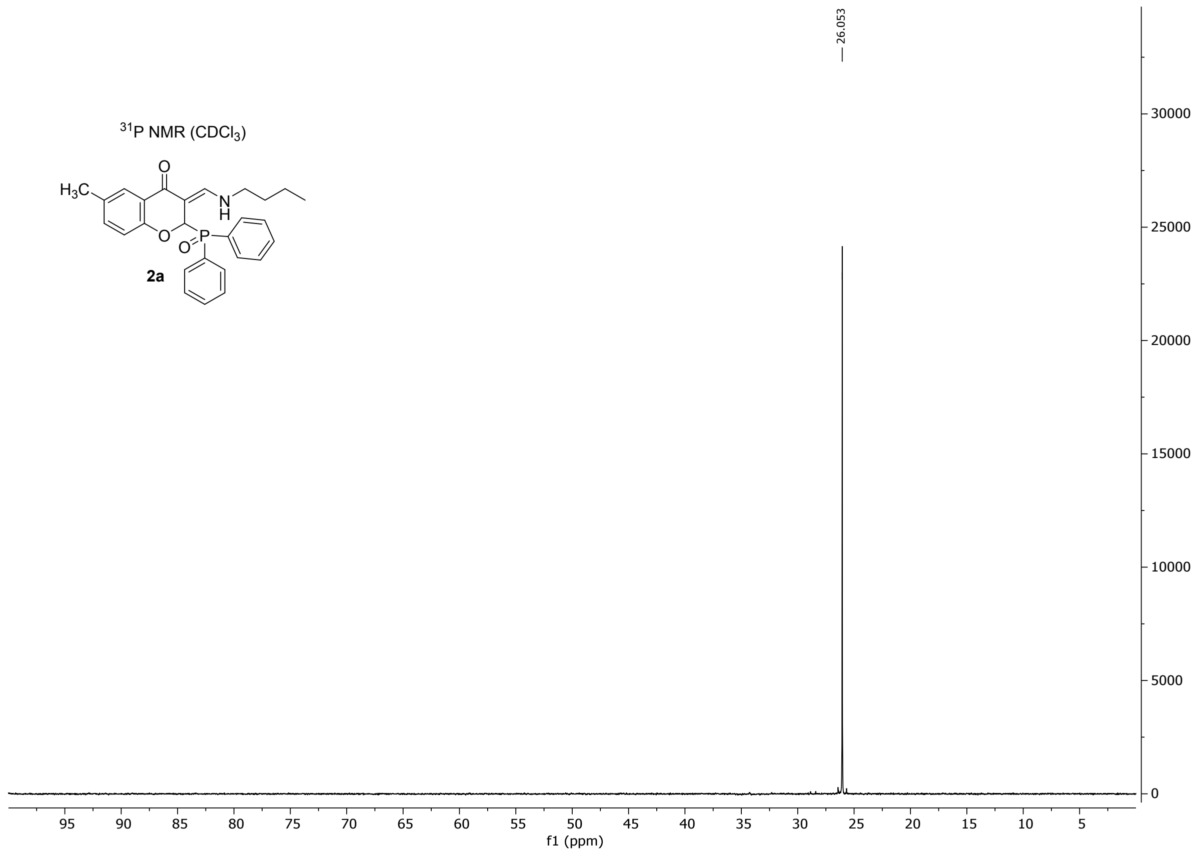

31P NMR (CDCl3)
2a
26.053
f1 (ppm)

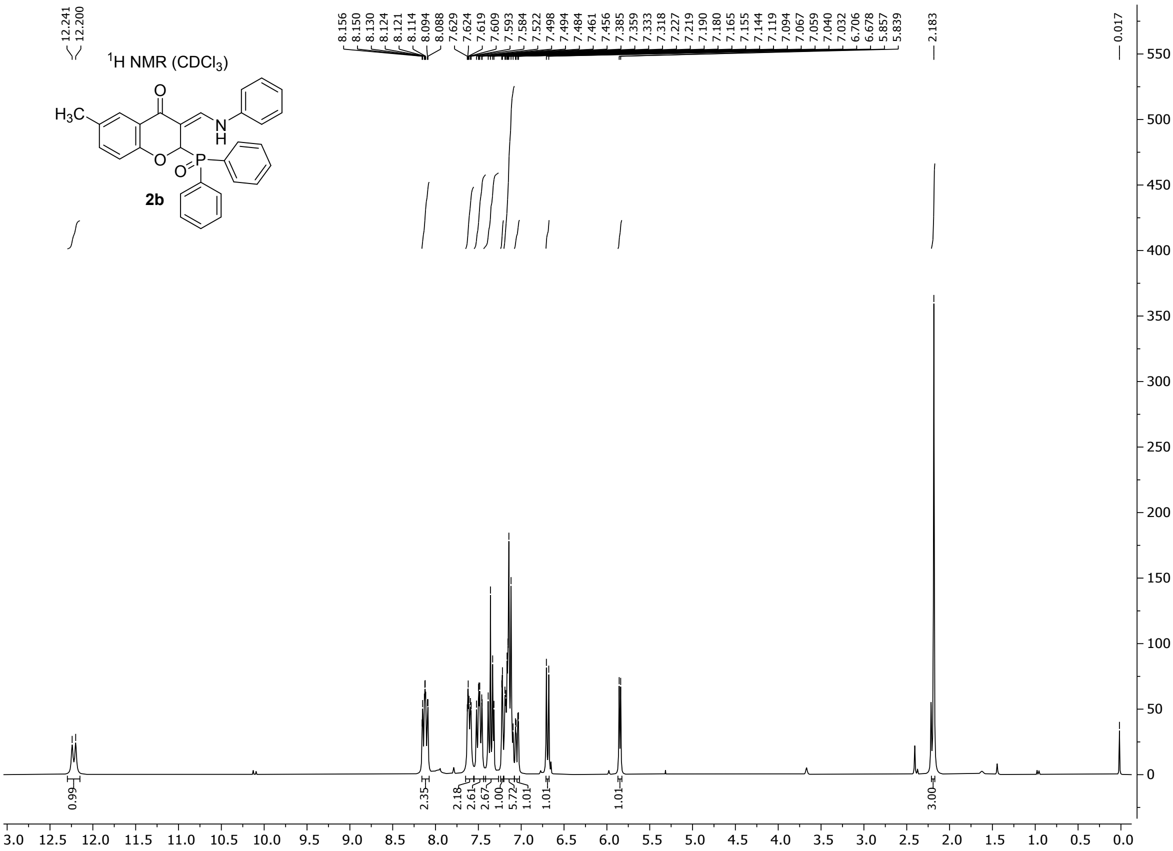
1H NMR (CDCl3)
2b
12.241
12.200
8.156
8.150
8.130
8.124
8.121
8.114
8.094
8.088
7.629
7.624
7.619
7.609
7.593
7.584
7.522
7.498
7.494
7.484
7.461
7.456
7.385
7.359
7.333
7.318
7.227
7.219
7.190
7.180
7.165
7.155
7.144
7.119
7.094
7.067
7.059
7.040
7.032
6.706
6.678
5.857
5.839
2.183
0.017
0.99
2.35
2.18
2.61
2.67
1.00
5.72
1.01
1.01
3.00

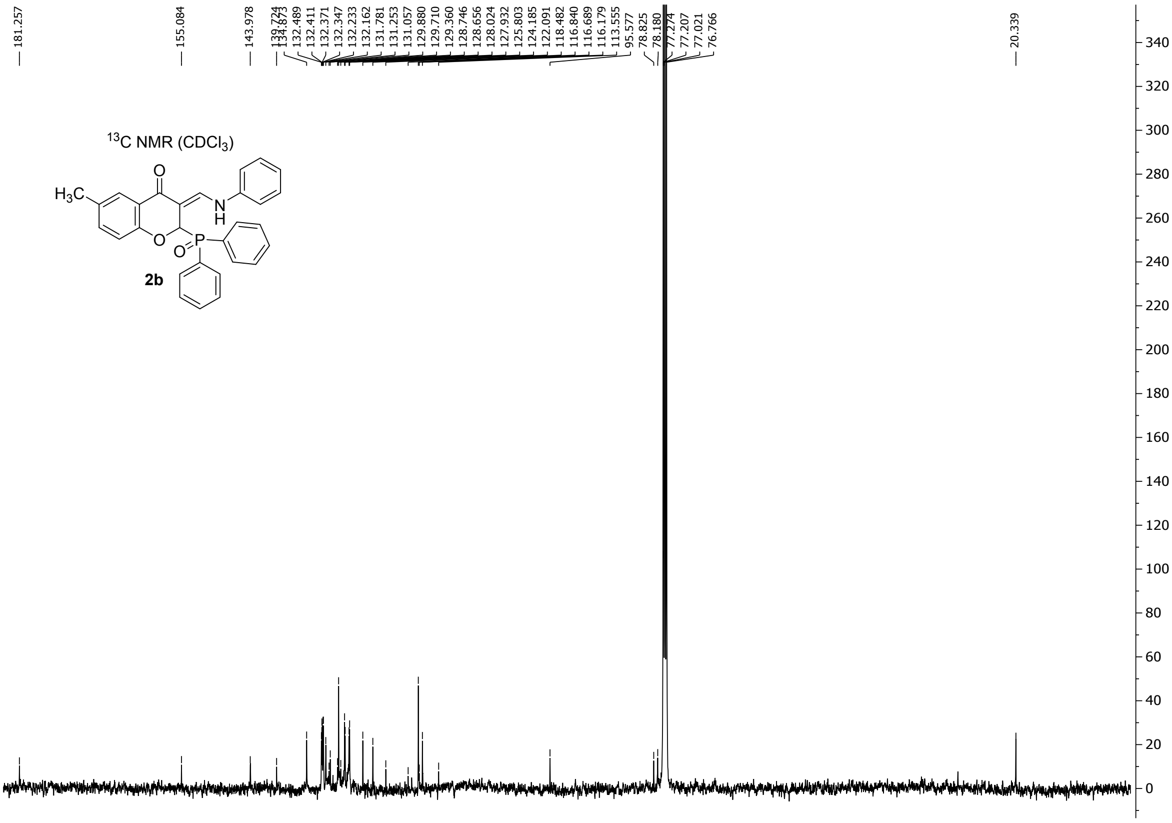
13C NMR (CDCl3)
2b
181.257
155.084
143.978
139.724
134.873
132.489
132.411
132.371
132.347
132.233
132.162
131.781
131.253
131.057
129.880
129.710
129.360
128.746
128.656
128.024
127.932
125.803
124.185
122.091
118.482
116.840
116.689
116.179
113.555
95.577
78.825
78.180
77.274
77.207
77.021
76.766
20.339

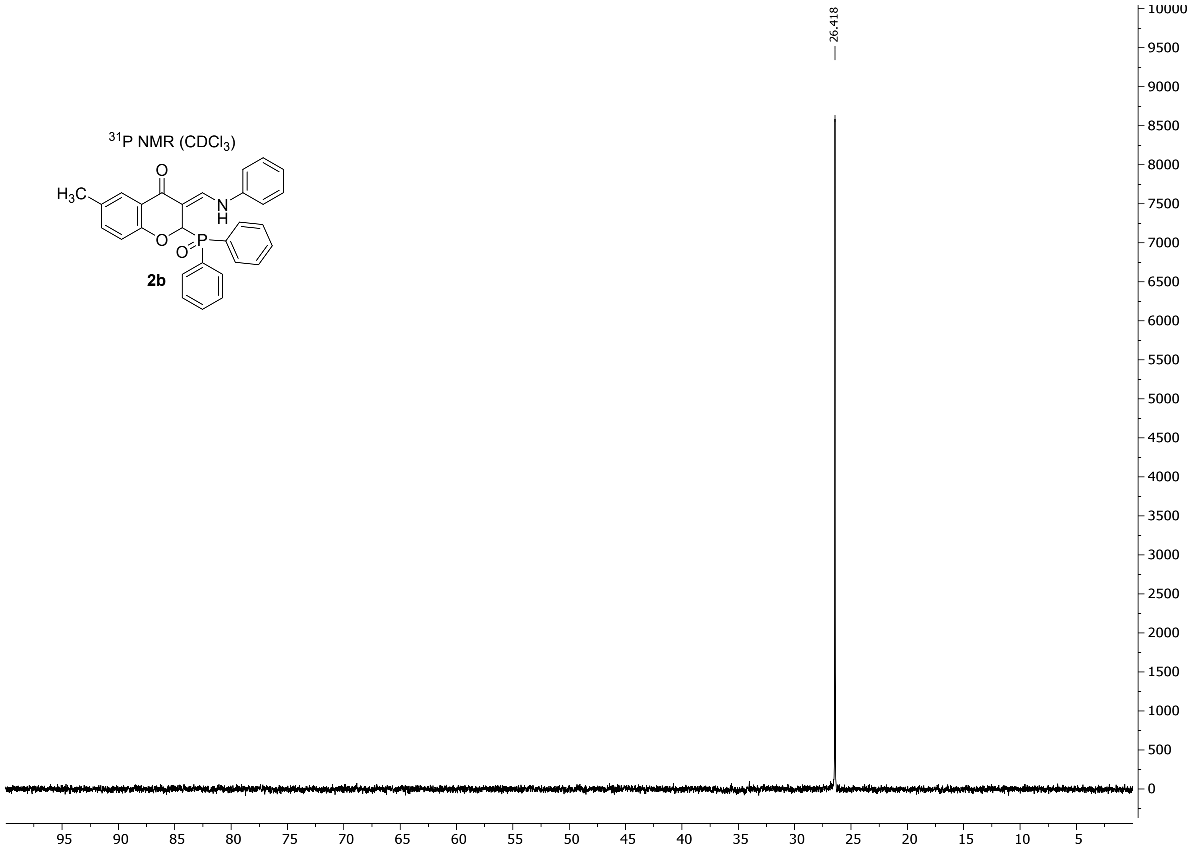

26.418
31P NMR (CDCl3)
2b

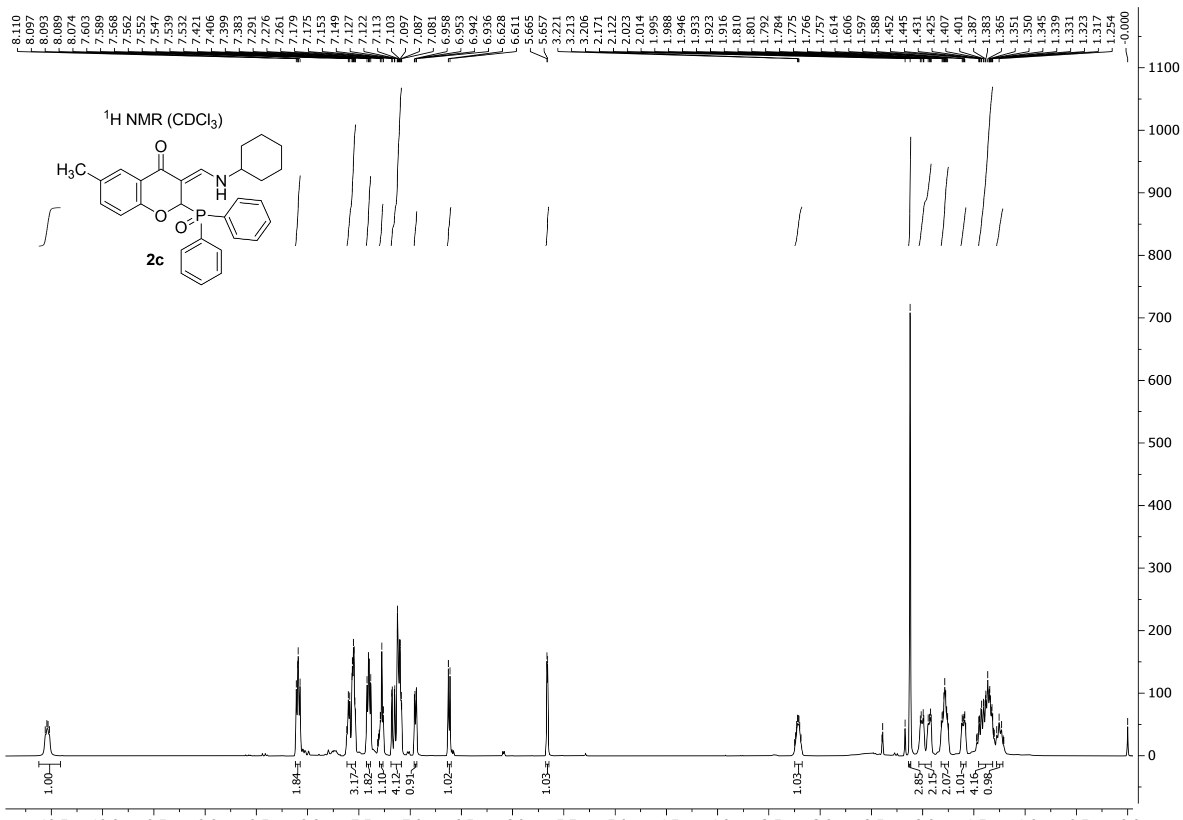

8.110 8.097 8.093 8.089 8.074 7.603 7.589 7.568 7.562 7.552 7.547 7.539 7.532 7.421 7.406 7.399 7.383 7.291 7.276 7.261 7.179 7.175 7.153 7.149 7.127 7.122 7.113 7.103 7.097 7.087 7.081 6.958 6.953 6.942 6.936 6.628 6.611 5.665 5.657 3.221 3.213 3.206 2.171 2.122 2.023 2.014 1.995 1.988 1.946 1.933 1.923 1.916 1.810 1.801 1.792 1.784 1.775 1.766 1.757 1.614 1.606 1.597 1.588 1.452 1.445 1.431 1.425 1.407 1.401 1.387 1.383 1.365 1.351 1.350 1.345 1.339 1.331 1.323 1.317 1.254 -0.000
1H NMR (CDCl3)
2c
f1 (ppm)

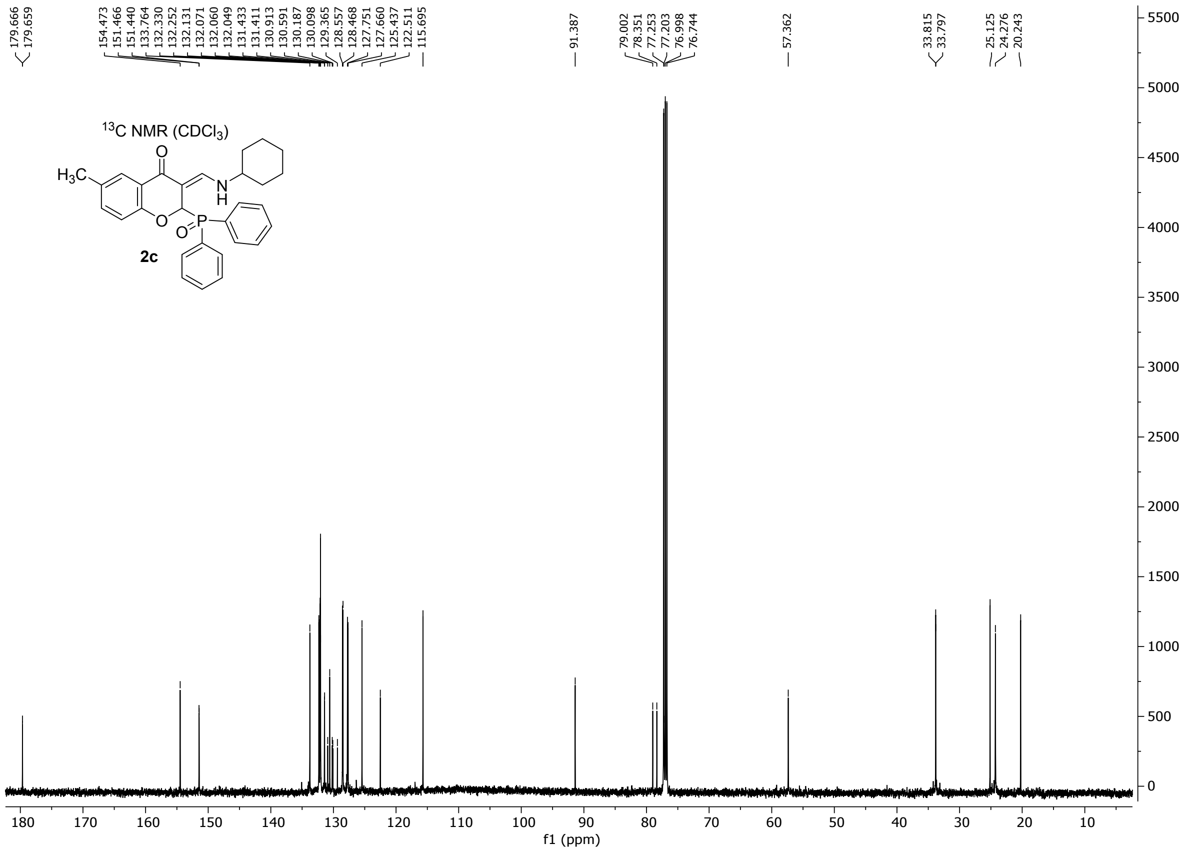

179.666
179.659
154.473
151.466
151.440
133.764
132.330
132.252
132.131
132.071
132.060
132.049
131.433
131.411
130.913
130.591
130.187
130.098
129.365
128.557
128.468
127.751
127.660
125.437
122.511
115.695
91.387
79.002
78.351
77.253
77.203
76.998
76.744
57.362
33.815
33.797
25.125
24.276
20.243
^{13}C NMR (CDCl$_3$)
H_3C
O
N
H
O
P
O
2c
f1 (ppm)

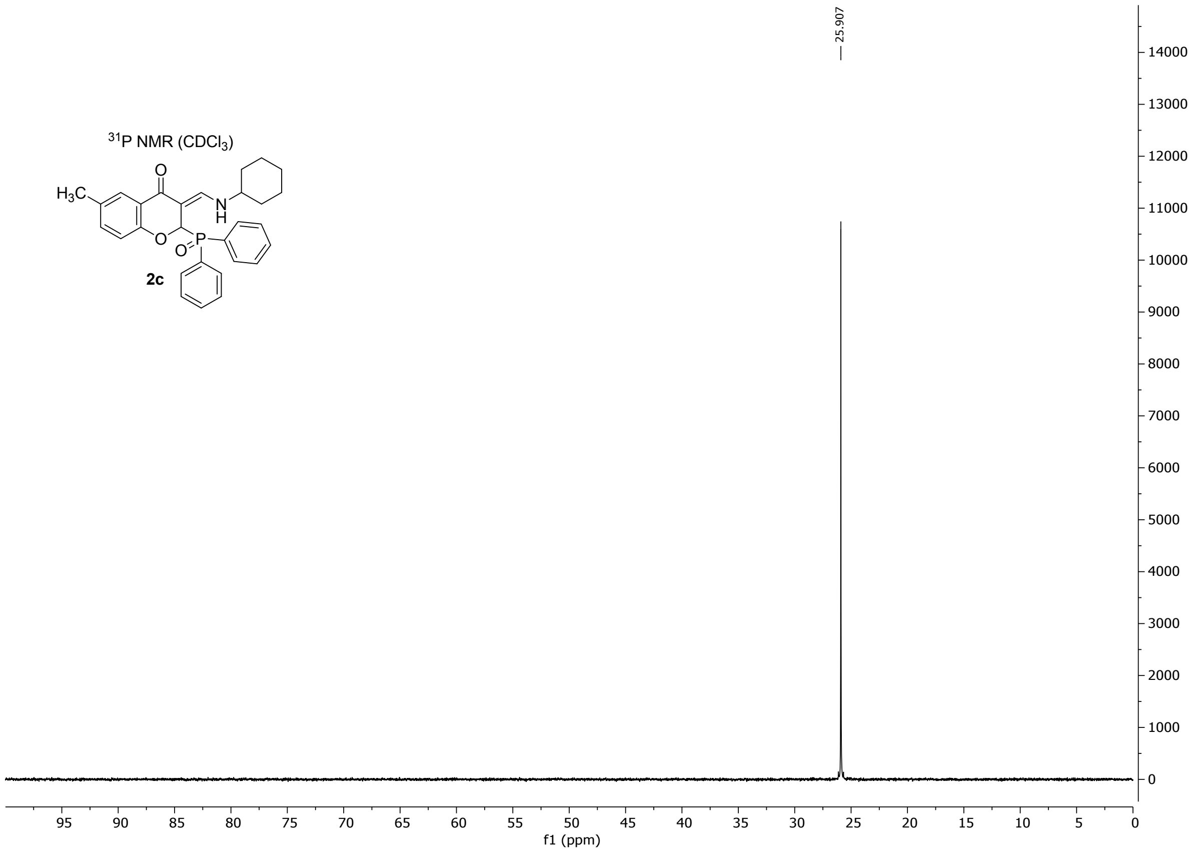

25.907
^{31}P NMR (CDCl$_3$)
H_3C
O
N
H
O
P
O
2c
f1 (ppm)

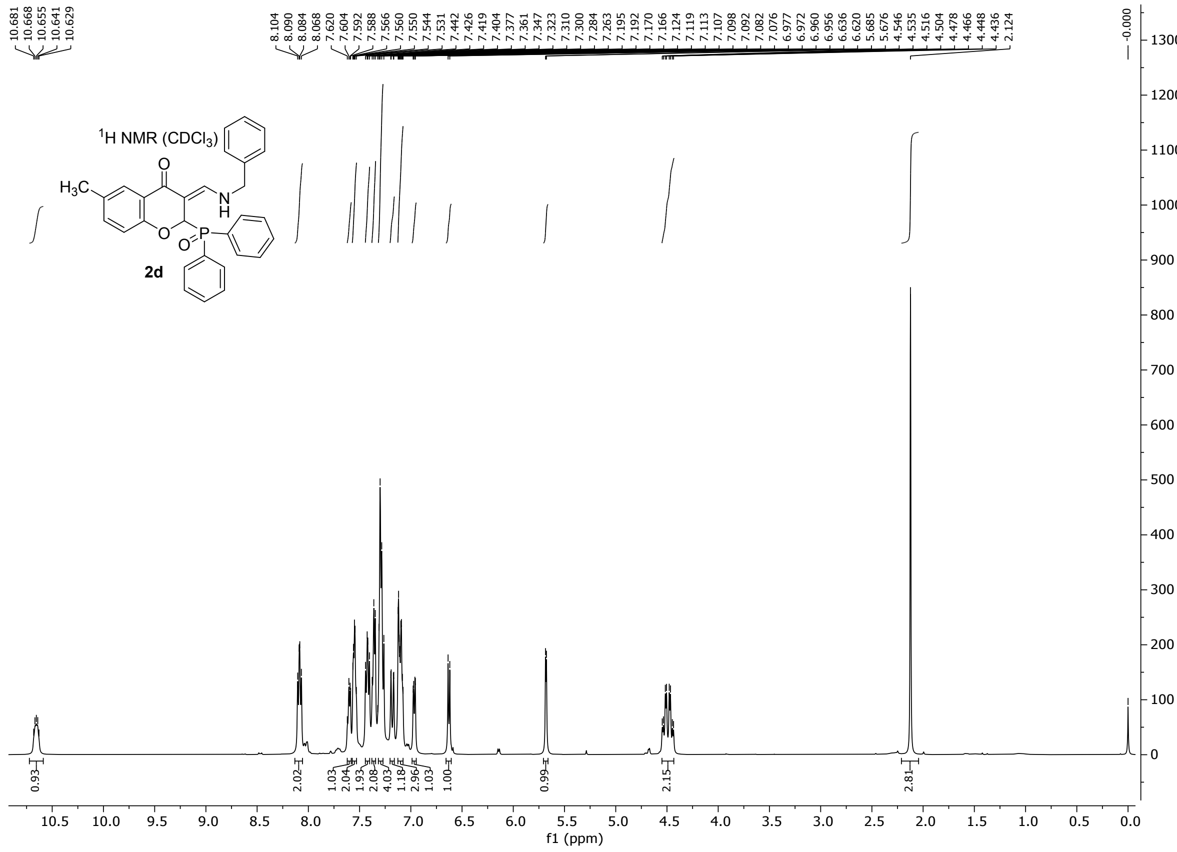
1H NMR (CDCl3)
2d
f1 (ppm)

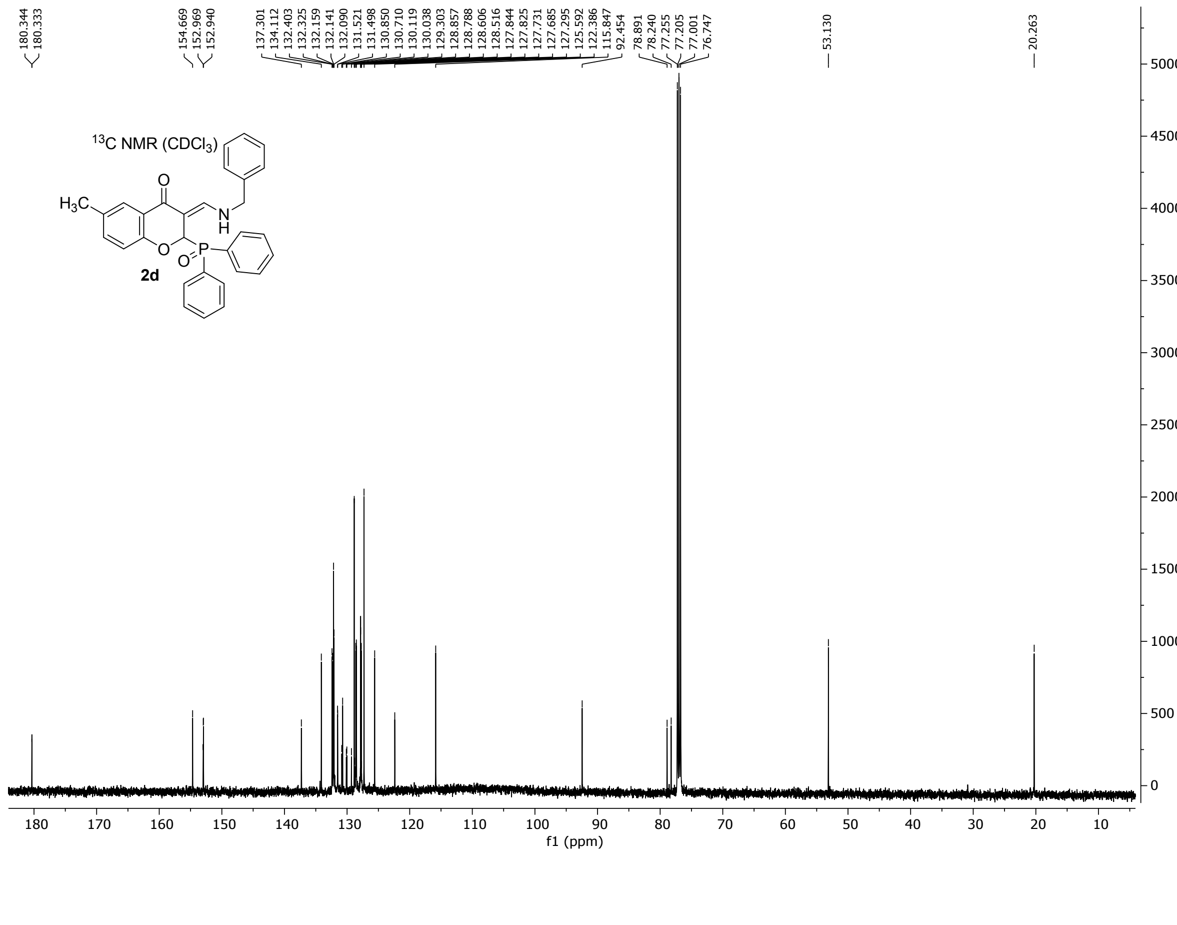
13C NMR (CDCl3)
2d
f1 (ppm)

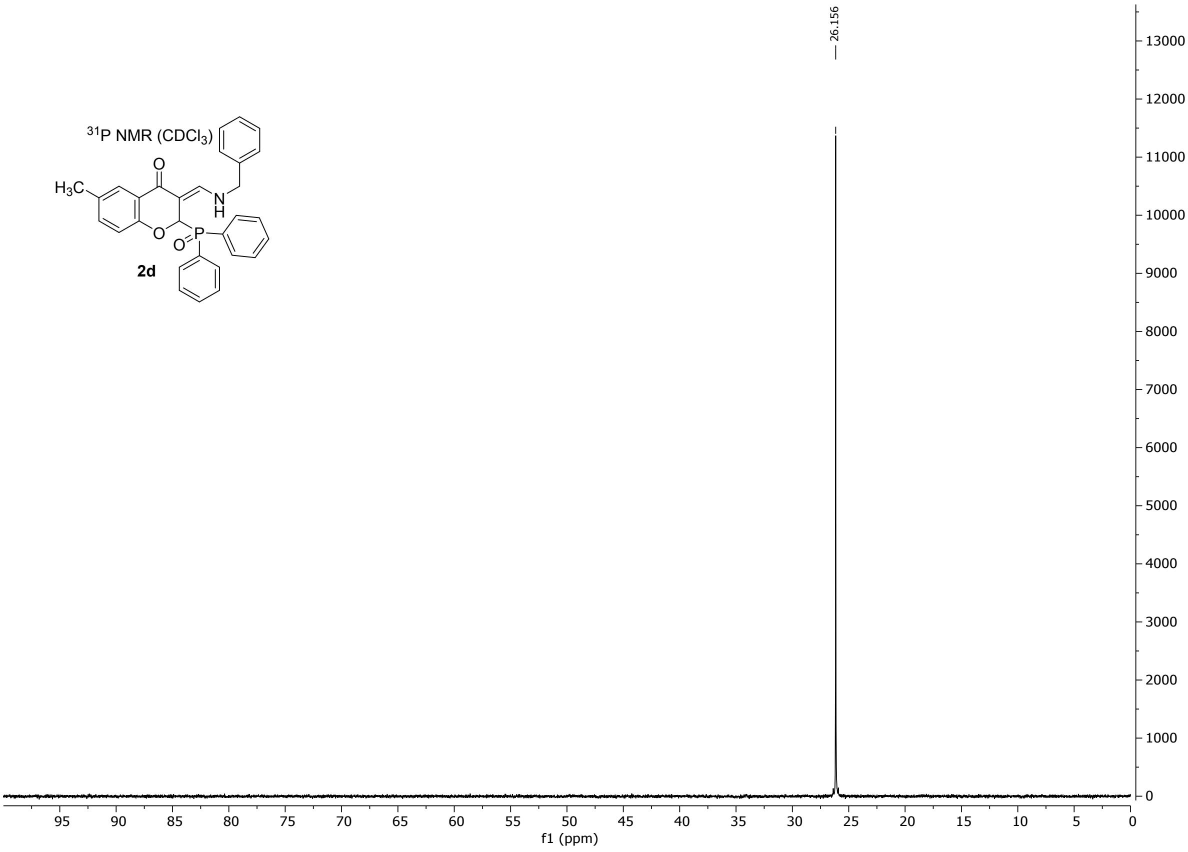

26.156
^{31}P NMR (CDCl$_3$)
2d
f1 (ppm)

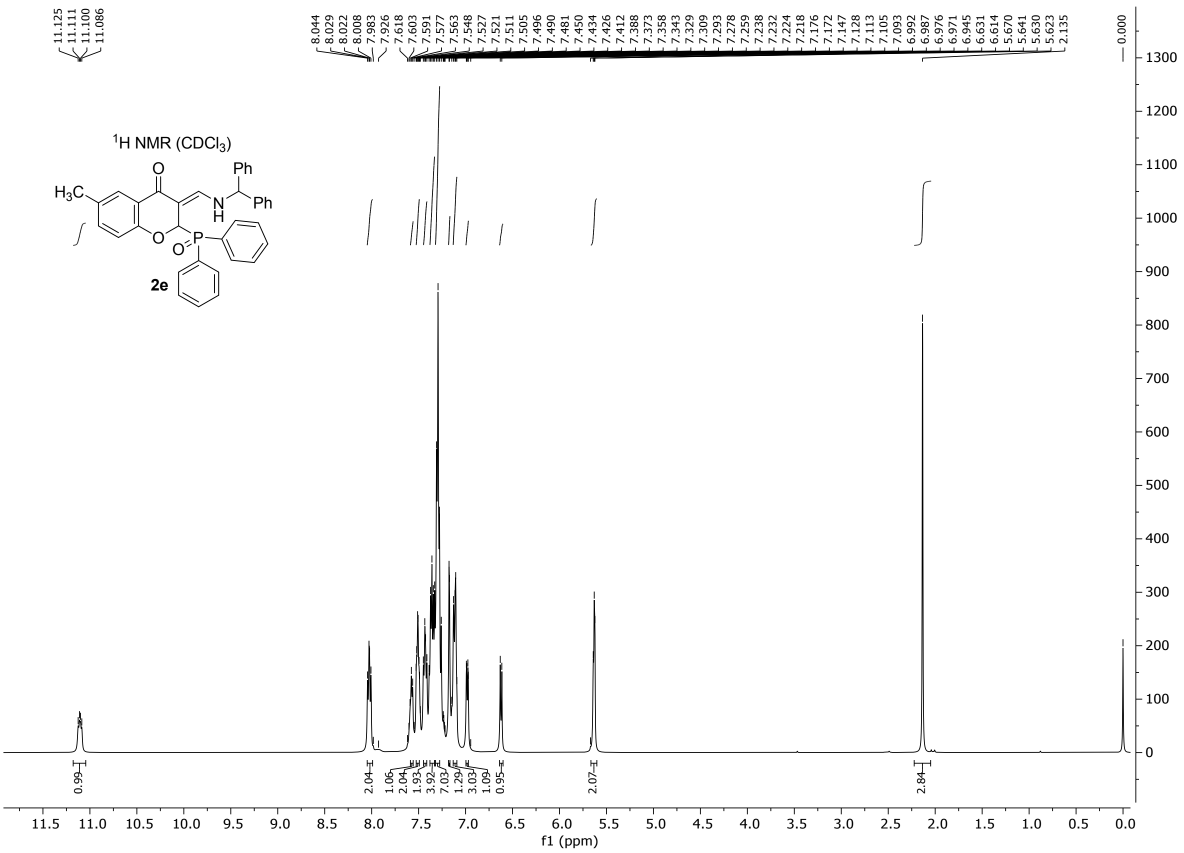

^{1}H NMR (CDCl$_3$)
2e
f1 (ppm)

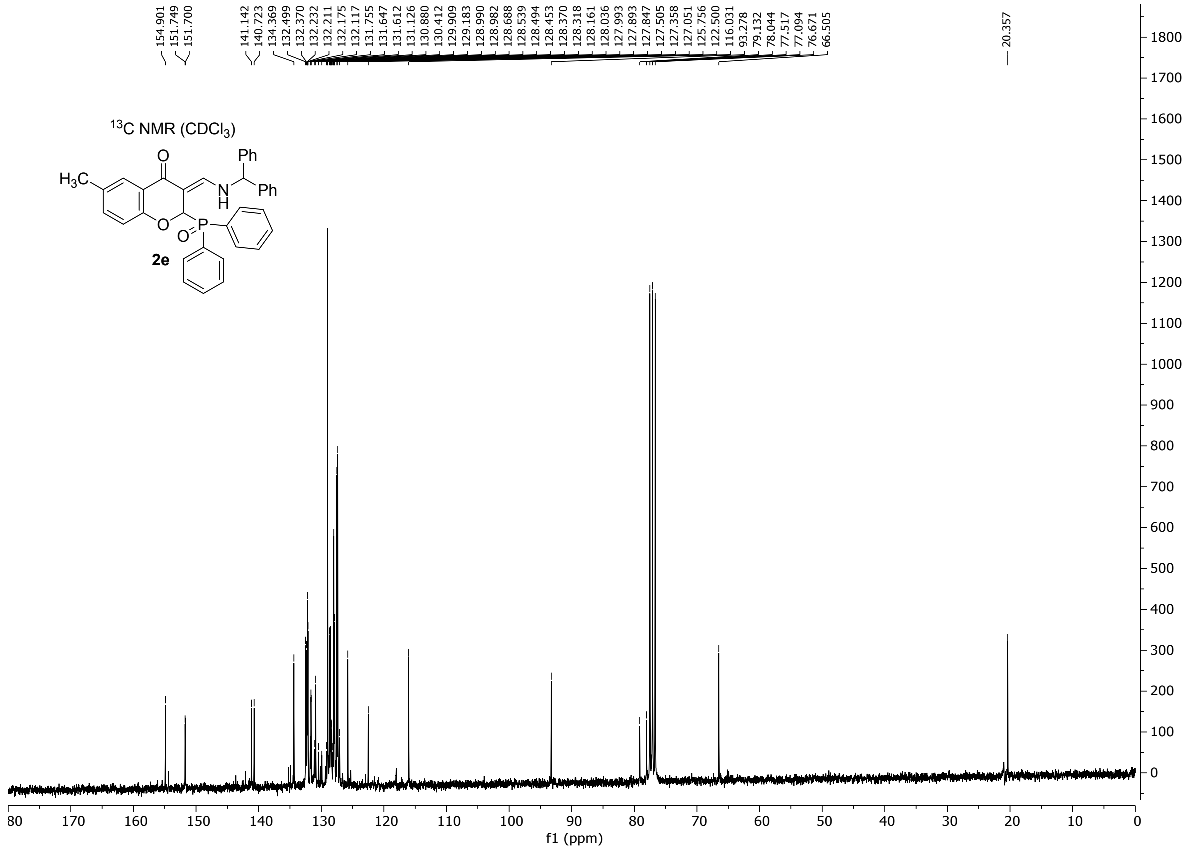
13C NMR (CDCl3)
2e
154.901
151.749
151.700
141.142
140.723
134.369
132.499
132.370
132.232
132.211
132.175
132.117
131.755
131.647
131.612
131.126
130.880
130.412
129.909
129.183
128.990
128.982
128.688
128.539
128.494
128.453
128.370
128.318
128.161
128.036
127.993
127.893
127.847
127.505
127.358
127.051
125.756
122.500
116.031
93.278
79.132
78.044
77.517
77.094
76.671
66.505
20.357
f1 (ppm)

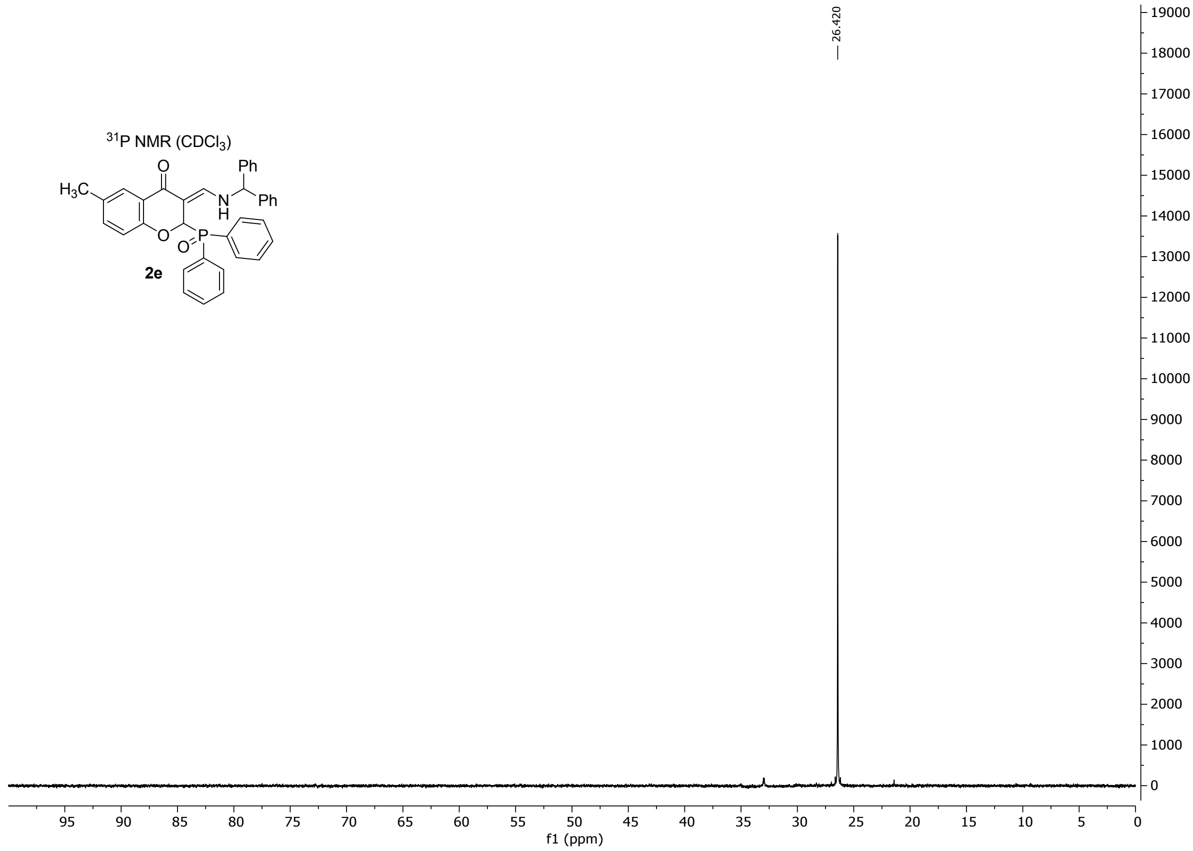
31P NMR (CDCl3)
2e
26.420
f1 (ppm)

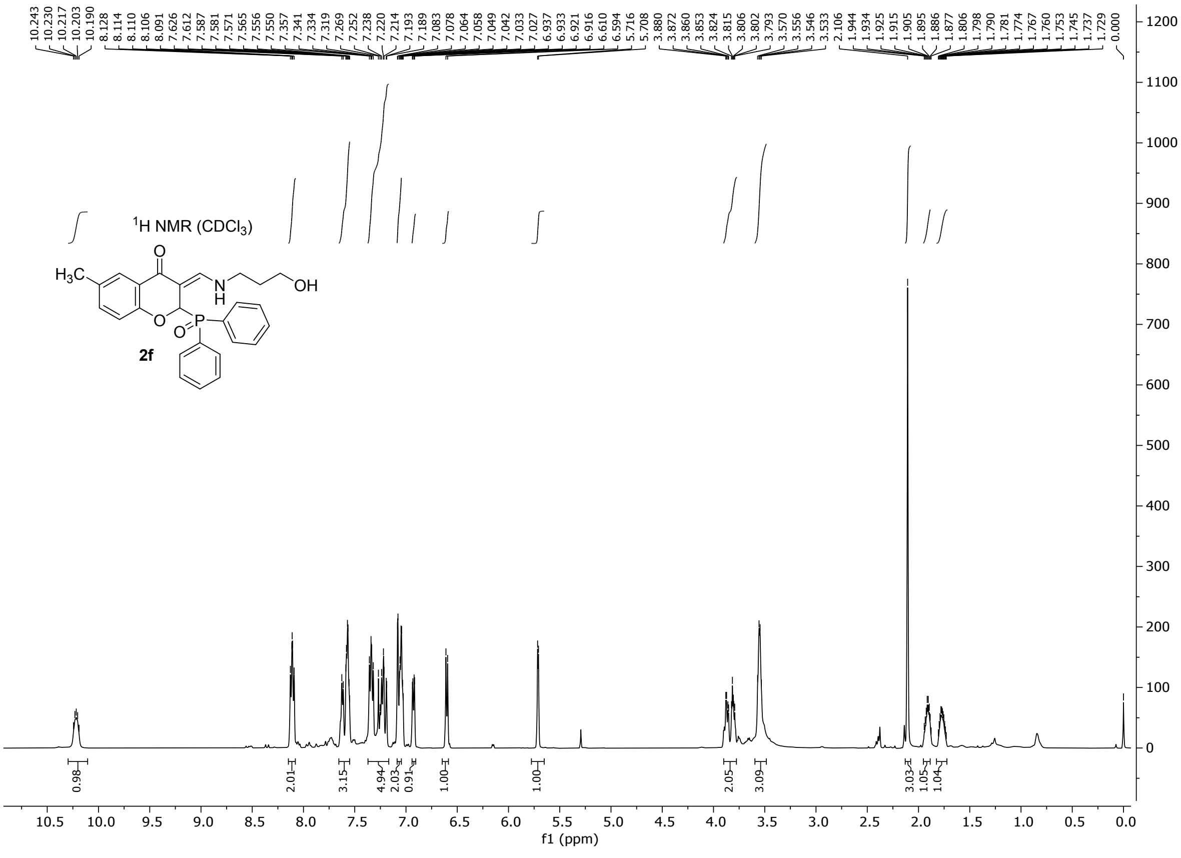

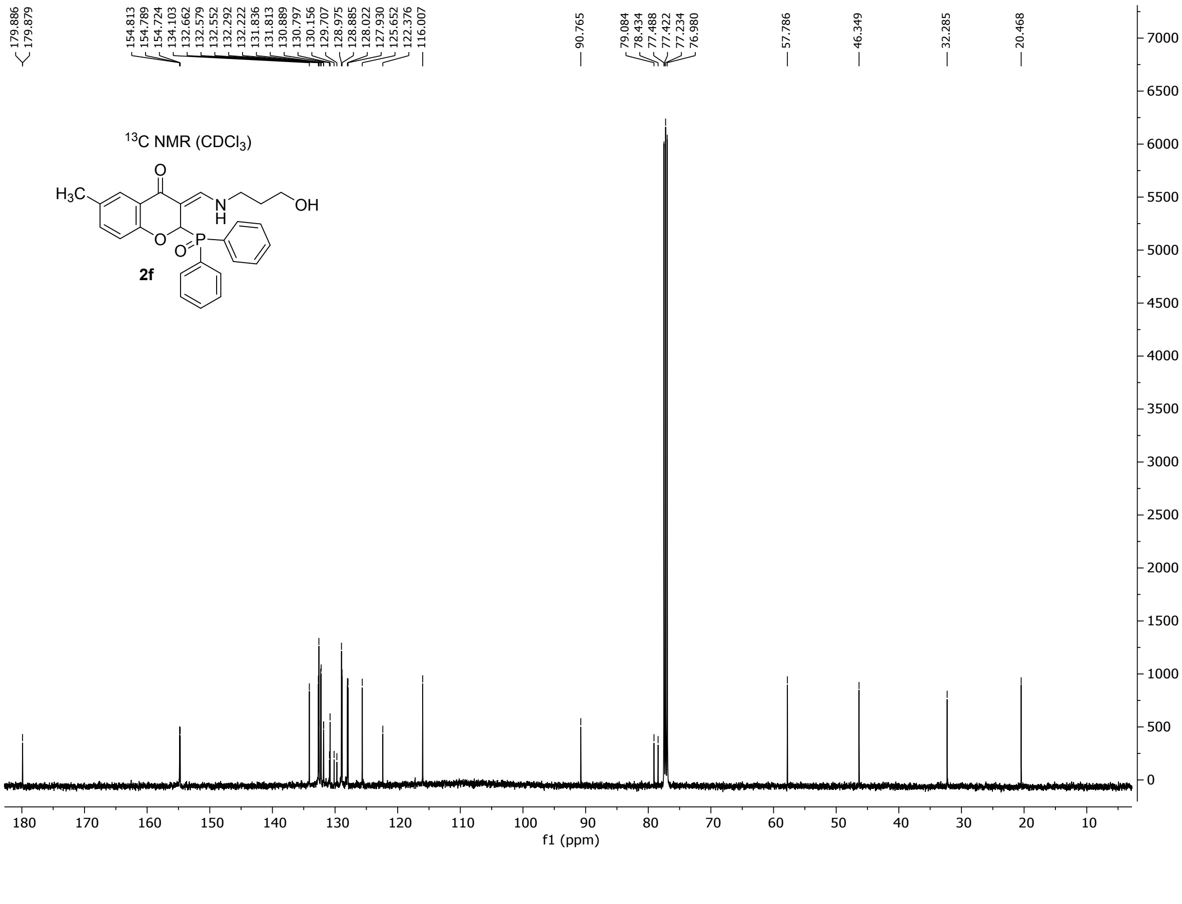

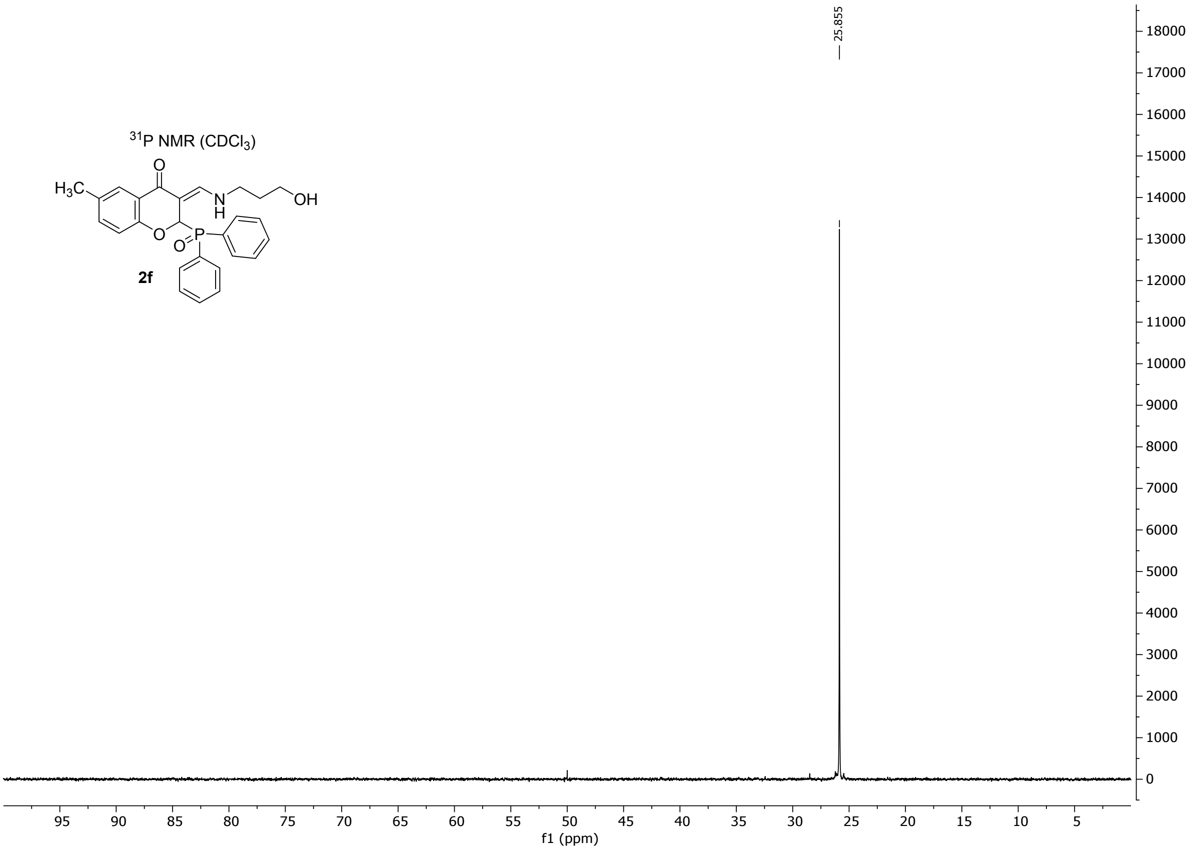

31P NMR (CDCl3)
H3C
O
N
H
OH
O
P
O
2f
25.855
f1 (ppm)

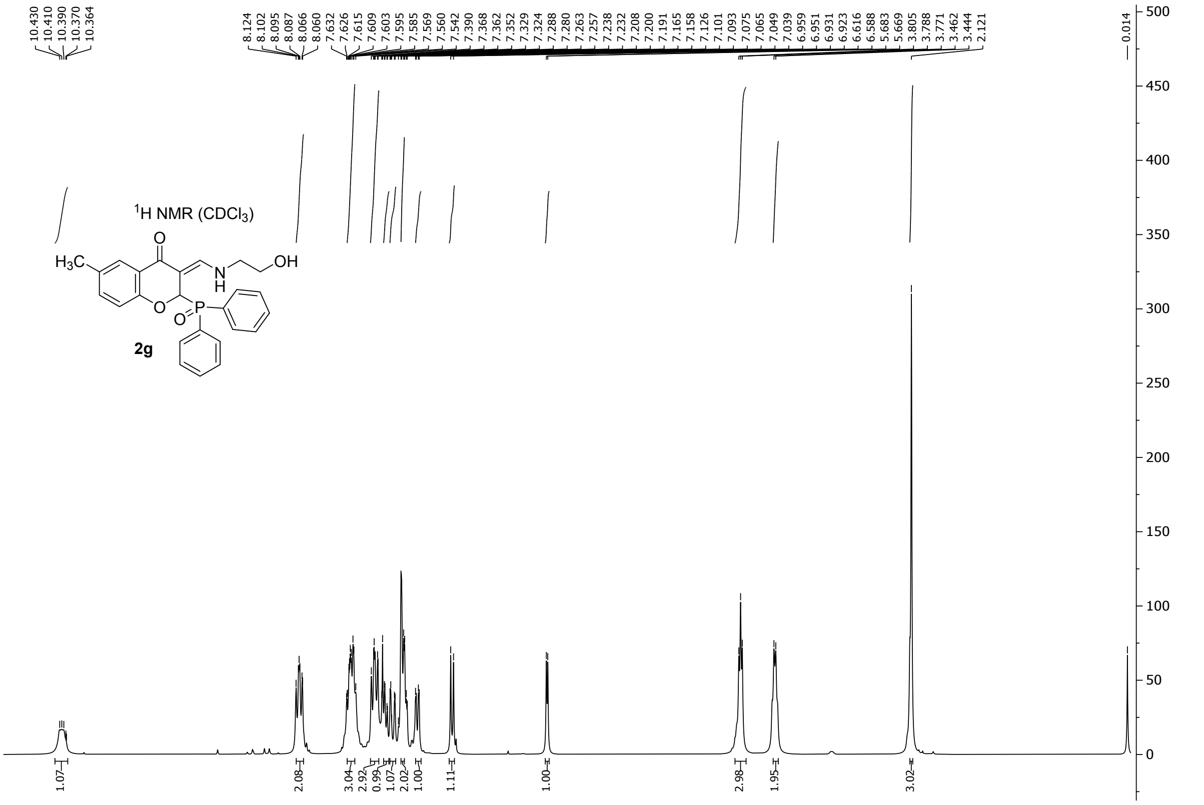

1H NMR (CDCl3)
H3C
O
N
H
OH
O
P
O
2g
10.430 10.410 10.390 10.370 10.364
8.124 8.102 8.095 8.087 8.066 8.060 7.632 7.626 7.615 7.609 7.603 7.595 7.585 7.569 7.560 7.542 7.390 7.368 7.362 7.352 7.329 7.324 7.288 7.280 7.263 7.257 7.238 7.232 7.208 7.200 7.191 7.165 7.158 7.126 7.101 7.093 7.075 7.065 7.049 7.039 6.959 6.951 6.931 6.923 6.616 6.588 5.683 5.669 3.805 3.788 3.771 3.462 3.444 2.121
0.014
1.07
2.08
3.04
2.92
0.99
1.07
2.02
1.00
1.11
1.00
2.98
1.95
3.02
f1 (ppm)

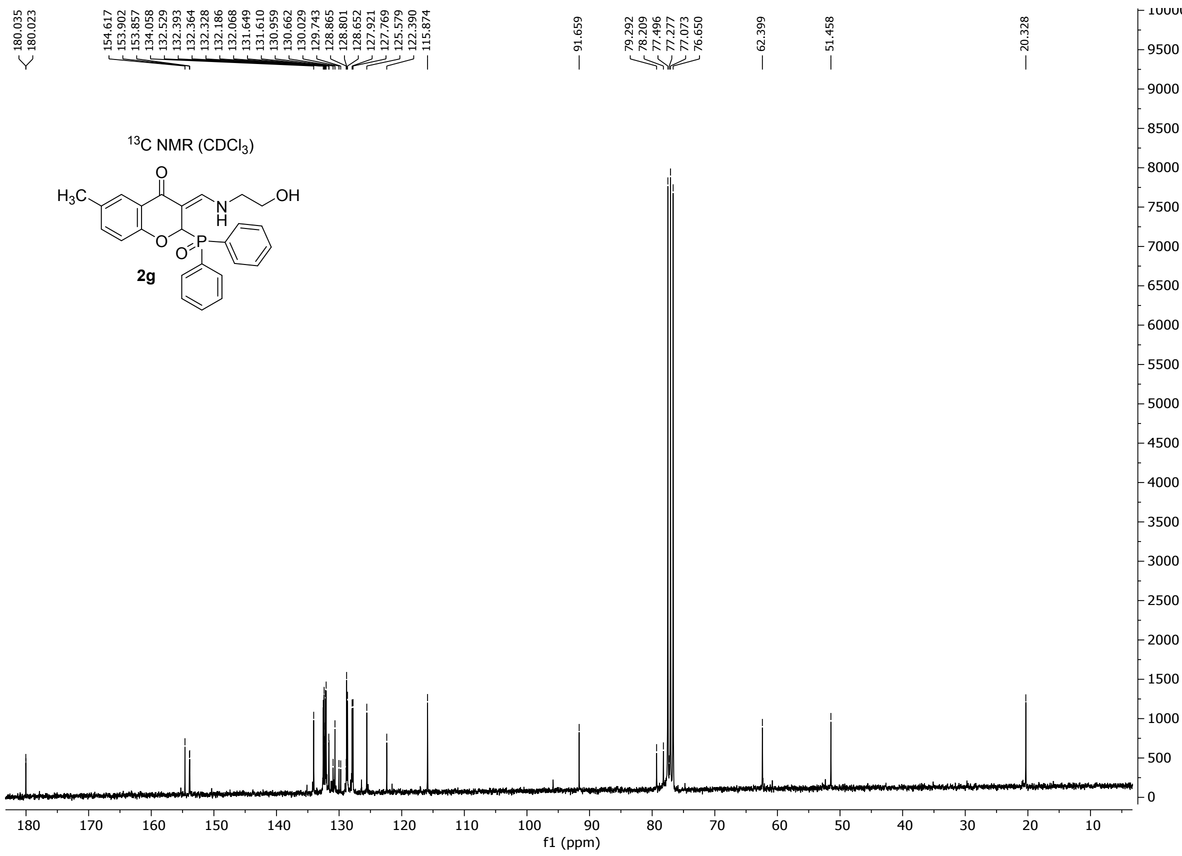

180.035
180.023
154.617
153.902
153.857
134.058
132.529
132.393
132.364
132.328
132.186
132.068
131.649
131.610
130.959
130.662
130.029
129.743
128.865
128.801
128.652
127.921
127.769
125.579
122.390
115.874
91.659
79.292
78.209
77.496
77.277
77.073
76.650
62.399
51.458
20.328
^{13}C NMR (CDCl$_3$)
2g
f1 (ppm)

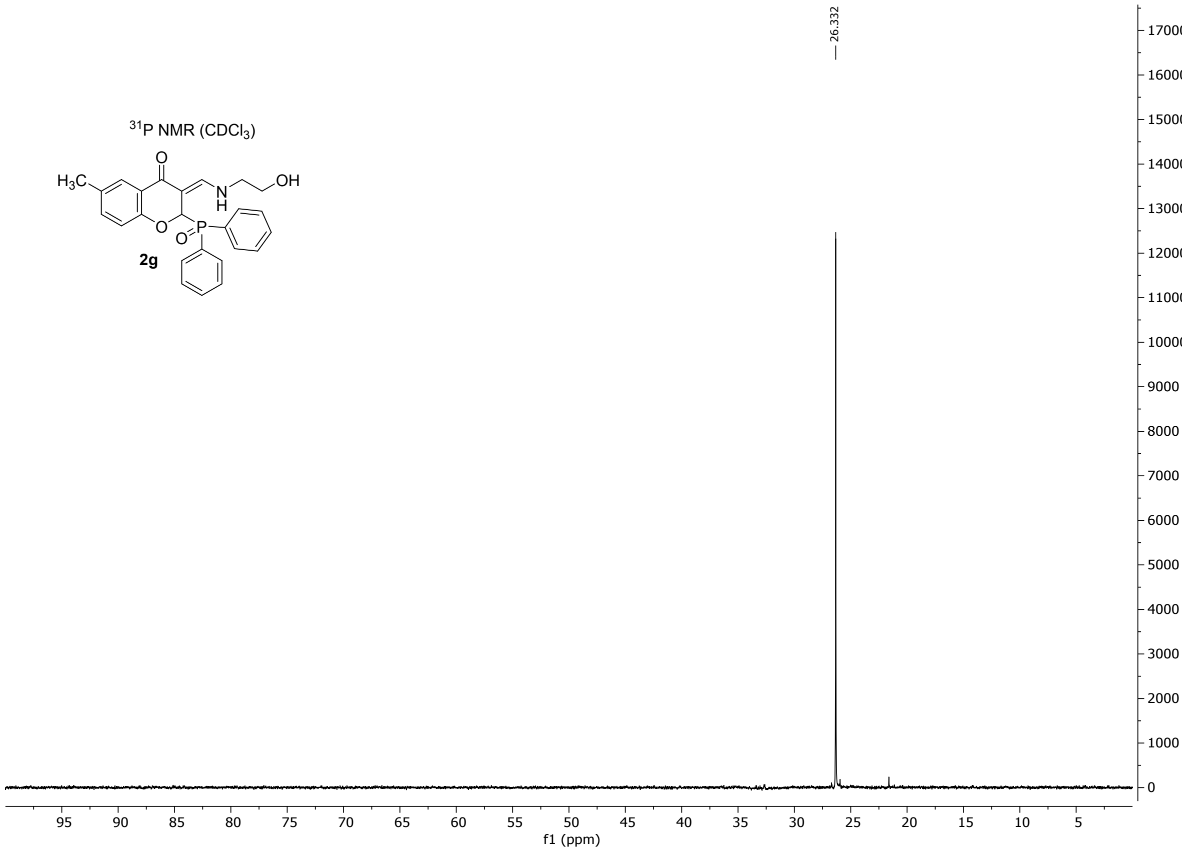

26.332
^{31}P NMR (CDCl$_3$)
2g
f1 (ppm)

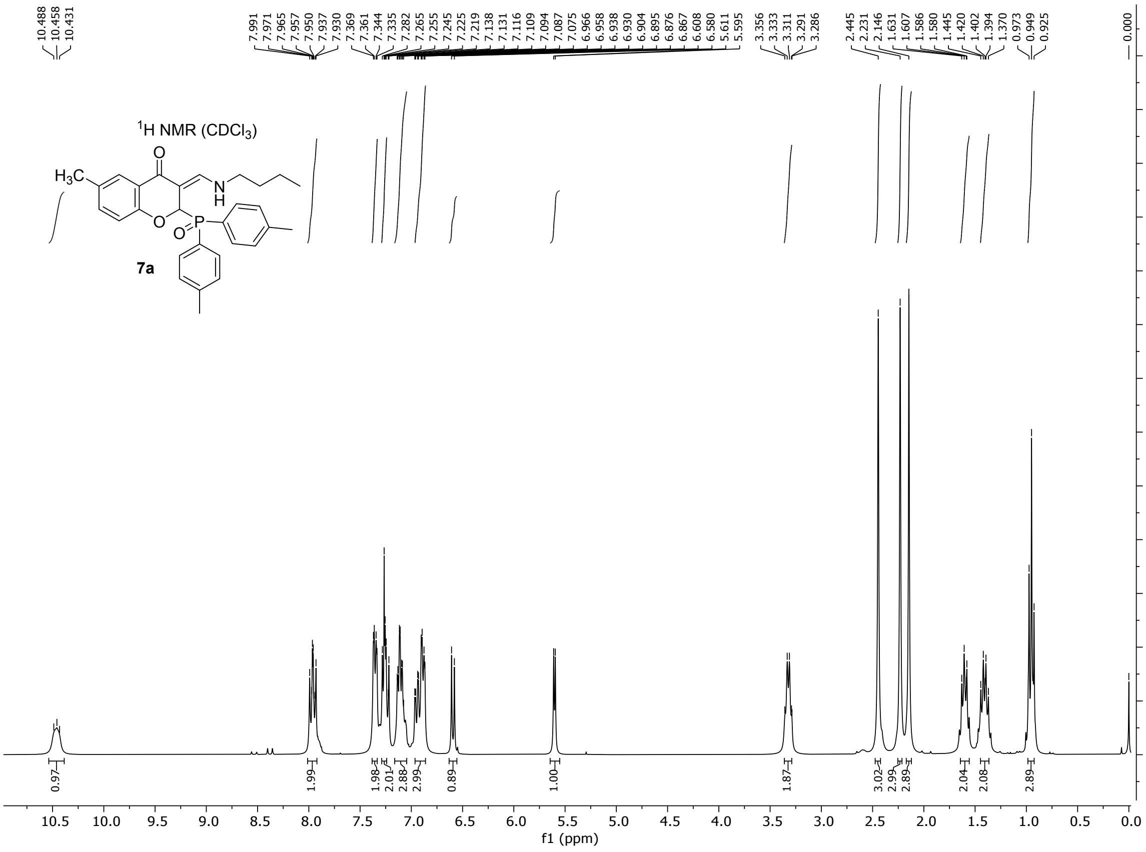

1H NMR (CDCl3)
7a
f1 (ppm)

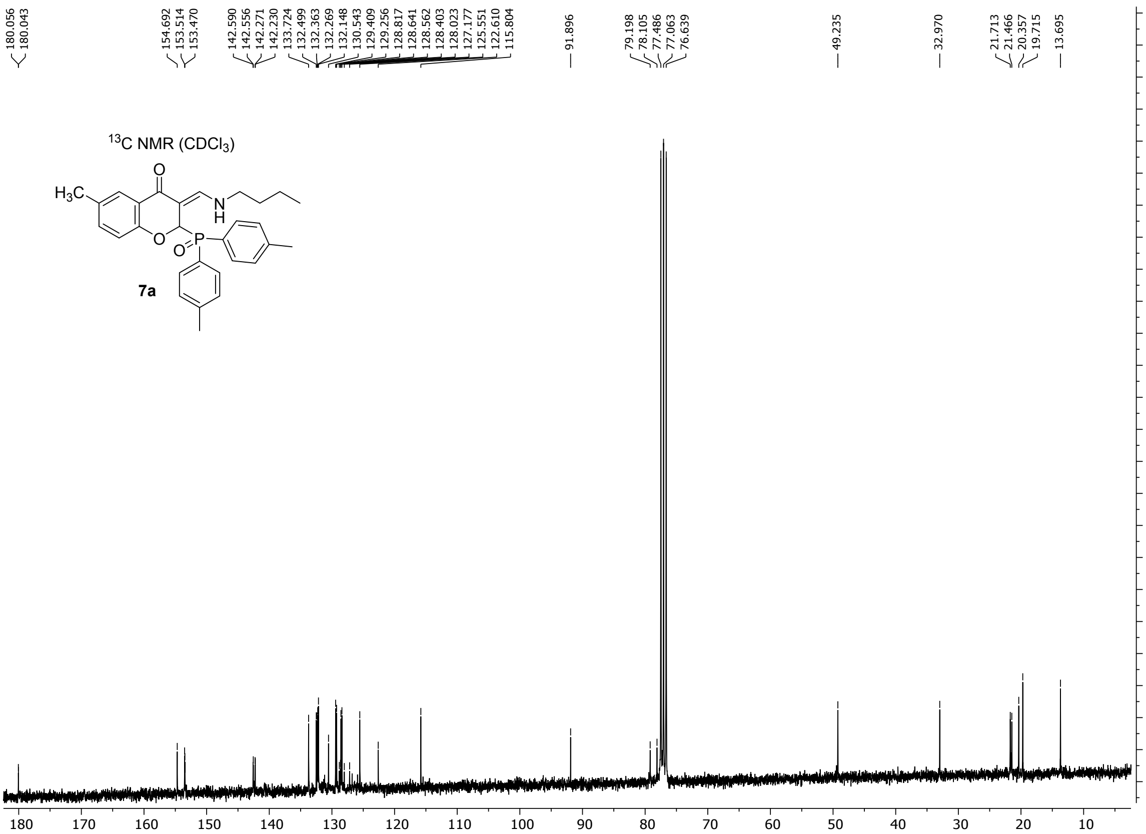

13C NMR (CDCl3)
7a
f1 (ppm)

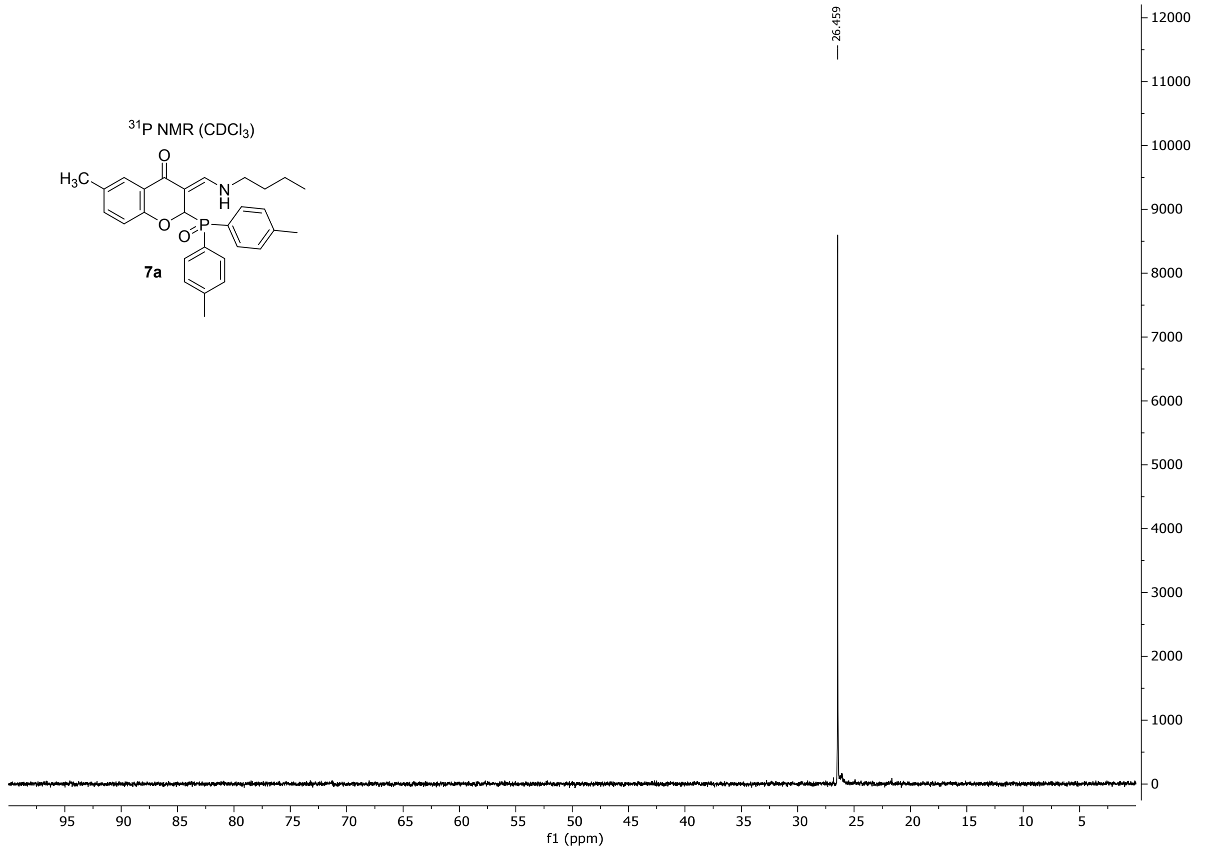

26.459
^{31}P NMR (CDCl$_3$)
7a
f1 (ppm)

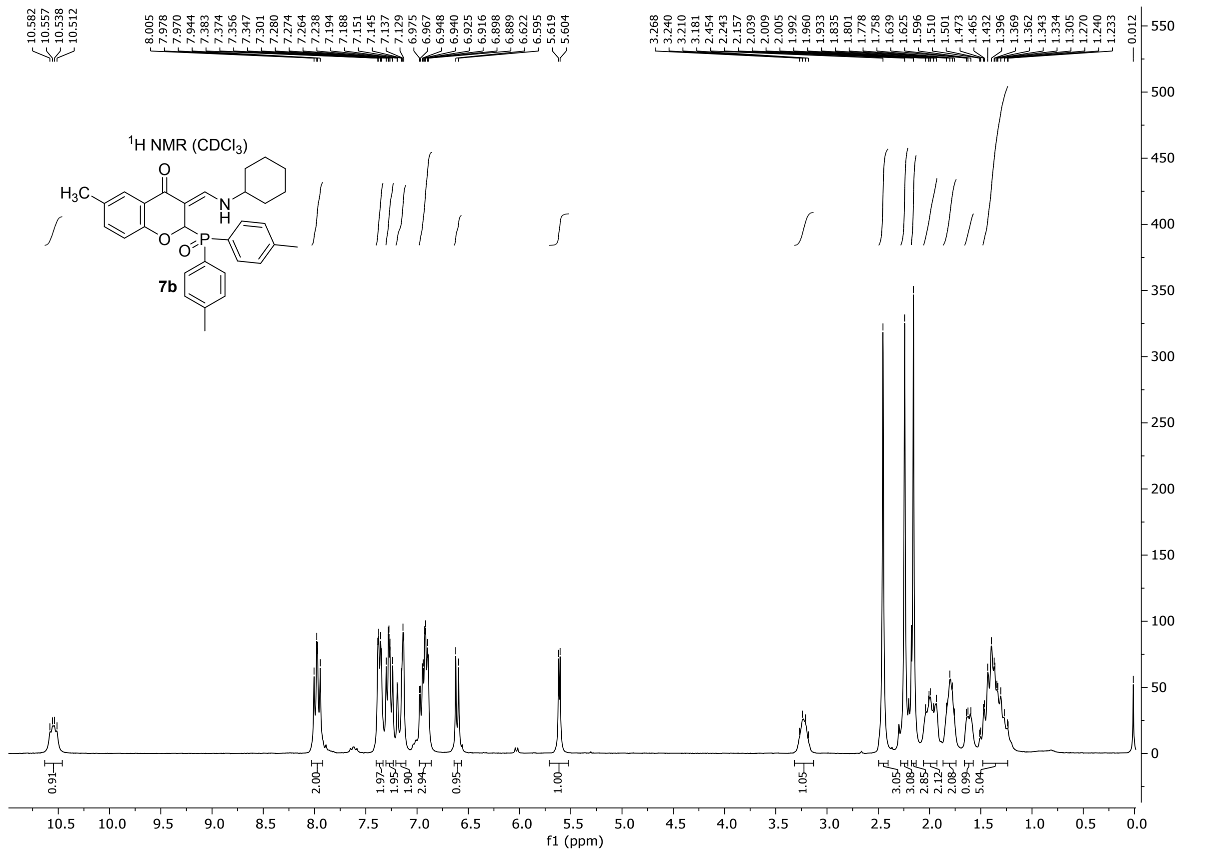

^{1}H NMR (CDCl$_3$)
7b
f1 (ppm)

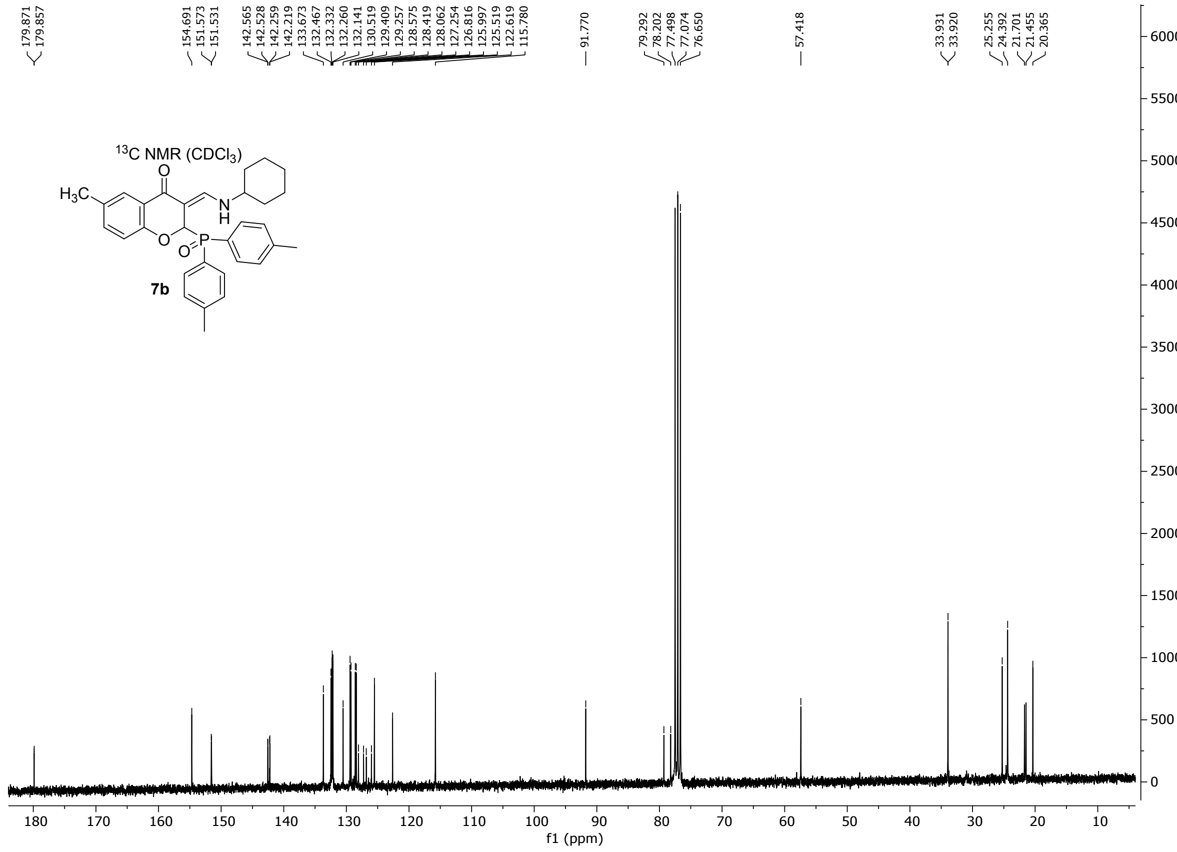

^{13}C NMR (CDCl$_3$)
7b
f1 (ppm)

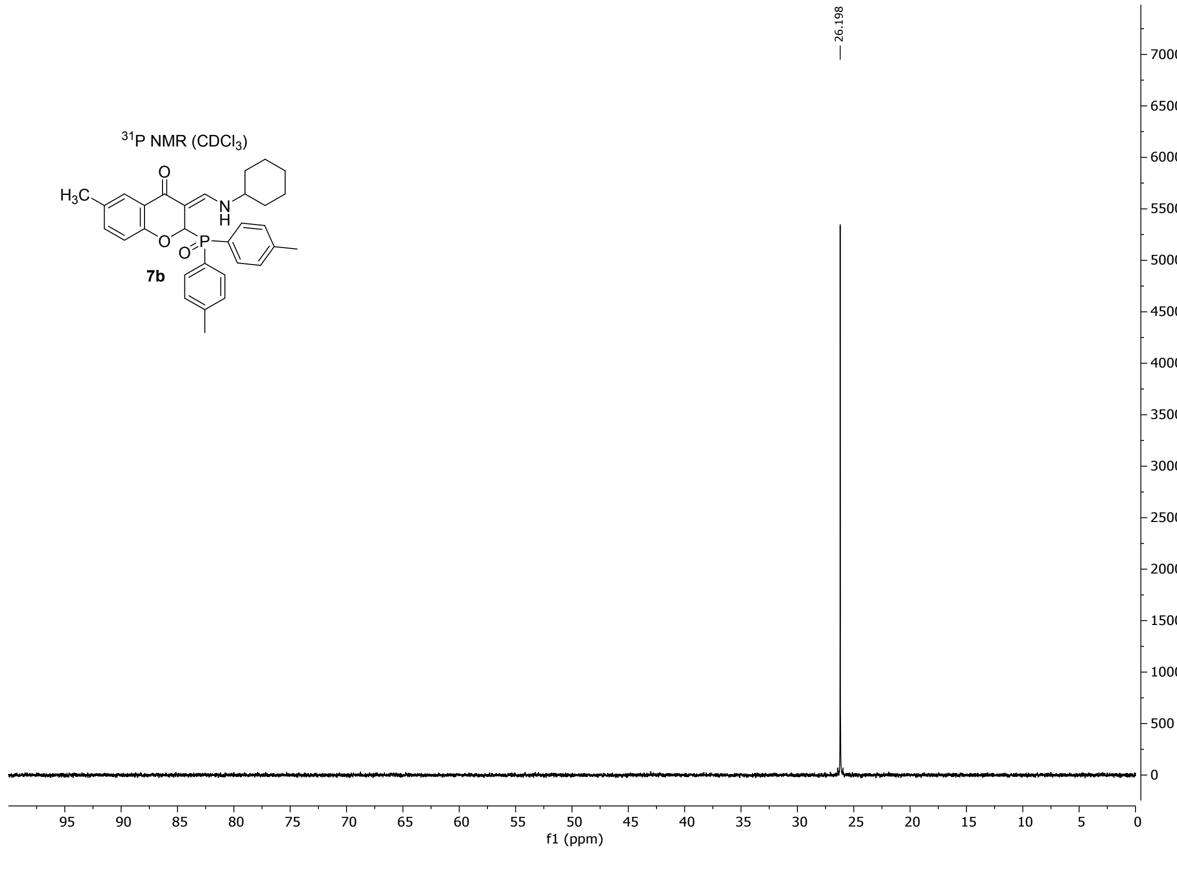

^{31}P NMR (CDCl$_3$)
7b
26.198
f1 (ppm)

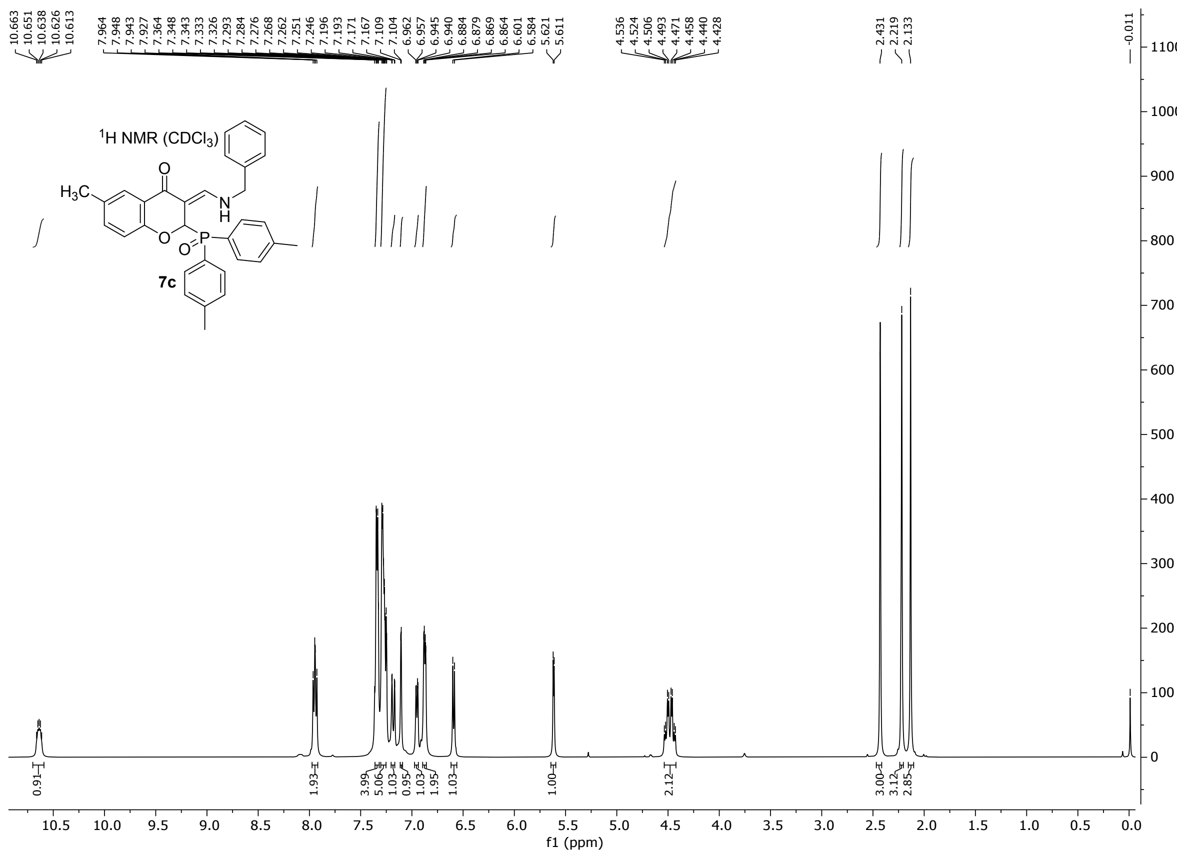

¹H NMR (CDCl₃)
7c
f1 (ppm)

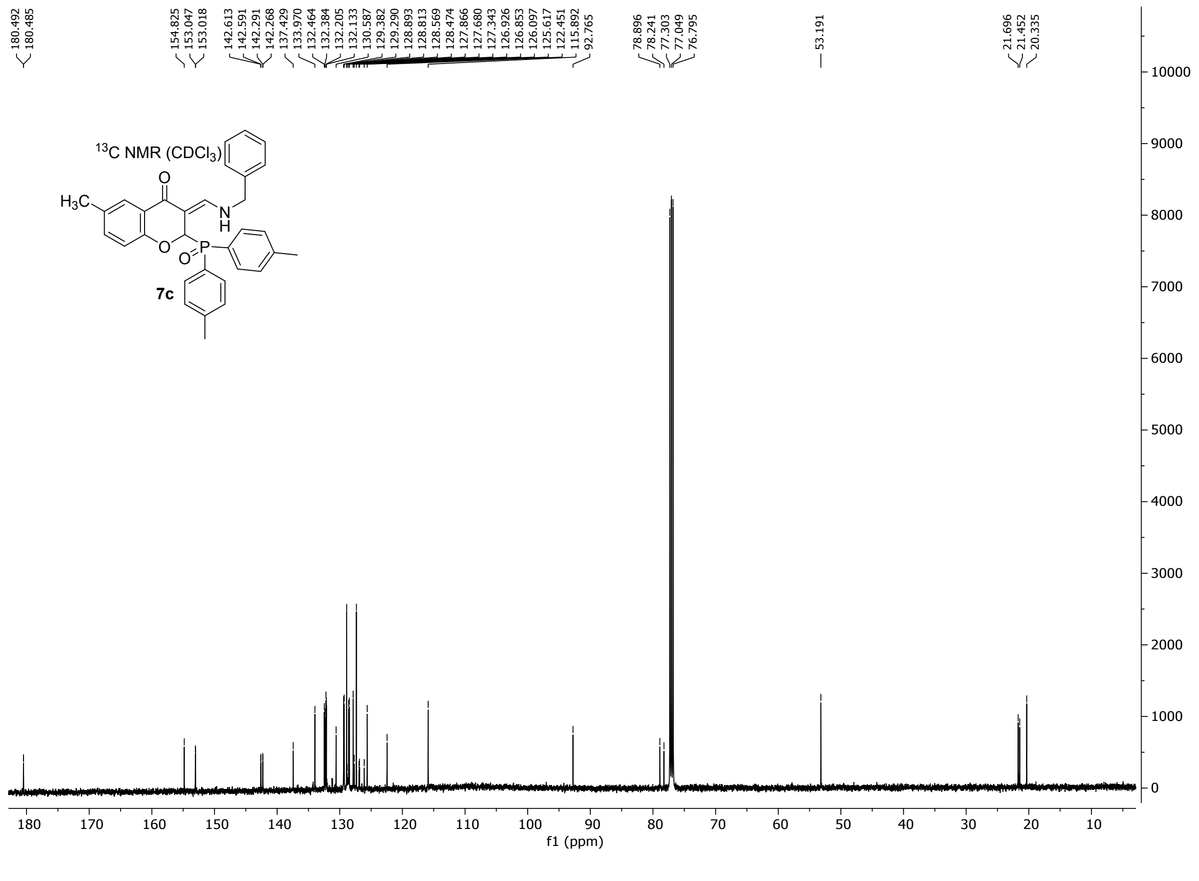

¹³C NMR (CDCl₃)
7c
f1 (ppm)

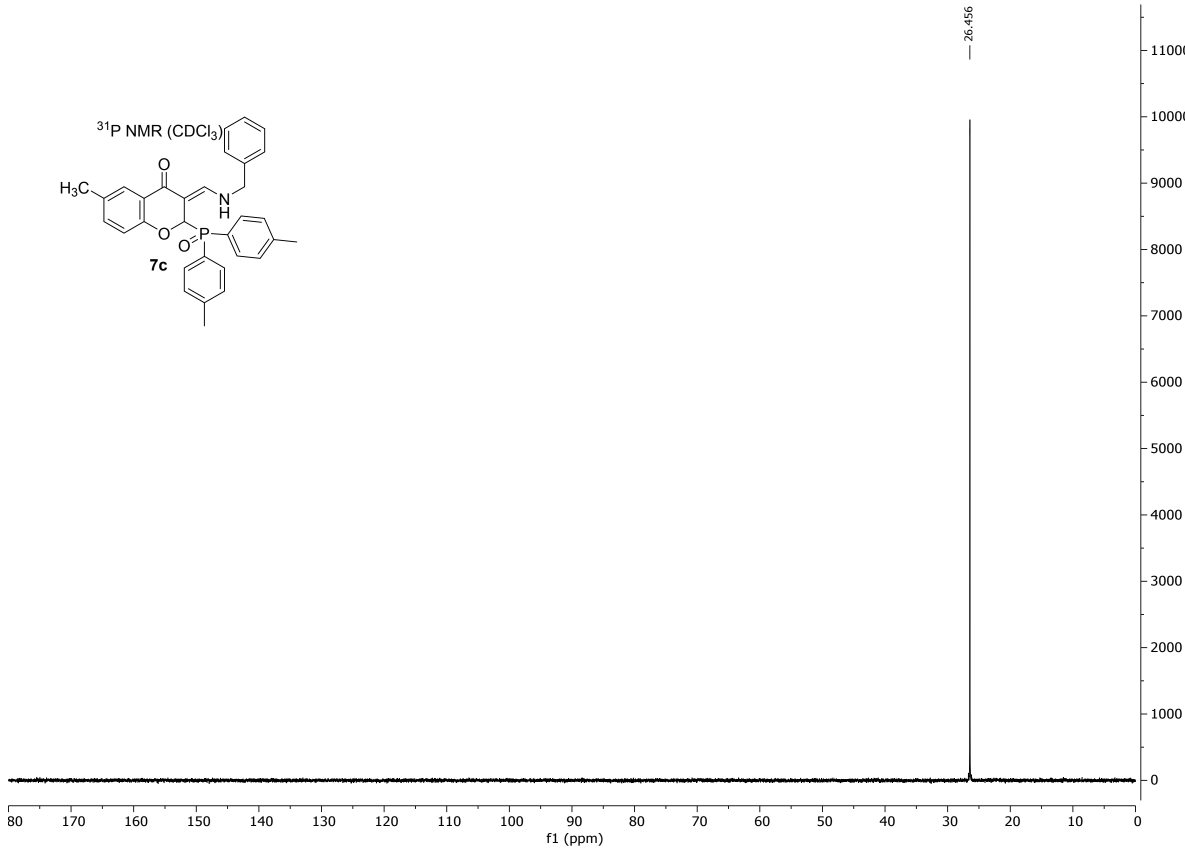
31P NMR (CDCl3)
7c
26.456
f1 (ppm)

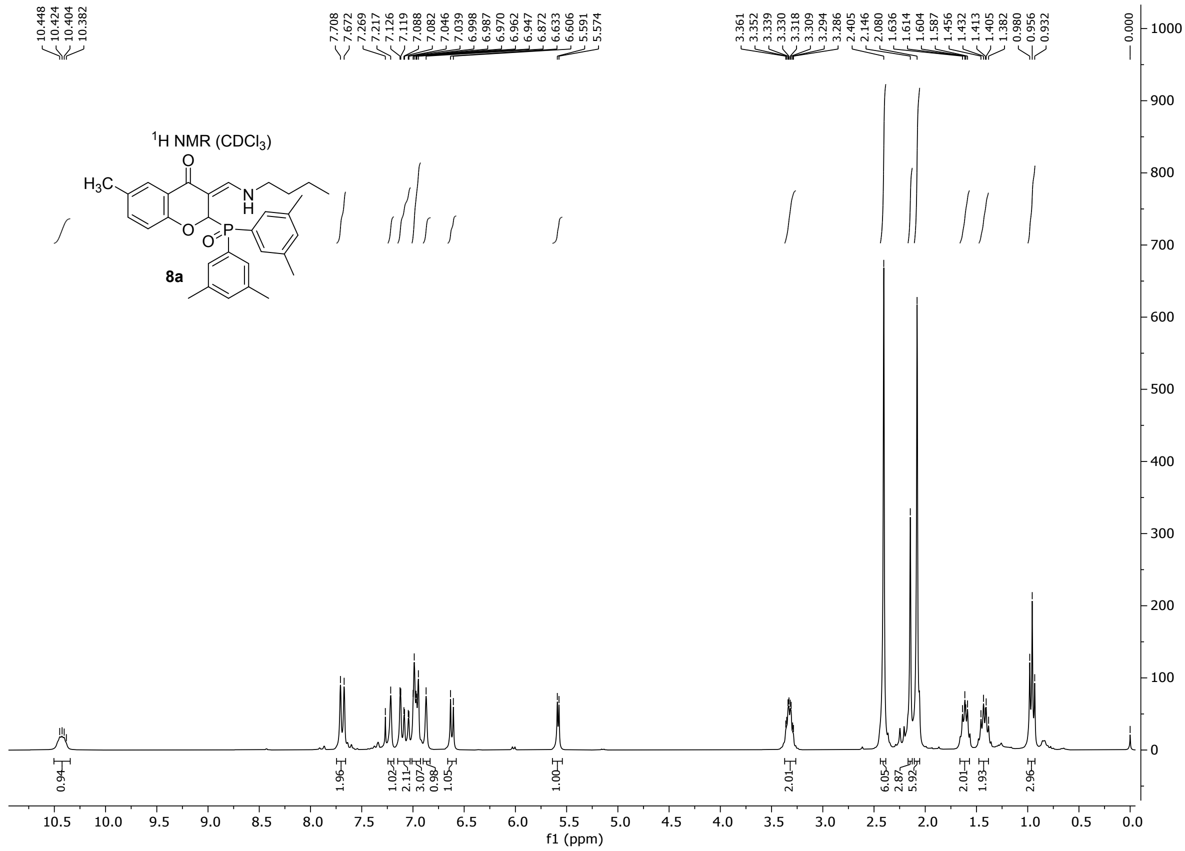
1H NMR (CDCl3)
8a
f1 (ppm)

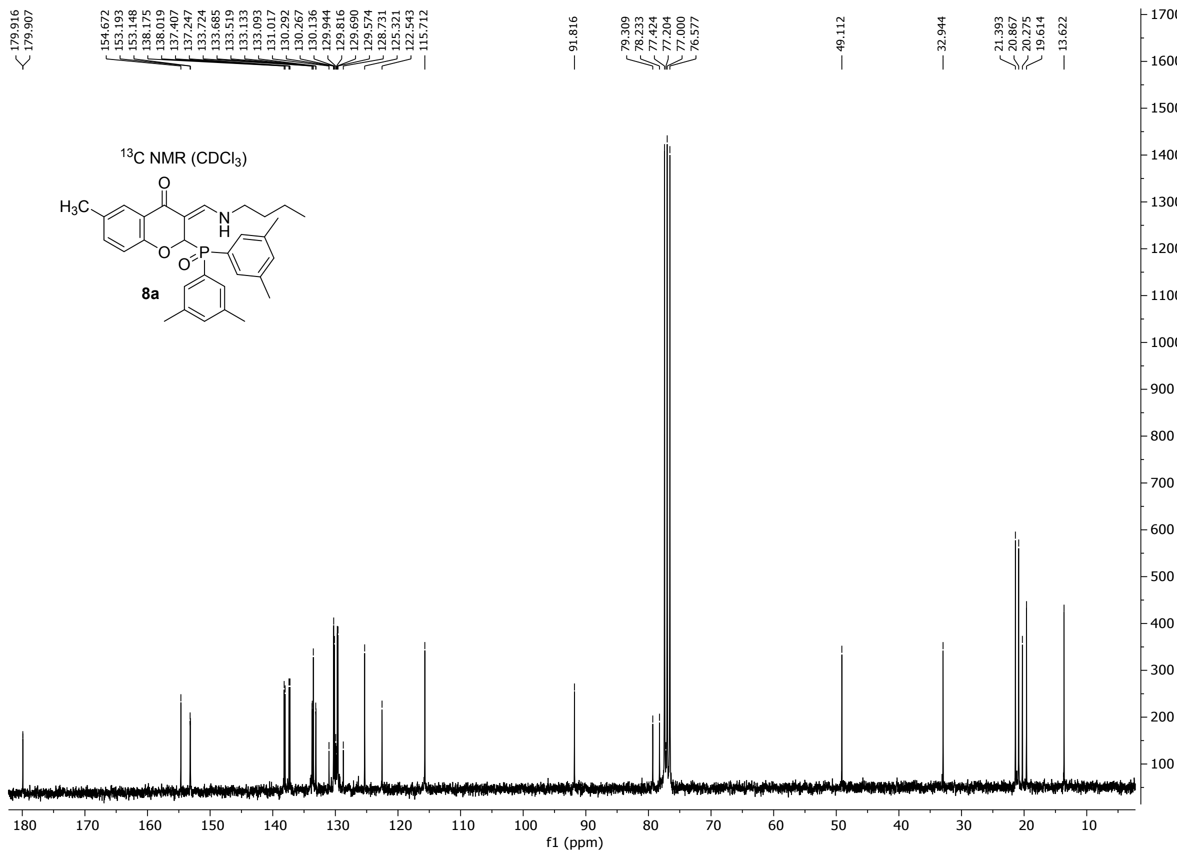
13C NMR (CDCl3)
8a
179.916
179.907
154.672
153.193
153.148
138.175
138.019
137.407
137.247
133.724
133.685
133.519
133.133
133.093
131.017
130.292
130.267
130.136
129.944
129.816
129.690
129.574
128.731
125.321
122.543
115.712
91.816
79.309
78.233
77.424
77.204
77.000
76.577
49.112
32.944
21.393
20.867
20.275
19.614
13.622
f1 (ppm)

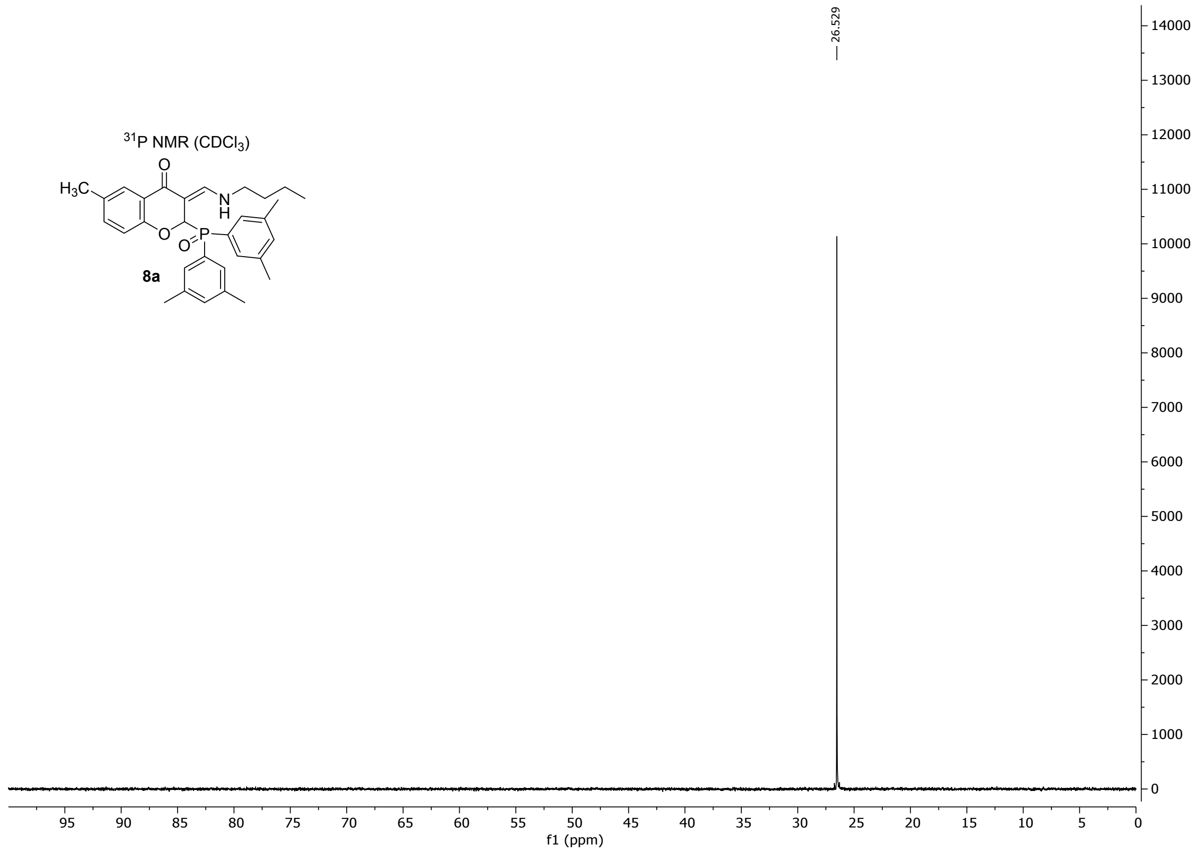
31P NMR (CDCl3)
8a
26.529
f1 (ppm)

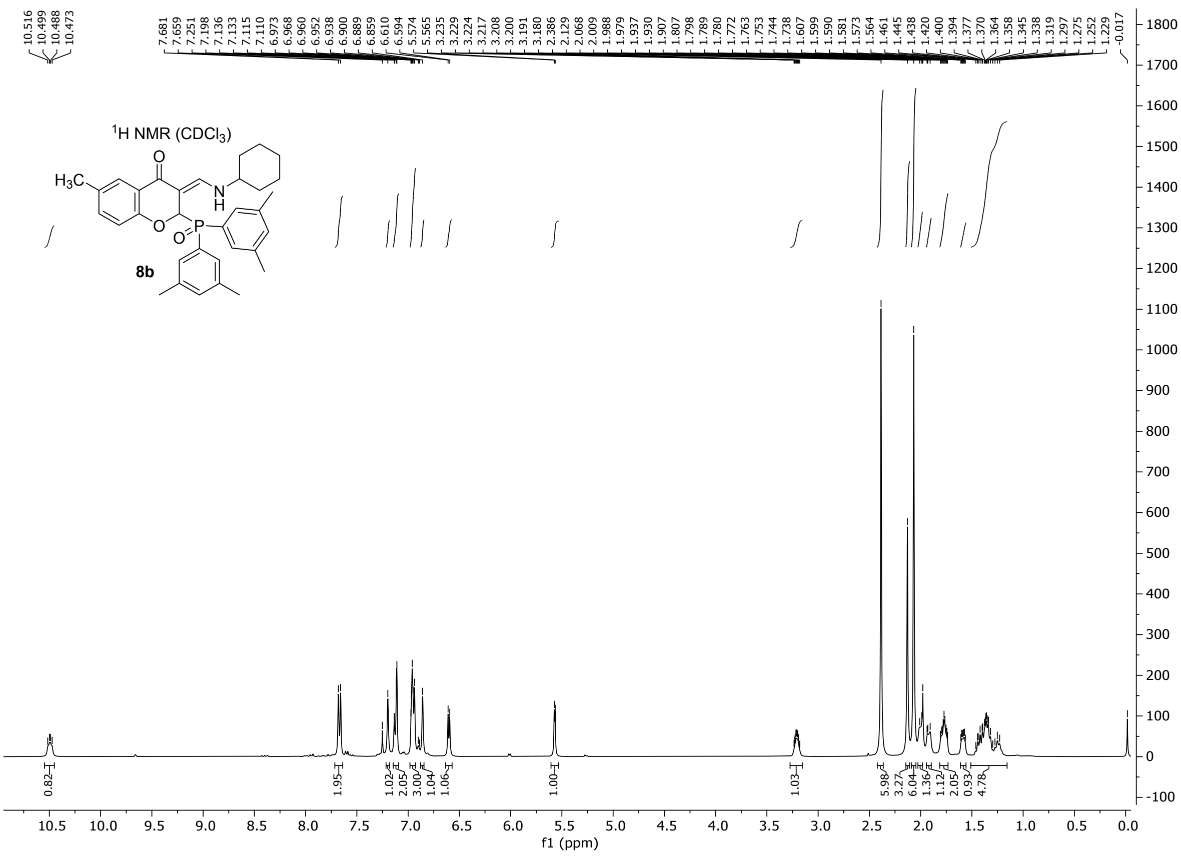

10.516
10.499
10.488
10.473
7.681
7.659
7.251
7.198
7.136
7.133
7.115
7.110
6.973
6.968
6.960
6.952
6.938
6.900
6.889
6.859
6.610
6.594
5.574
5.565
3.235
3.229
3.224
3.217
3.208
3.200
3.191
3.180
2.386
2.129
2.068
2.009
1.988
1.979
1.937
1.930
1.907
1.807
1.798
1.789
1.780
1.772
1.763
1.753
1.744
1.738
1.607
1.599
1.590
1.581
1.573
1.564
1.461
1.445
1.438
1.420
1.400
1.394
1.377
1.370
1.364
1.358
1.345
1.338
1.319
1.297
1.275
1.252
1.229
-0.017
¹H NMR (CDCl₃)
8b
f1 (ppm)

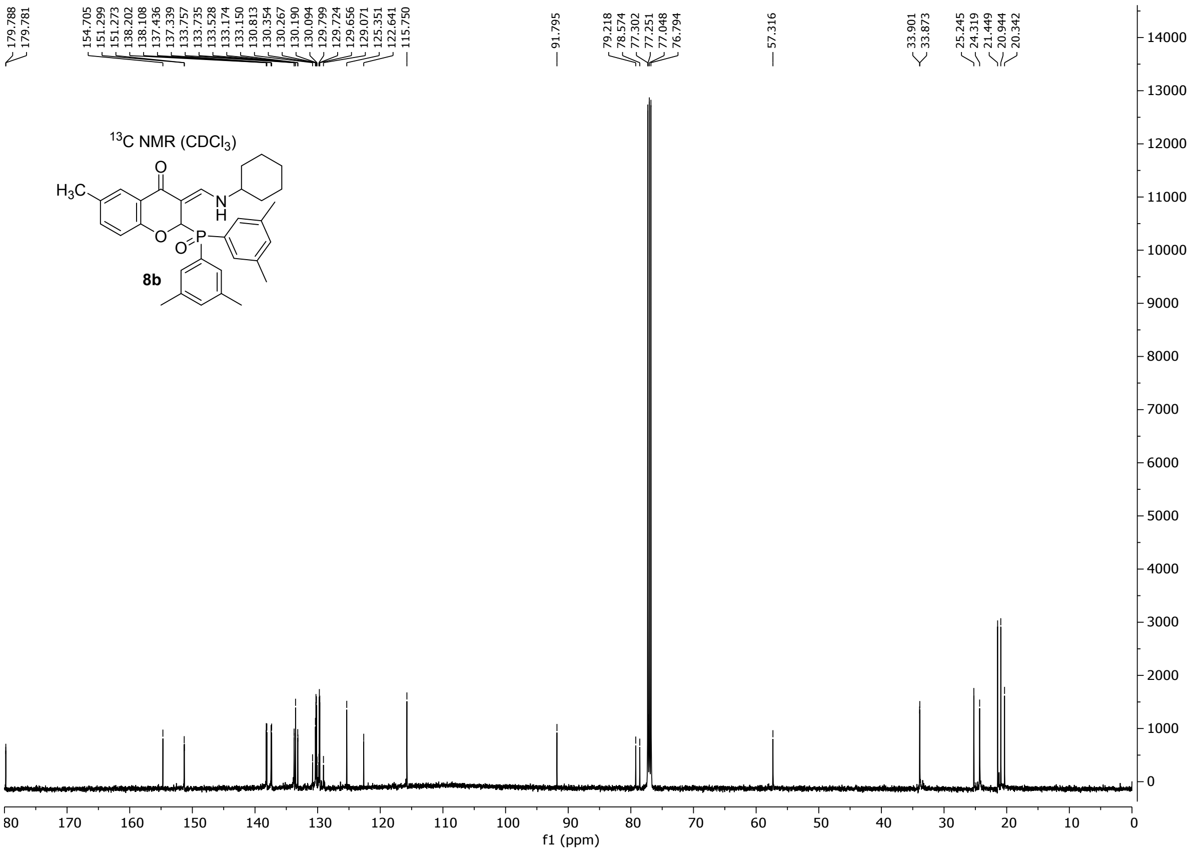

179.788
179.781
154.705
151.299
151.273
138.202
138.108
137.436
137.339
133.757
133.735
133.528
133.174
133.150
130.813
130.354
130.267
130.190
130.094
129.799
129.724
129.656
129.071
125.351
122.641
115.750
91.795
79.218
78.574
77.302
77.251
77.048
76.794
57.316
33.901
33.873
25.245
24.319
21.449
20.944
20.342
¹³C NMR (CDCl₃)
8b
f1 (ppm)

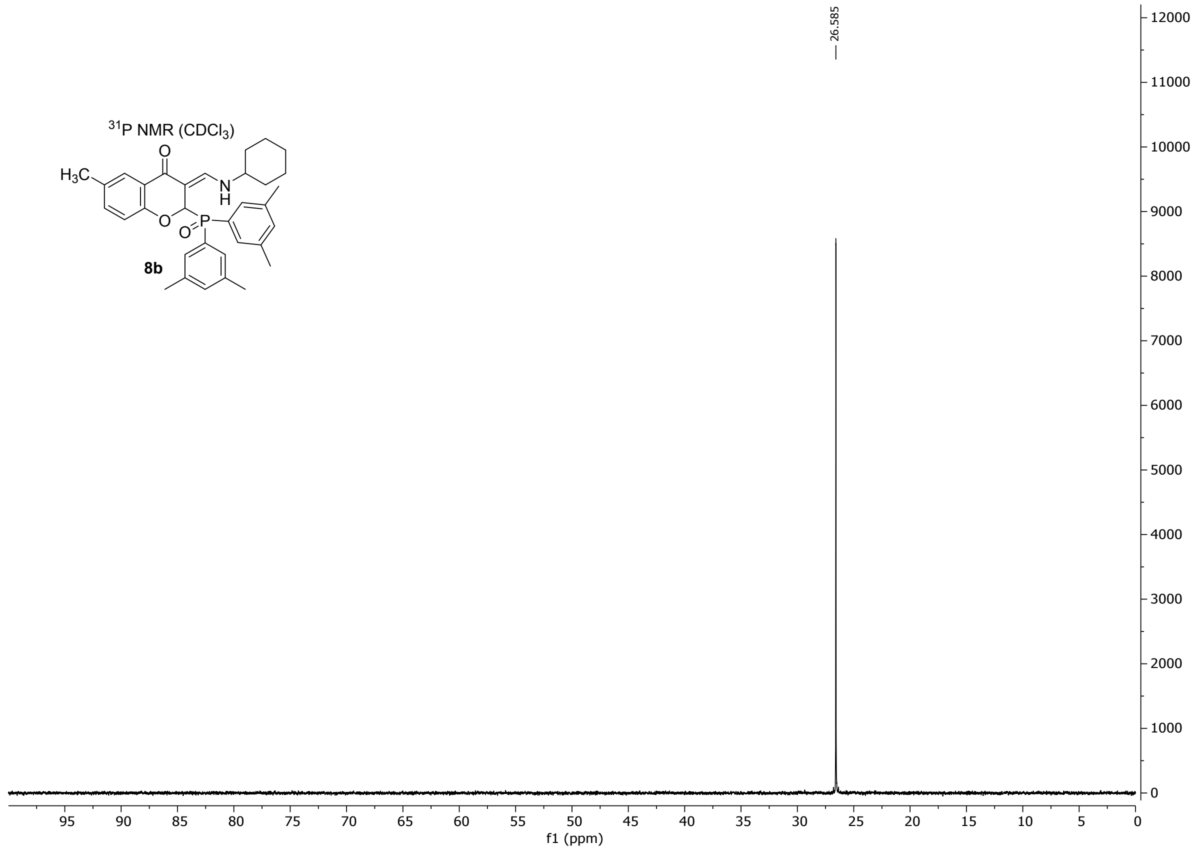

31P NMR (CDCl3)
8b
26.585
f1 (ppm)

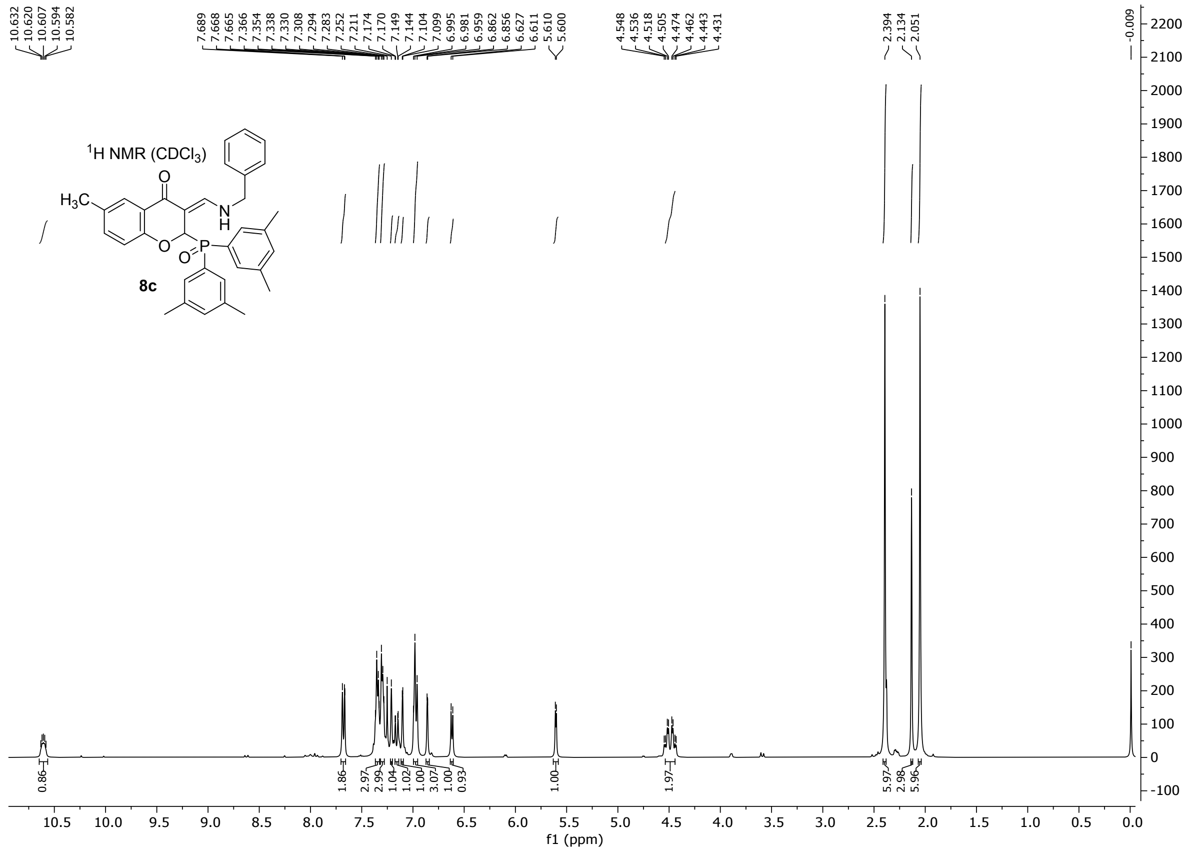

1H NMR (CDCl3)
8c
f1 (ppm)

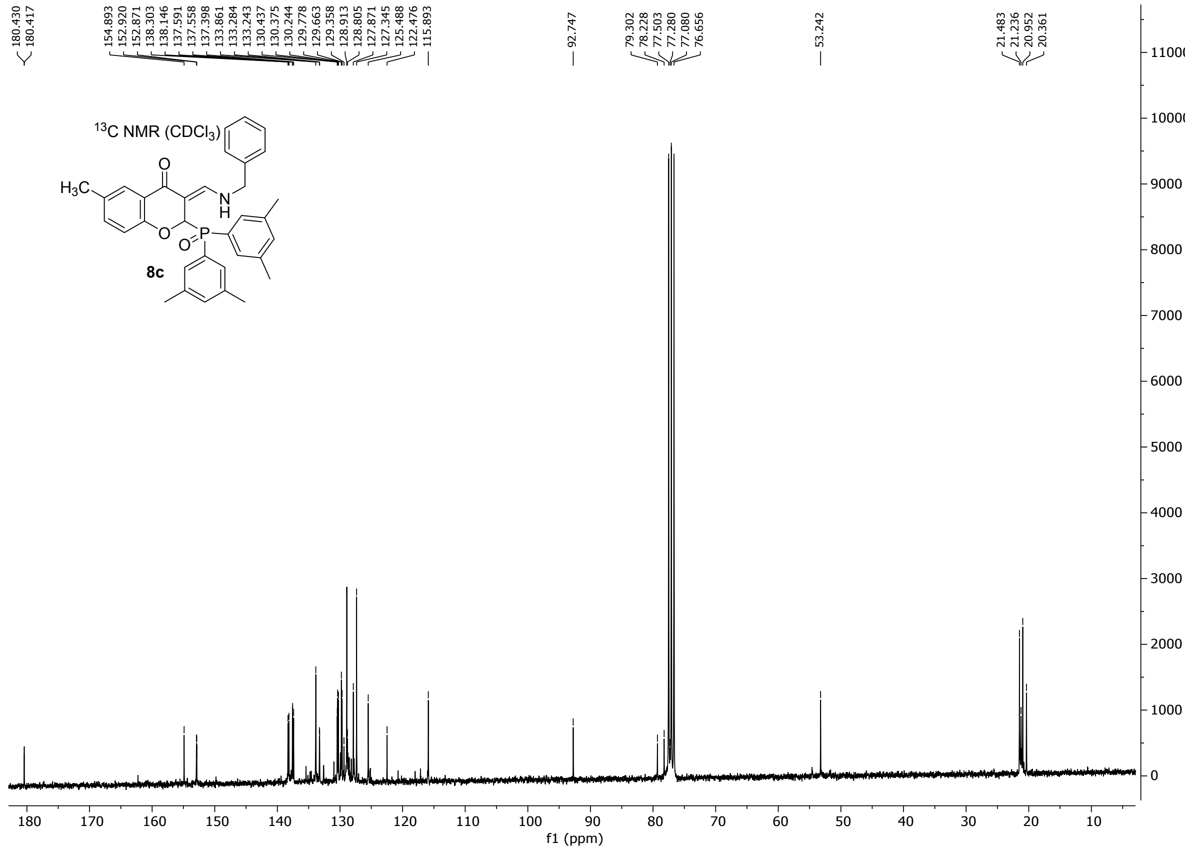

180.430
180.417
154.893
152.920
152.871
138.303
138.146
137.591
137.558
137.398
133.861
133.284
133.243
130.437
130.375
130.244
129.778
129.663
129.358
128.913
128.805
127.871
127.345
125.488
122.476
115.893
92.747
79.302
78.228
77.503
77.280
77.080
76.656
53.242
21.483
21.236
20.952
20.361
^{13}C NMR ($CDCl_3$)
O
H_3C
N
H
O
O
P
8c
f1 (ppm)

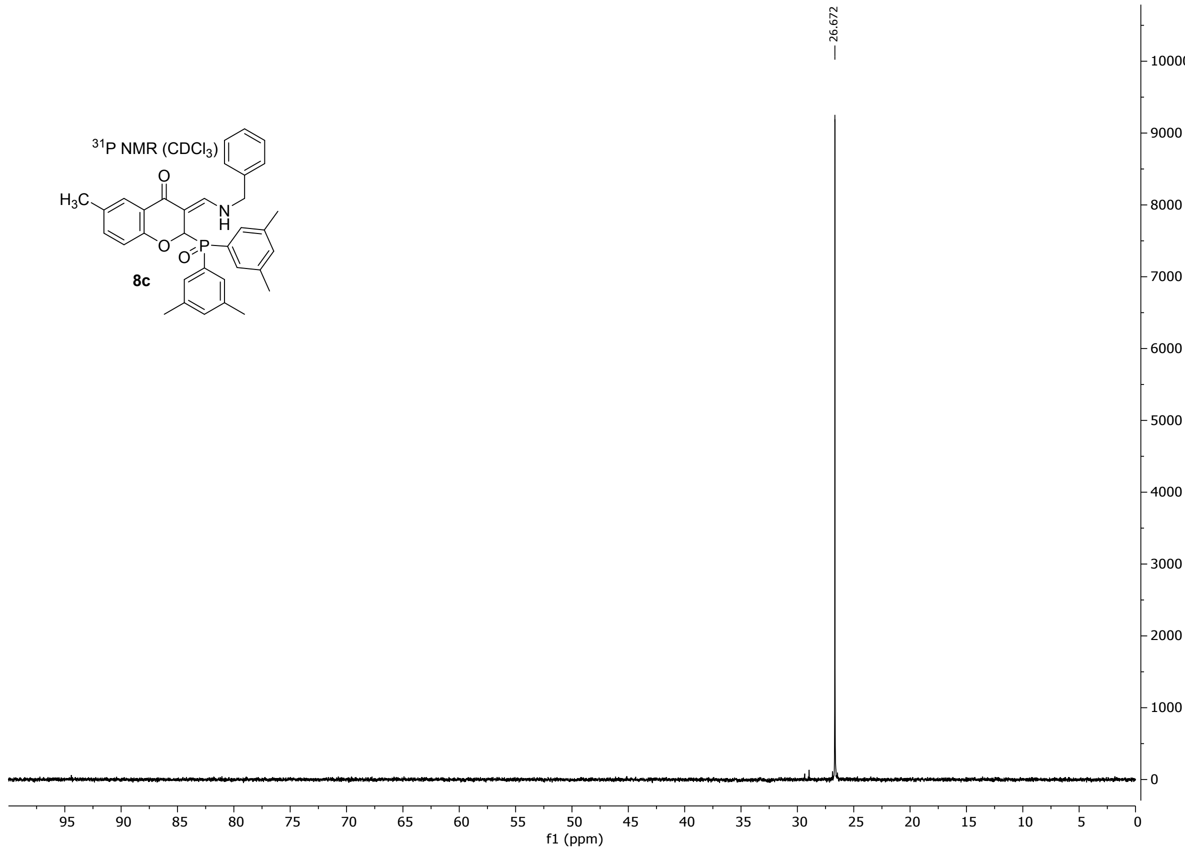

26.672
^{31}P NMR ($CDCl_3$)
O
H_3C
N
H
O
O
P
8c
f1 (ppm)

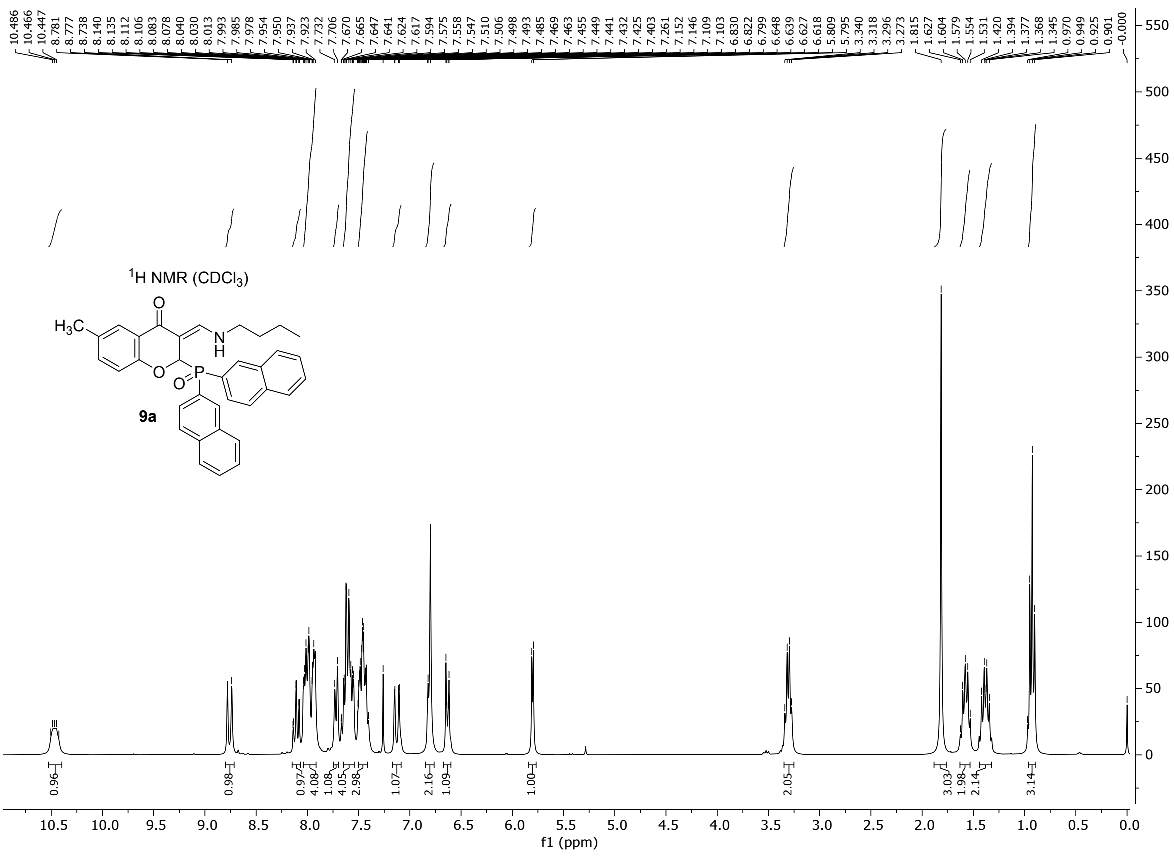
10.486 10.466 10.447 8.781 8.777 8.738 8.140 8.135 8.112 8.106 8.083 8.078 8.040 8.030 8.013 7.993 7.985 7.978 7.954 7.950 7.937 7.923 7.732 7.706 7.670 7.665 7.647 7.641 7.624 7.617 7.594 7.575 7.558 7.547 7.510 7.506 7.498 7.493 7.485 7.469 7.463 7.455 7.449 7.441 7.432 7.425 7.403 7.261 7.152 7.146 7.109 7.103 6.830 6.822 6.799 6.648 6.639 6.627 6.618 5.809 5.795 3.340 3.318 3.296 3.273 1.815 1.627 1.604 1.579 1.554 1.531 1.420 1.394 1.377 1.368 1.345 0.970 0.949 0.925 0.901 -0.000
^{1}H NMR (CDCl$_3$)
9a
0.96 0.98 0.97 4.08 1.08 4.05 2.98 1.07 2.16 1.09 1.00 2.05 3.03 1.98 2.14 3.14
f1 (ppm)

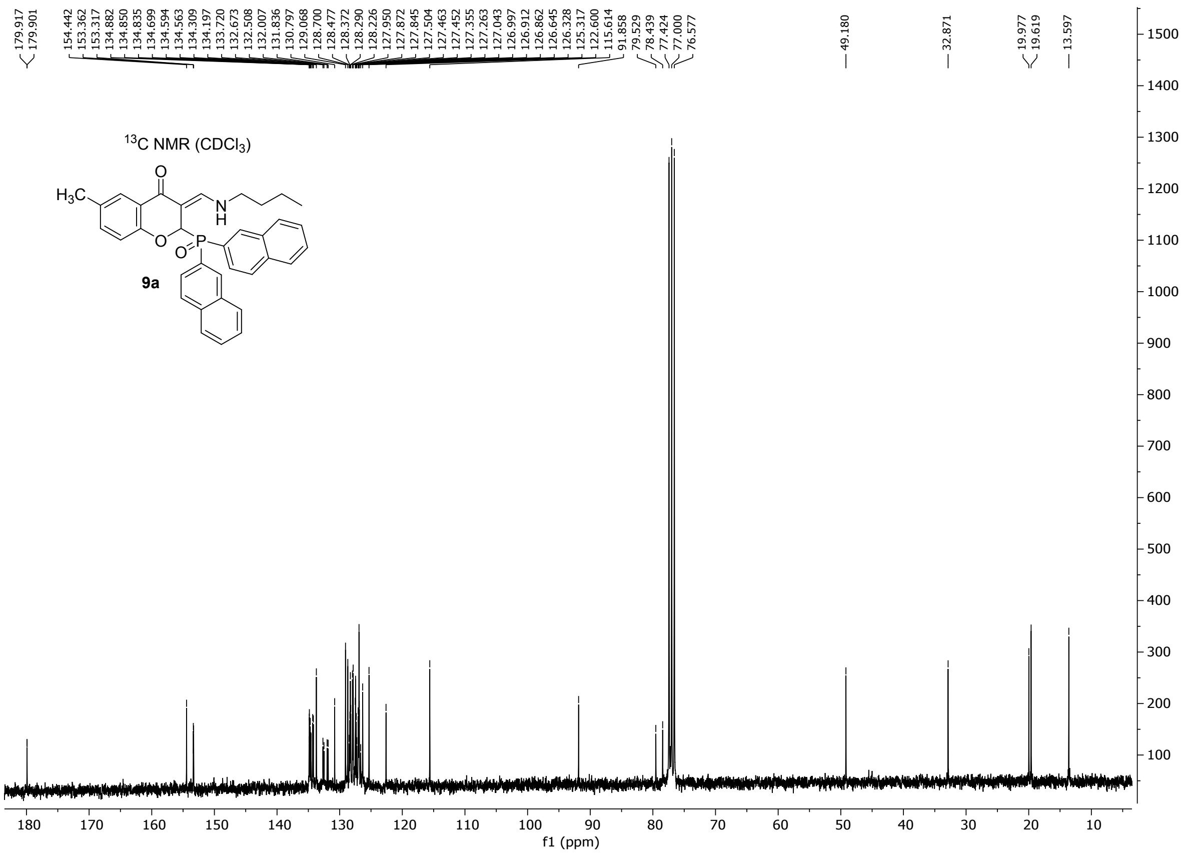
179.917 179.901 154.442 153.362 153.317 134.882 134.850 134.835 134.699 134.594 134.563 134.309 134.197 133.720 132.673 132.508 132.007 131.836 130.797 129.068 128.700 128.477 128.372 128.290 128.226 127.950 127.872 127.845 127.504 127.463 127.452 127.355 127.263 127.043 126.997 126.912 126.862 126.645 126.328 125.317 122.600 115.614 91.858 79.529 78.439 77.424 77.000 76.577 49.180 32.871 19.977 19.619 13.597
^{13}C NMR (CDCl$_3$)
9a
f1 (ppm)

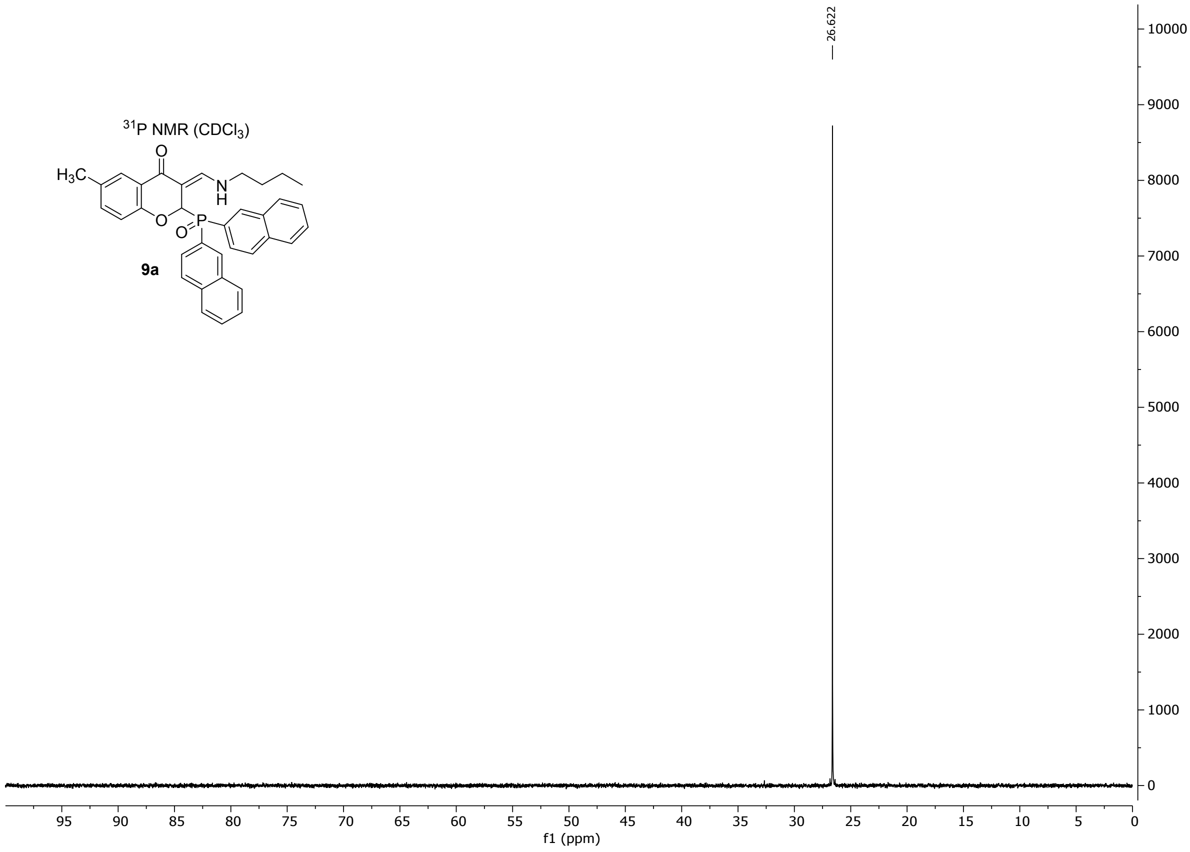
31P NMR (CDCl3)
9a
26.622
f1 (ppm)

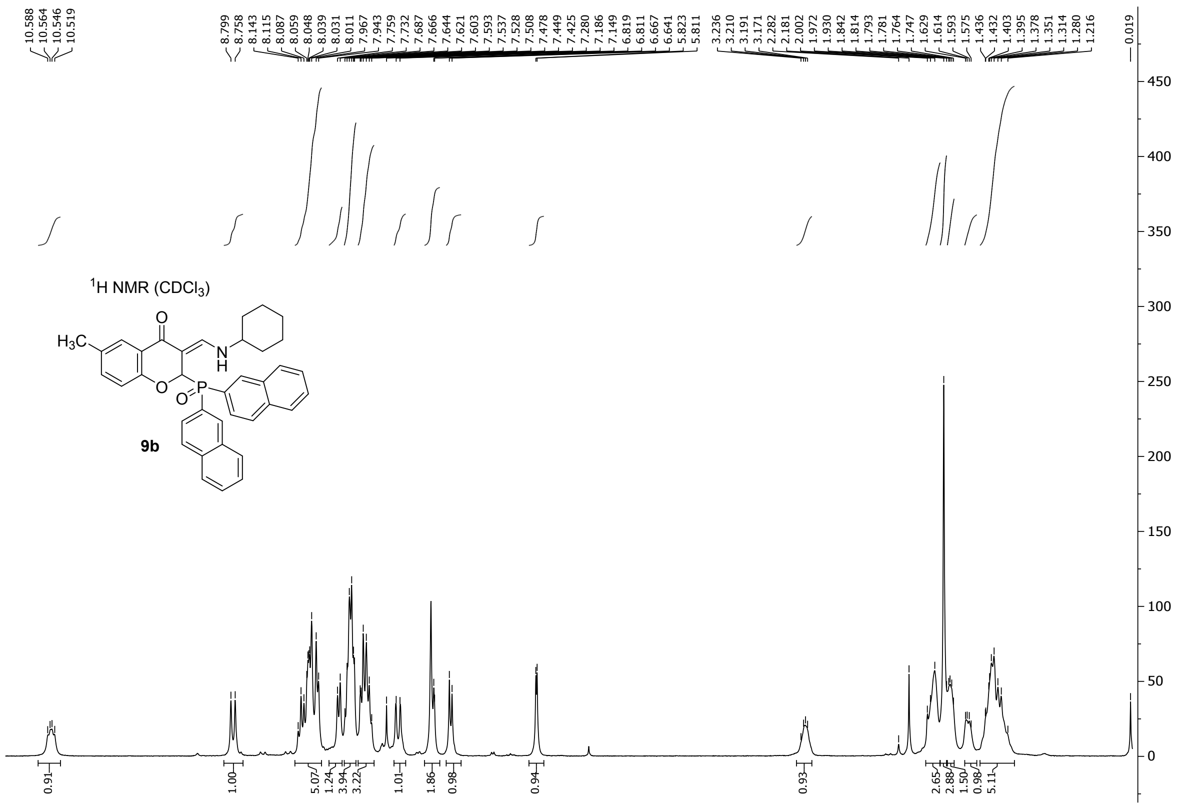
1H NMR (CDCl3)
9b
10.588
10.564
10.546
10.519
8.799
8.758
8.143
8.115
8.087
8.059
8.048
8.039
8.031
8.011
7.967
7.943
7.759
7.732
7.687
7.666
7.644
7.621
7.603
7.593
7.537
7.528
7.508
7.478
7.449
7.425
7.280
7.186
7.149
6.819
6.811
6.667
6.641
5.823
5.811
3.236
3.210
3.191
3.171
2.282
2.181
2.002
1.972
1.930
1.842
1.814
1.793
1.781
1.764
1.747
1.629
1.614
1.593
1.575
1.436
1.432
1.403
1.395
1.378
1.351
1.314
1.280
1.216
0.019
0.91
1.00
5.07
1.24
3.94
3.22
1.01
1.86
0.98
0.94
0.93
2.65
2.88
1.50
0.98
5.11
f1 (ppm)

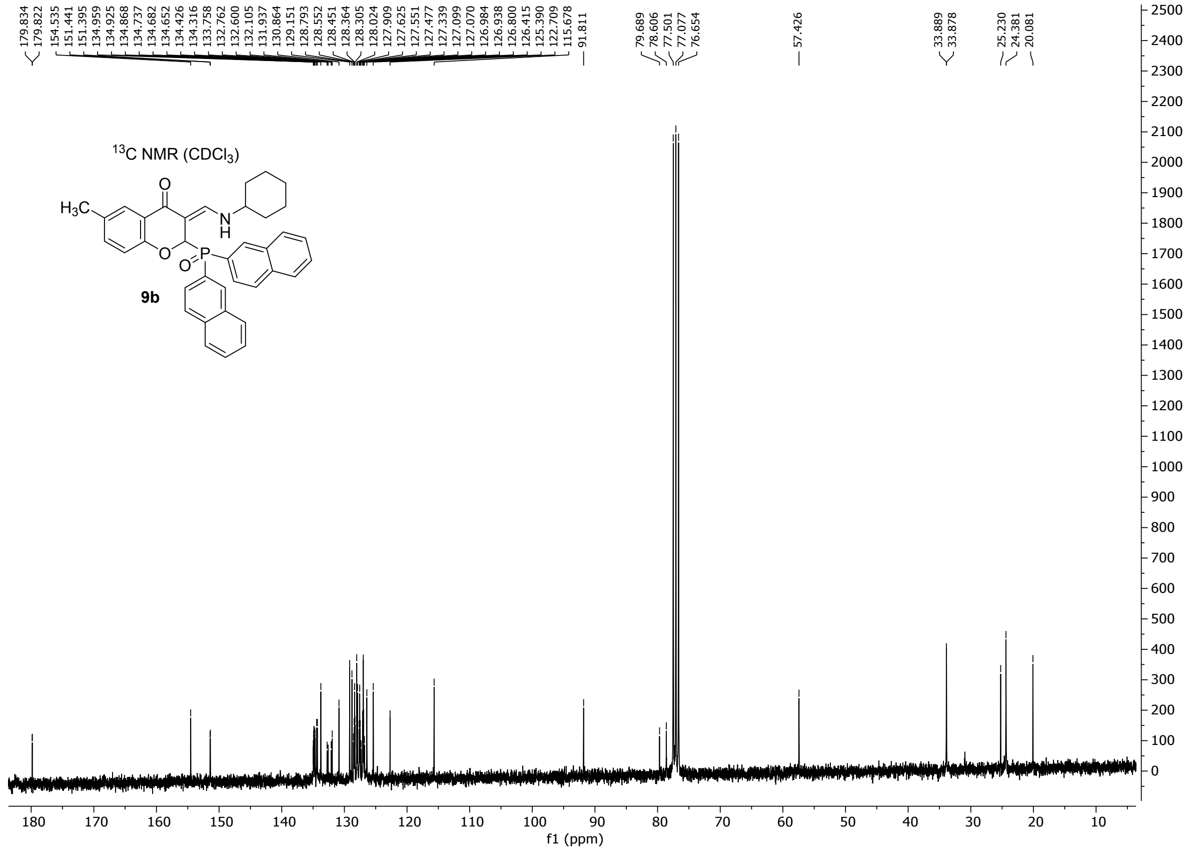

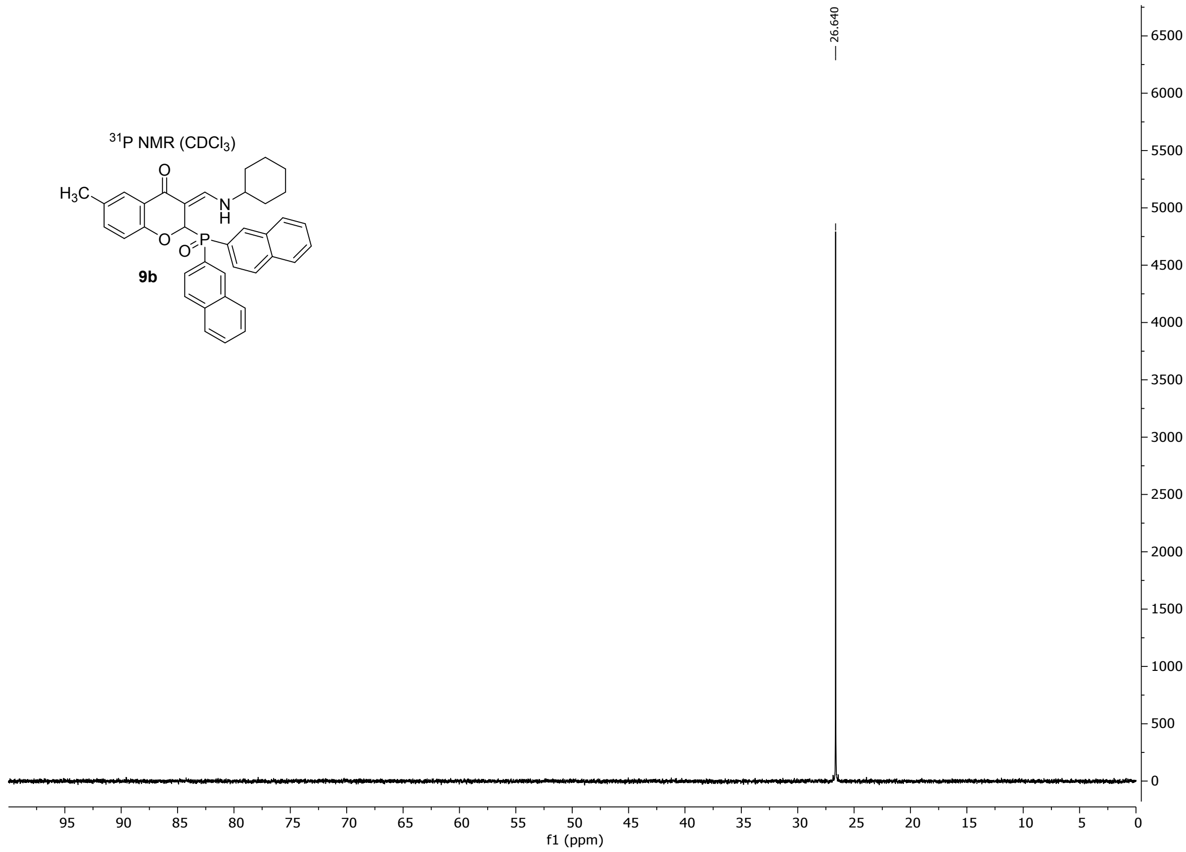

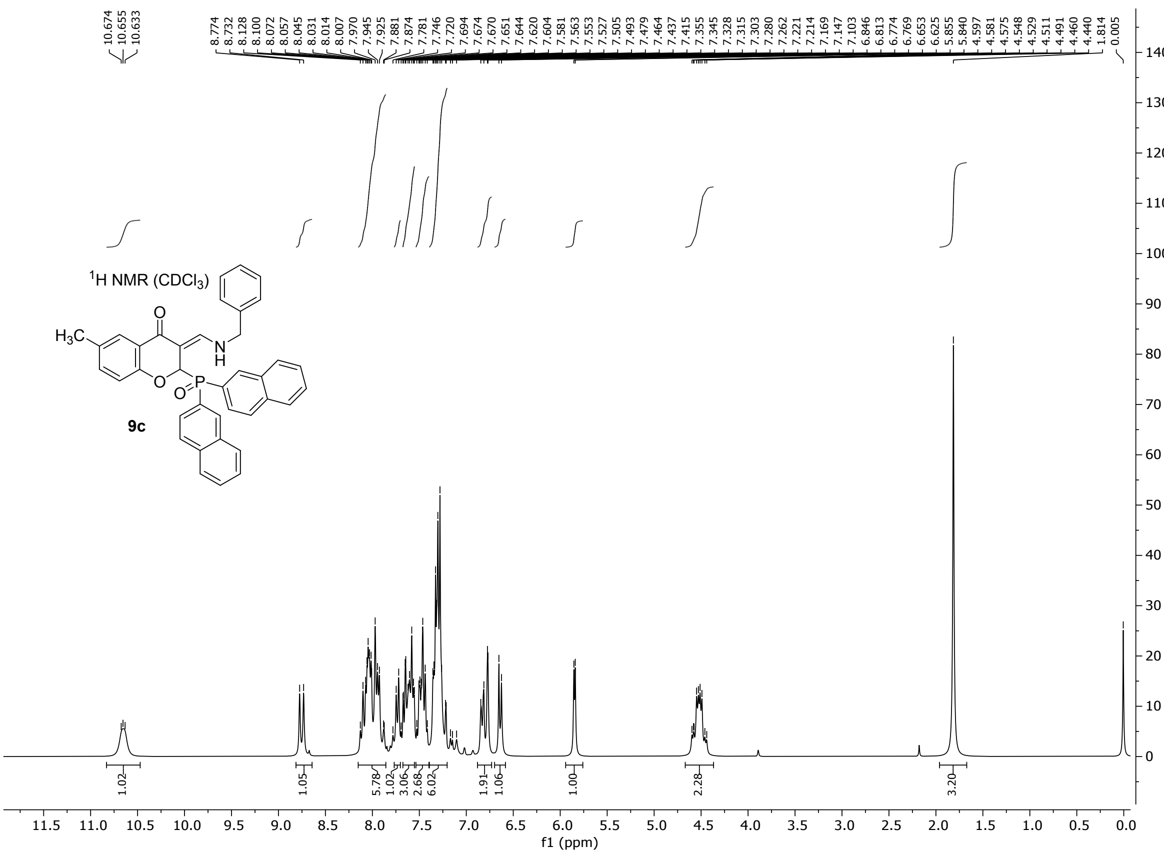

^{1}H NMR (CDCl$_3$)
9c
f1 (ppm)

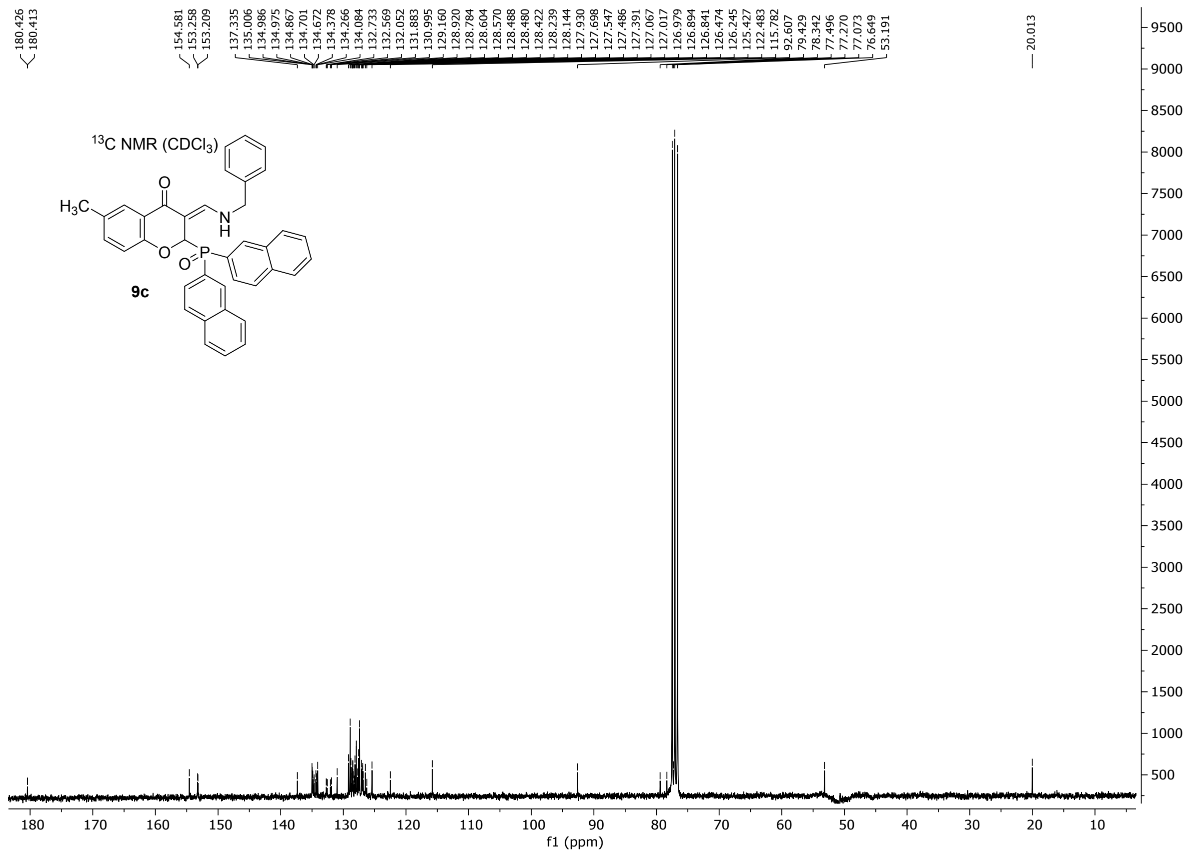

^{13}C NMR (CDCl$_3$)
9c
f1 (ppm)

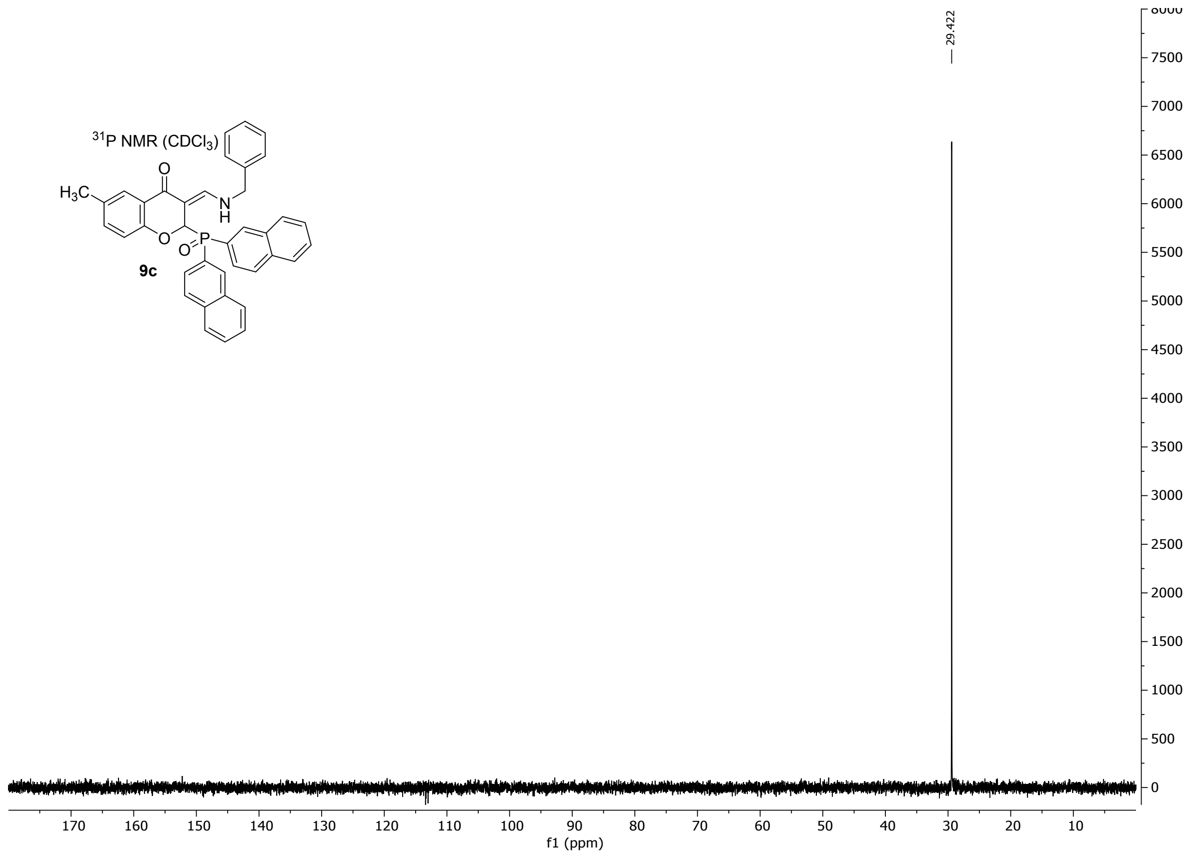
^{31}P NMR (CDCl$_3$)
9c
29.422
f1 (ppm)

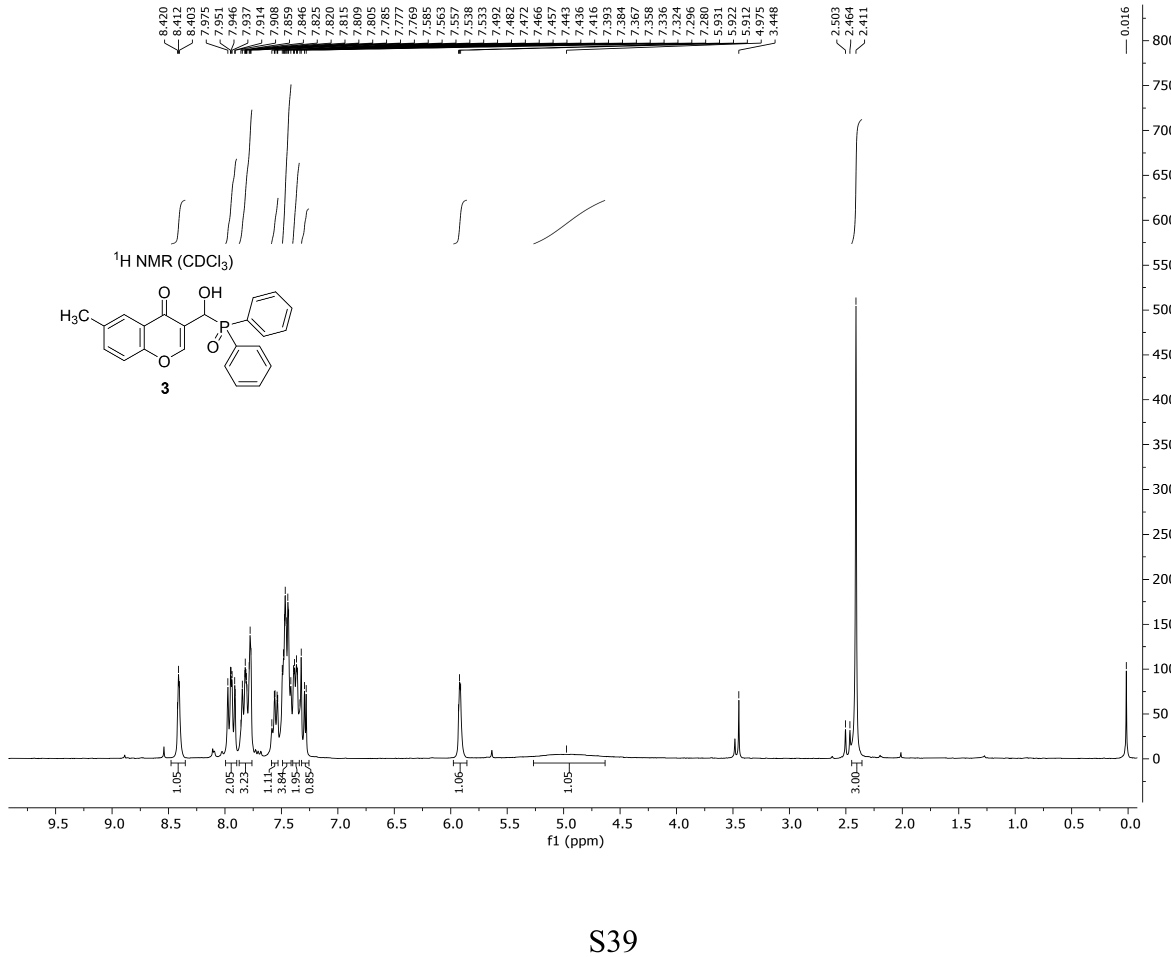
^{1}H NMR (CDCl$_3$)
3
f1 (ppm)

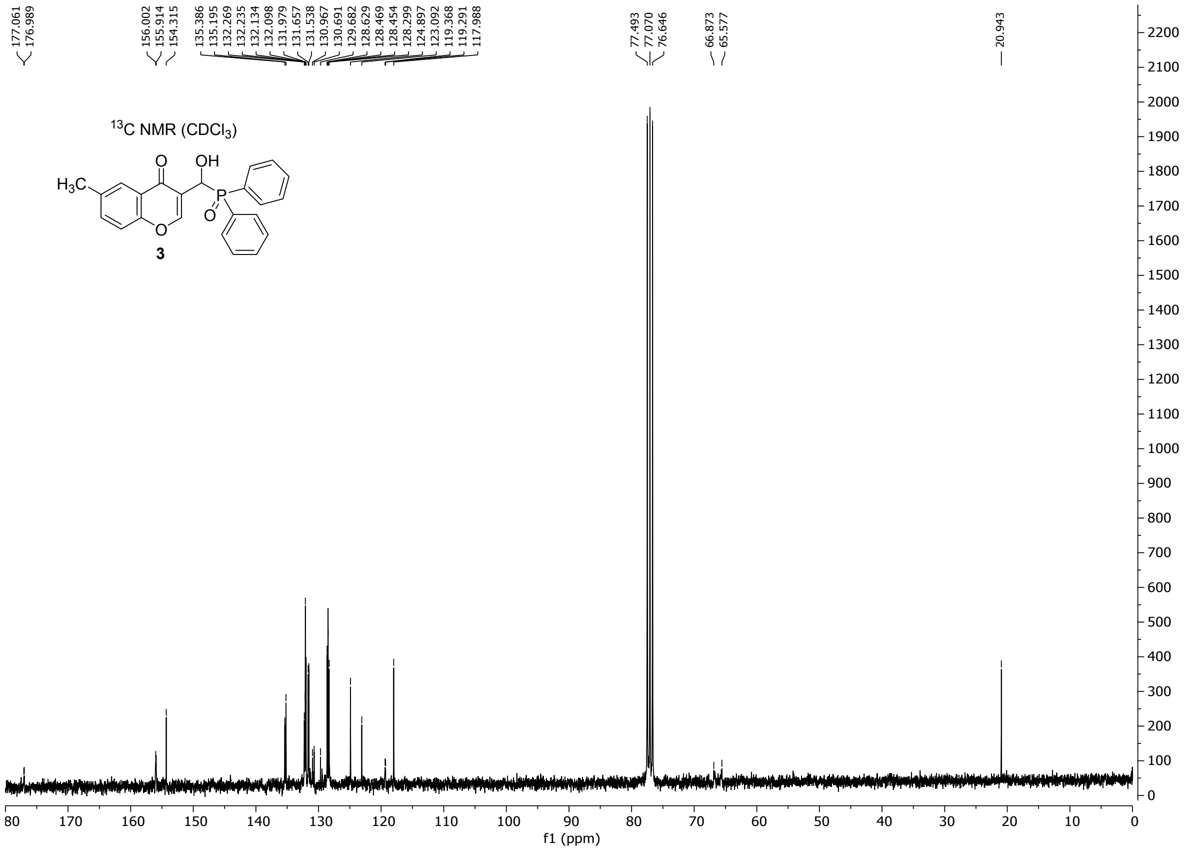
177.061
176.989
156.002
155.914
154.315
135.386
135.195
132.269
132.235
132.134
132.098
131.979
131.657
131.538
130.967
130.691
129.682
128.629
128.469
128.454
128.299
124.897
123.092
119.368
119.291
117.988
77.493
77.070
76.646
66.873
65.577
20.943
13C NMR (CDCl3)
O
OH
H3C
P
O
O
3
f1 (ppm)

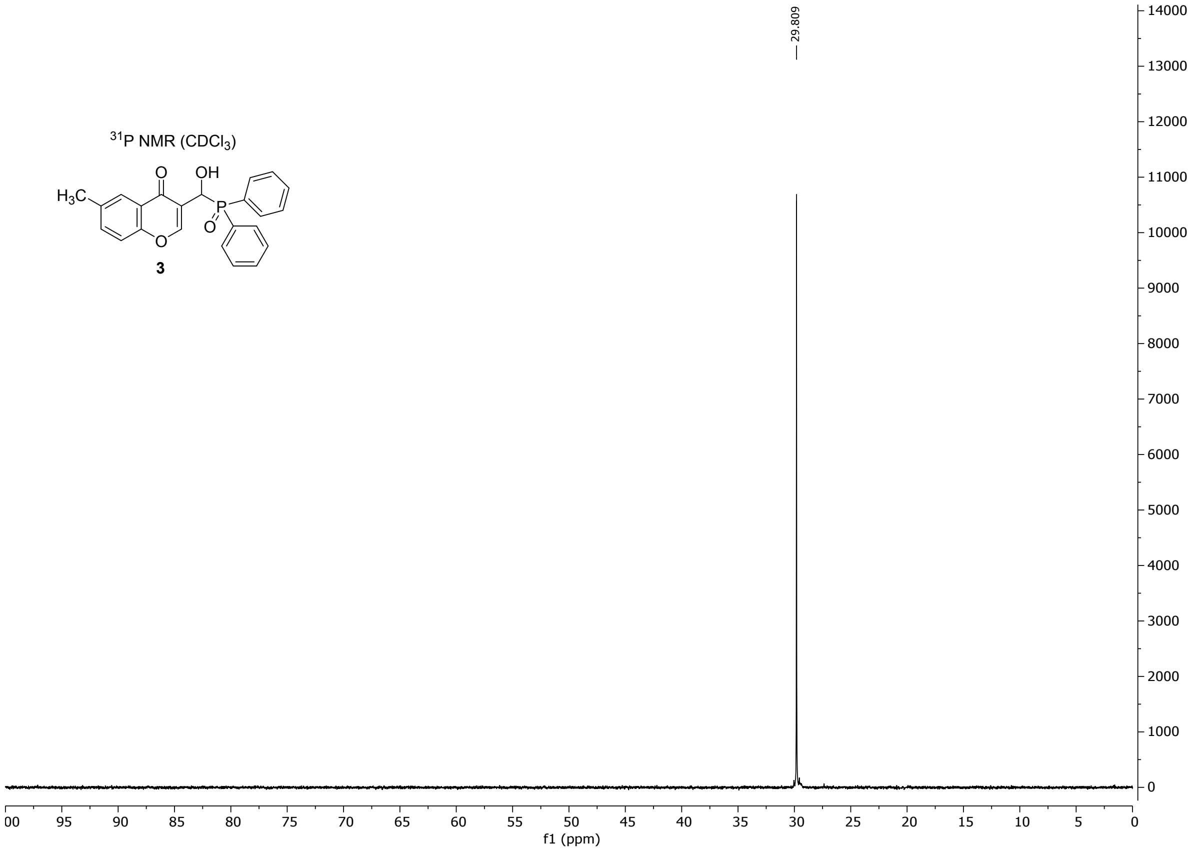
29.809
31P NMR (CDCl3)
O
OH
H3C
P
O
O
3
f1 (ppm)
